# Evaluating the Performance of Photon- and Electron-Based Fragmentation Methods in Omnitrap-LCMS Analysis of *N*‑Glycopeptides

**DOI:** 10.1021/acs.analchem.6c00435

**Published:** 2026-06-26

**Authors:** Nikita Levin, Daniel A. Polasky, Kai Li, Alexey I. Nesvizhskii, Shabaz Mohammed

**Affiliations:** † Rosalind Franklin Institute, Harwell Campus, Didcot OX11 0QX, U.K.; ‡ Department of Pharmacology, 6396 University of Oxford, Oxford OX1 3QT, U.K.; § Department of Pathology, 1259 University of Michigan, Ann Arbor, Michigan 48109, United States; ∥ Gilbert S. Omenn Department of Computational Medicine and Bioinformatics, University of Michigan, Ann Arbor, Michigan 48109, United States; ⊥ Department of Biochemistry, University of Oxford, Oxford OX1 3QU, U.K.; # Department of Chemistry, University of Oxford, Oxford OX1 3TA, U.K.

## Abstract

To date, collision-induced dissociation and methods based on electron transfer dissociation are considered the ‘best’ approaches for the mass spectrometry analysis of *N*- and *O*-glycopeptides, respectively, allowing for identification of both peptide and glycan compositions. In recent years, alternative fragmentation methods such as ultraviolet photodissociation (UVPD) and more energetic versions of electron-based techniques (e.g., electron ionization dissociation, EID) have been shown to be useful for analysis of glycopeptides, producing rich information on the glycopeptide structure, including types of glycosidic linkages. We evaluated UVPD, EID, electron capture dissociation (ECD), and activated-ion ECD (AI-ECD) using an Orbitrap–Omnitrap hybrid for LCMS analysis of complex *N*-glycopeptides. Both UVPD and EID generated extensive peptide, glycosidic, and cross-ring fragments, complementary to standard collisional dissociation, and achieved comparable identification efficiencies. While ECD alone produced few glycopeptide identifications, AI-ECD significantly improved yields through supplemental vibrational activation. These results establish the Omnitrap, and its ability to house multiple fragmentation approaches, as a powerful platform for comprehensive glycoproteomic analysis and highlight the continuing need for enhanced computational tools to interpret complex UVPD and EID spectra.

## Graphical abstract

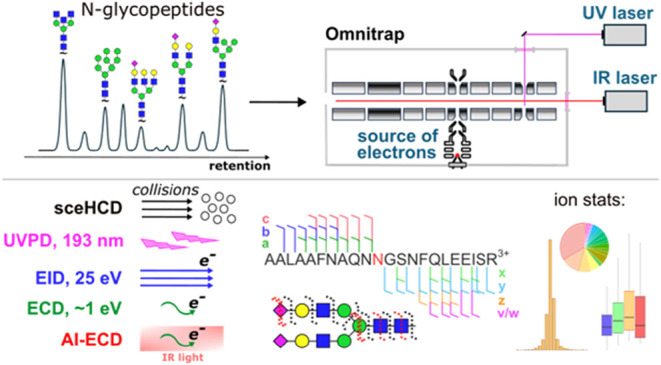

Protein glycosylation is a covalent attachment of an (oligo)­saccharide to specific amino acid residues within a polypeptide chain. Glycosylation is one of the most abundant and complex posttranslational modifications (PTMs) and plays a crucial role in multiple biological processes ranging from cell signaling and immune response to protein folding and protein stability.^1,2^ Glycoproteins and glycopeptides are often characterized by various degrees of glycosylation site occupancy (macroheterogeneity) and high diversity of glycan structure (microheterogeneity) including glycan composition, branching, and types of glycosidic bonds.^3,4^ This intricate nature of glycopeptides poses a significant challenge to the analysis of complex glycopeptide mixtures by conventional analytical techniques such as reverse-phase liquid chromatography coupled with mass spectrometry (RP-LCMS).^5–7^ In particular, collision-induced dissociation (CID) often fails to sufficiently characterize both peptide and glycan structures and firmly identify the glycosylation site due to high acid lability of glycosidic ether bonds resulting in their preferential dissociation in a typical CID experiment.^6^ To address this shortcoming, a variety of alternative fragmentation techniques have been suggested, which include methods based on the use of electrons of various energies,^8–20^ infrared radiation,^10,11,21^ ultraviolet photodissociation (UVPD),^22–24^ and combinations thereof.^25^ While electrons with relatively low energies (<5 eV) provide peptide fragments complementary to CID,^9–12,14,15^ high-energy electrons and UV light generate all types of peptide main-series fragments, dissociate glycosidic bonds, and produce cross-ring fragments of sugars.^16–18,22,24,26,27^ These fragments, when properly annotated, allow one to confirm the glycopeptide composition and may provide valuable information about the types of glycosidic linkages between saccharides which can be useful for unraveling their biological function.^28–30^ This level of information potentially enables direct analysis of glycosidic bonds by means of MS/MS sequencing of glycopeptides without the chemical derivatization of saccharides, which is currently the analytical method of choice.^31^

Despite ever-increasing attention from the glycopeptide community toward UVPD and electron-based dissociation techniques (ExD), literature providing fragmentation insight and utilization approaches, especially on the LCMS scale, is scarce, not least because of prohibitive requirements to instrumentation, (subsequent) lack of mechanistic understanding, and insufficient tools for data analysis. To address some of these issues, we have previously employed a hybrid Orbitrap-Omnitrap platform^32^ to characterize UVPD, EID, ECD, and activated-ion ECD (AI-ECD) in a series of direct-infusion experiments of a few standard polypeptides^33^ and LCMS analyses of a variety of highly complex peptide mixtures.^34^ In this paper, we extend our characterization of UVPD, EID, ECD, and activated-ion ECD (AI-ECD) to LCMS analyses of complex *N*-glycopeptide mixtures.

## Methods

### Materials

Two standard glycopeptides with amino acid sequences KVANKT and glycoforms A2G2S2 (HexNAc4Hex5NeuAc2) and FA2 (HexNAc4Hex3Fuc) were purchased from Ludger Ltd., see Supporting Information Figure S1d for detailed annotation of their structures. Human serum, solvents, and chemicals for sample preparation were obtained from Sigma-Aldrich. Trypsin was supplied by Promega, and LysC was purchased from Fujifilm Wako. Oasis HLB cartridges were acquired from Waters.

### Sample Preparation

Two standard purified glycopeptides were each resuspended in 50% acetonitrile in water with 0.1% formic acid for direct-infusion experiments. To prepare a complex mixture of glycopeptides enriched from a tryptic digest of human serum, we adopted the protocol suggested by Takakura et al.^35^ Briefly, 1 μL of human serum was dissolved in 100 mM ammonium bicarbonate containing 8 M urea, reduced by 10 mM TCEP and alkylated by 50 mM 2-CAA, diluted to 6 M urea, incubated with 2 μg of LysC for 4 h at 37 °C, further diluted to 1 M urea, and incubated with 2 μg of trypsin at 37 °C overnight. After incubation, 5-fold volume of ice-cold acetone was added to the peptide mixture and left at −20 °C overnight. The resulting mixture was centrifuged at 12000 g, and the supernatant was carefully aspirated and discarded. The pellet containing glycopeptides was redissolved in 5% formic acid in water, desalted using Oasis HLB cartridges and resuspended in 5% formic acid 5% DMSO prior to LCMS analysis.

### Direct Infusion and LCMS Analyses

All experiments were performed on a Thermo Fisher Scientific Exploris Orbitrap 480 mass spectrometer modified with an Omnitrap platform. The design of direct-infusion experiments was described in the previous publication.^33^ Briefly, precursor ions were isolated in the quadrupole mass filter using an isolation mass window of 2 Th, processed in the Omnitrap, and fragments together with unfragmented precursor ions were characterized in the Orbitrap analyzer with a mass resolution of 60,000. The injection times were fixed and set to match the AGC target of 200,000. An ArF ExciStar 200 laser (Coherent, Santa Clara, CA) was used as the source of 193 nm UV light. A FireStar ti60 (Synrad, Mukilteo, WA) laser was used as the source of 10.6 μm IR light with a maximum power output of 60 W. For analysis of complex mixtures, samples were subjected to LCMS/MS using an UltiMate 3000 nanoUHPLC system (Thermo Fisher Scientific) coupled to an Exploris-Omnitrap hybrid instrument. The peptides were trapped on a C18 PepMap100 precolumn (300 μm i.d. × 5 mm, 100 Å, Thermo Fisher Scientific) using solvent A (0.1% formic acid in water), then separated on an in-house packed analytical column (50 μm i.d. × 50 cm in-house packed with ReproSil Gold 120 C18, 1.9 μm, Dr. Maisch GmbH) with a gradient of 5% to 45% B (0.1% formic acid in acetonitrile) over 60 min at a flow rate of 100 nL/min. Full scan MS1 survey spectra were acquired in the Orbitrap (scan range 400–2000 *m*/*z*, resolution 60,000, AGC target 1,200,000). The 20 most intense peaks identified in a survey scan were selected for fragmentation in the Omnitrap with an AGC target of 200,000 and a maximum injection time of 60 ms, and fragments together with unfragmented precursor ions were characterized in the Orbitrap analyzer in a single scan with a mass resolution of 45,000. SceHCD experiments were performed in the HCD cell of Exploris at 20, 30, and 40% of normalized collision energy (NCE) using an AGC target of 100,000, a maximum injection time of 60 ms, and a mass resolution of 30,000. The first mass in MS2 scans was locked at 125 *m*/*z* in all experiments. The low-mass cutoff of the Omnitrap was held constant at 150 *m*/*z* during acquisition.

### Data Analysis

Raw LCMS data were analyzed in the FragPipe 23.1 platform using the MSFragger 4.3 search engine^36^ featuring a glycopeptide search module.^37^ The search was performed using “glyco-N-HCD” or “glyco-N-Hybrid” default parameters with, depending on the purpose of the search, various fragment ion series specified for identification in the “MSFragger” tab. Variable modifications included oxidation of methionine and protein *N*-terminal acetylation, and cysteine carbamidomethylation was set as fixed modification. Glycans were searched within the “Human_N-glycan_Medium” database. Results were filtered to 1% false discovery rate on both peptide and glycan levels.^38,39^ Downstream data processing was performed using in-house R scripts (RStudio build 418, R version 4.3.1).

### Spectral Annotations and Extraction of Fragment Ion Intensities

Automated annotations of peptide- and glycan- fragment ions in LCMS spectra were performed using GlyCounter^40^ v1.0.19 and a new fragment annotation tool available in the custom prerelease FragPipe 24.1-build 17. GlyCounter parameters are given in Supporting Information Figure S2. FragPipe’s annotator outputs experimental *m*/*z* values, intensities and mass errors of peptide-, oxonium-, and *Y*-type fragment ions. Selected spectra were manually annotated using fragment *m*/*z* values generated *in-silico* in Protein Prospector v6.5.0 (http://prospector.ucsf.edu) and GlycoWorkbench v1.2.4105.^41^ Nomenclature for the annotation of spectra used in this paper is given in Supporting Information Figure S1.

## Results and Discussion

### Initial Characterization of ExD and UVPD of Standard Glycopeptides in Direct-Infusion Experiments

The distinct fragmentation behavior of glycopeptides required its own preliminary investigation using an analyte with known composition of amino acid and glycan sequences. We started with the study of standard glycopeptides KVANKT modified with glycoforms HexNAc4Hex5NeuAc2 or HexNAc4Hex3Fuc (Supporting Information Figure S1d) in direct-infusion ECD, AI-ECD, UVPD, and EID experiments. ECD of triply charged precursor generated very clear spectra dominated by charge-reduced precursor ions and losses of N-acetyl and sialic acid groups (Supporting Information Figure S3a). In addition, relatively low-abundant *c* and *z* ions formed a complete coverage of the peptide sequence and were able to retain the intact glycan. Among other products, we identified one oxonium ion at *m*/*z* 657, which together with the loss of sialic acid resulted in only two dissociated glycosidic bonds. ECD of the doubly charged precursor generated only two *c* ions (Supporting Information Figure S3b) reflecting its well-documented inefficiency for 2+ precursors.^34,42^ The extent of fragmentation of glycosidic bonds is modest since low-energy electrons dissociate primarily C_α_-N bonds within the amino-acid backbone and add very little vibrational energy.^43^ Coon and co-workers have shown that supplemental coactivation by IR radiation can be beneficial for ETD analysis of glycopeptides (AI-ETD).^25^ We reasoned that, similar to that study, the combination of ECD and IRMPD within one MS scan (activated-ion ECD, AI-ECD^44,45^ ) may enhance fragmentation by providing glycan fragments and at the same time improving the amino acid sequence coverage by *c* and *z* fragments via vibrational activation of charge-reduced products. To achieve the best results, the energy supplied by the IR laser must be sufficiently low to minimize secondary fragmentation of *c,z* ions generated by ECD. To facilitate efficient AI-ECD, we therefore moved the IR laser focus out of the ECD reaction chamber by adjusting the optical lens and limited the laser coirradiation time to 50 ms at 15% laser duty cycle while keeping the duration of the electron irradiation at 150 ms. Coirradiation by low-energy IR photons led to the more extensive desialylation of *c* and *z* fragments and the generation of oxonium and *Y* fragments (Supporting Information Figure S4).

UVPD and EID experiments both generated an abundance of fragments of different types, including peptide, glycosidic, and cross-ring fragments. When comparing the glycoforms, we observed that the glycan composition affects the sequencing of the peptide: the presence of additional galactose and sialic acid saccharides on both antennas reduced the number of peptide backbone fragments, in particular on the C-terminus irrespective of applied conditions (Supporting Information Figures S5 and S6). Interestingly, it was galactose and sialic acid that demonstrated extensive cross-ring dissociation (*A*- and *X*-types of glycan fragments) in both EID and UVPD, while the HexNAc4Hex3Fuc glycoform showed almost exclusively ^1,5^X fragments of various saccharides, which is simply the combination of an anomeric carbon and one oxygen atom. While UVPD and EID indeed produced a plethora of cross-ring and glycosidic internal glycan fragments, many of them (labeled with asterisks in Supporting Information Figures S5–S8) have identical masses, which makes it impossible to determine their identity using standard analytical workflows. In addition, we discovered some peaks that were present in the spectra of both glycoforms of the standard glycopeptides but could only be annotated for one of them. For example, Supporting Information Figure S9 shows EID and UVPD spectra of standard glycopeptides zoomed-in around the *m*/*z* 382.134. While it is tempting to assign this peak to the [^0,3^A_Gal_+NeuAc] fragment in the spectrum of the HexNAc4Hex5NeuAc2 glycopeptide, the question remains about its identity in the spectrum of the HexNAc4Hex3Fuc glycoform. The solution we propose is to consider secondary cross-ring fragmentation. This way, the peak can be assigned to [^0,3^A_Man_+Man+^3,5^X_GlcNAc_] or [^1,4^A_Man_+Man+^3,5^X_GlcNAc_] (Supporting Information Figure S9). We acknowledge, however, that such a solution will further complicate an already complex analysis of glycopeptide spectra.

The charge state had hardly any effect on the fragment generation (Supporting Information Figures S7 and S8), possibly suggesting a charge-remote mechanism of dissociation. In terms of fragment diversity, EID produced noticeably more cross-ring fragments, while UVPD performed slightly better at dissociating the peptide backbone. Importantly, both techniques cleaved all glycosidic bonds, producing complete sequences of Y fragments for both glycoforms and retaining the core fucose in the HexNAc4Hex3Fuc. To determine the effect of the experimental parameters on the fragmentation efficiency, we plotted the absolute intensities of representative peptide, glycosidic, and cross-ring fragments against the number and energy of laser pulses in UVPD (Supporting Information Figures S10 and S11) and irradiation length in EID (Supporting Information Figure S12). We observed a continuous increase in fragmentation intensity within the range of chosen values. It is noteworthy that the nonmodified peptides experienced optimal fragmentation intensity well within this range of values.^34^ This suggests that higher energy density may be required to dissociate glycopeptides compared to nonmodified peptides, but further investigation with a larger pool of glycopeptides is necessary. Reassuringly, our direct-infusion EID of the HexNAc4Hex5NeuAc2 glycopeptide and those by Wei and co-workers^18^ produced similar spectra, suggesting that EID on an Omnitrap can be reproducible and independent of the mass analyzer.

### Characterization of ExD and UVPD in LCMS Experiments

In order to characterize ECD, AI-ECD, UVPD, and EID on a larger range of glycopeptides, we extended our study to LCMS analysis of complex mixtures. We generated a tryptic digest of human serum that had its glycopeptide population enriched by acetone precipitation.^35^ Such treatment preferentially removes hydrophobic compounds, yielding a mixture of mainly hydrophilic peptides and *N*-glycosylated peptides. The choice of the search engine for LCMS data analysis was dictated, first, by the need to control false discovery rate (FDR) on both peptide and glycan levels and, second, by the need to be able to freely select the types of peptide fragment ions for analysis of EID and UVPD data. We therefore opted for the MSFragger search engine^36^ implemented as part of the FragPipe proteomics platform. FragPipe provides a tool for glycopeptide analysis via its MSFragger-Glyco module,^37^ enables glycan FDR control,^39^ and allows users to customize peptide fragment ion types used for identification.

We first performed a stepped high-energy collisional dissociation (sceHCD) analysis of the glycopeptide mixture at 20, 30 and 40% normalized collisional energy and searched for *N*-glycopeptides. This method reflects a common approach to study *N*-glycopeptides and served as a benchmark for the other types of glycopeptide dissociation. In this experiment, we identified 4314 nonglycopeptide spectrum matches (nonglycoPSMs), 2104 glycoPSMs, 2007 unique nonglycosylated peptides, and 1256 unique glycopeptides which equates to 33% enrichment on the (glyco)­PSM level and 38% on the unique (glyco)­peptide level. In line with earlier studies,^46,47^ the distribution of precursors *m/z-*binned values showed an apparent separation between the two groups where glycopeptide precursors were shifted toward higher *m*/*z* (Supporting Information Figure S13).

We then investigated the efficiency of the ECD in the analysis of the glycopeptide mixture. In total, 2025 PSMs were identified in an ECD experiment using 100 ms of electron irradiation and *c*- and *z*-ions for analysis, with only 131 of them being glycoPSMs. The effect of ion activation induced by IR light is demonstrated in Figure 1, showing ECD and AI-ECD spectra of the peptide [QQQHLFGSNVTDCSGNFCLFR]^3+^ modified with a complex glycan [HexNAc4Hex5NeuAc2]. ECD resulted in only six peptide fragments (see Supporting Information Figure S14 for the definitions of peptide *d*-, *v*-, and *w*-ion types) and two glycan fragments of the precursor (Figure 1a), while AI-ECD yielded a near-complete peptide sequence coverage and dissociates four glycosidic bonds within the glycan (Figure 1b). Interestingly, the AI-ECD spectrum contains many *b,y* peptide fragments (Supporting Information Figure S15), which is not typical of the low-energy IRMPD of glycans.^10,11,21^ In total, we identified 473 glycoPSMs in AI-ECD experiments, a significant improvement over ECD only. Studying the distribution of precursors (Figure 1c) showed that the additional vibrational energy only slightly improved the identification rate at smaller *m*/*z* bins but greatly enhanced the numbers at higher *m*/*z*. The IR activation appears to be the most productive for 3+ and 4+ glycopeptide precursors, as very few doubly charged precursors were identified in both ECD and AI-ECD (Figure 1d–g). An extensive loss of an acetyl group from charge-reduced species, competing with the formation of informative fragments such as *c,z* ions, could be observed in some ECD mass spectra demonstrated in this study (e.g., Supporting Information Figures S3 and S4). An attempt was made previously to describe the loss of an acetyl group in terms similar to ECD of nonmodified peptides.^8^ The proposed mechanism involves an attack of the N-acetyl nitrogen atom in a glycan by a hydrogen atom liberated in ECD and a formation of an N-centered hypervalent radical which subsequently fragments by loss of an acetyl radical.^8^

**Figure 1:**
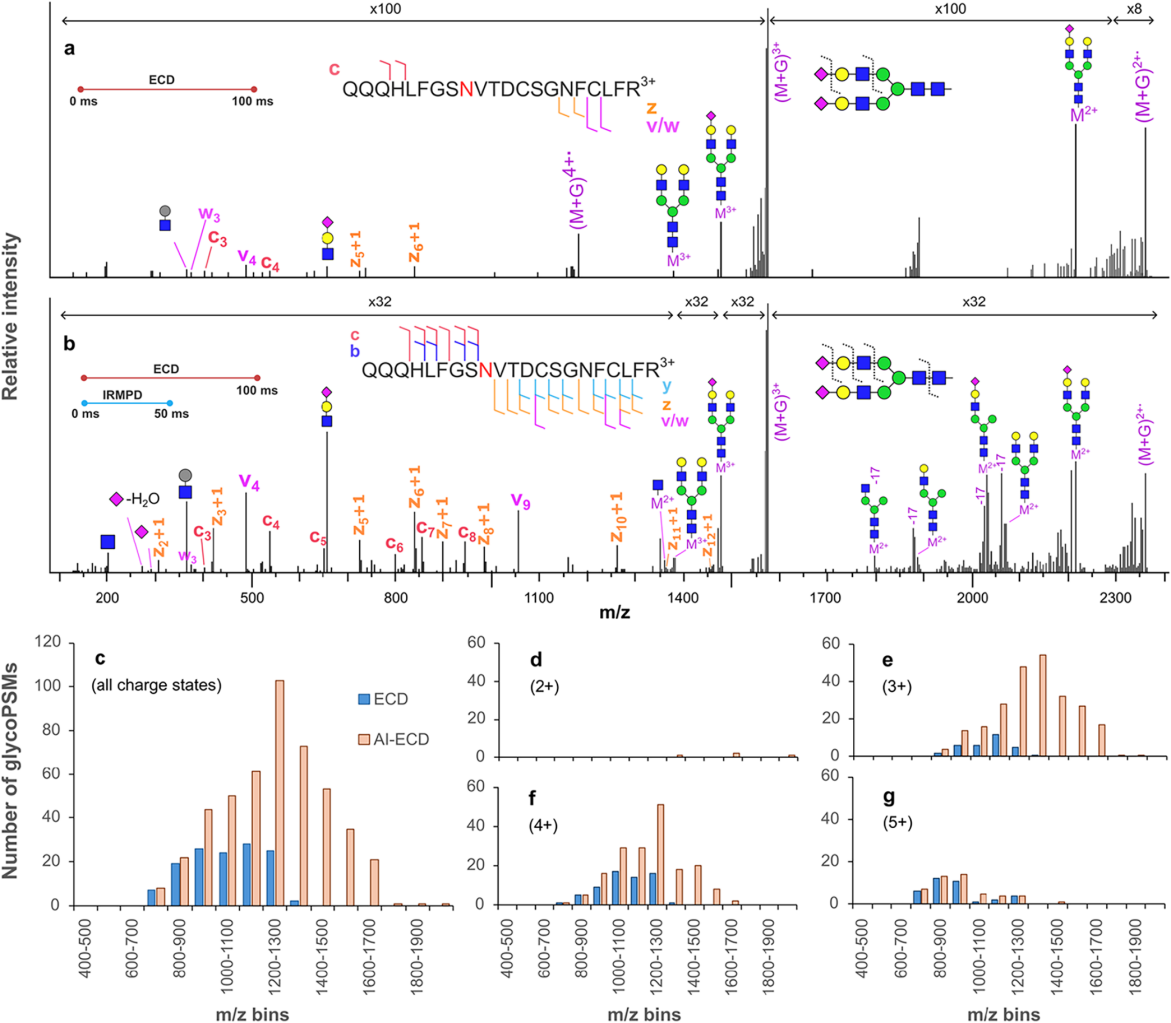
ECD (a) and AI-ECD (b) spectra of *N*-glycosylated QQQHLFGSNVTDCSGNFCLFR^3+^ acquired in LCMS of a complex glycopeptide mixture. For visual clarity, only *c* and *z* ions were annotated, full annotation can be found in Supporting Information Figure S15. All annotations of glycans correspond to *B* or *Y* fragments unless otherwise specified. Intact peptide is denoted as M, and precursor ions are annotated as (M+G). Precursor ions were irradiated by electrons for 100 ms. In AI-ECD, precursor ions were in addition coirradiated by IR light at 13% of the laser duty cycle for the first 50 ms of ECD. (c–g): M/z-binned distributions of the numbers of glycoPSMs identified in ECD (blue) and AI-ECD (orange) analyses of complex glycopeptide mixture: (c) all charge states, (d) doubly, (e) triply, (f) 4+, (g) 5+ charged precursors; *b,y,c,z* fragments were used for identification.

Next, we optimized the parameters of EID and UVPD in LCMS setting. We recorded the numbers of nonglycoPSMs and glycoPSMs while varying the number or energy of laser pulses in UVPD and irradiation time in EID and keeping other parameters fixed. For automated data analysis, we compared the following combinations of peptide fragments: *b,y*, as they were found to be essential for analysis of UVPD and EID of nonmodified peptides,^34^ *a,b,y*, as *a* ions are the third most frequent type of ions in UVPD and EID,^34^ *b,y,c,z* as default parameters in the “N-Hybrid” workflow in MSFragger, and we also included *a,b,y,c,z* set of fragments specifically in the analysis of EID data, as this combination showed the best results for nonmodified peptides.^34^

The analysis of the glycopeptide EID and UVPD data sets showed the presence of both nonmodified and *N*-glycosylated peptides (Figure 2). The identification rates of nonglycoPSMs at different UVPD and EID parameters (Figure 2a) were in line with our previous study.^34^ The identification of glycoPSMs, however, followed completely different patterns, especially in UVPD. The numbers of identified glycoPSMs across the range of UVPD and EID parameters depended on which peptide fragments are used for analysis (Figure 2b). The *b,y* pair of fragments showed clearer trends in UVPD with only one maximum and no sharp increases/drops in the range of energies and numbers of laser pulses, while simply adding *a* ions to the search parameters caused the appearance of one minimum and two maxima (Figure 2b). Furthermore, the optimal fragmentation in the LCMS UVPD analysis of glycopeptides was achieved at remarkably higher energies and pulses than those required to identify nonmodified peptides. In the analysis using *b,y* types of peptide fragments, two laser pulses at 6 mJ/pulse allowed to identify only 4 glycoPSMs, and this number gradually increased until the maximum of 555 glycoPSMs was reached at 8 laser pulses (Figure 2b). For nonglycoPSMs, this dependency flattened out after 4 laser pulses (Figure 2a), in agreement with our findings in the UVPD-LCMS of nonmodified peptides.^34^ In a range of pulse energies, the number of identified glycoPSMs started with 365 at 4 mJ/pulse and reached the maximum of 799 at 8 mJ/pulse. These observations are in line with the findings described for the UVPD of glycopeptides in the group of Brodbelt.^24,26^ As the search engine used in this work employs “peptide first” approach to identify glycopeptides, the identification of a precursor relies on the availability of peptide fragments in the first place. We assumed therefore that the efficiency of fragmentation of the peptide chain in a glycopeptide was significantly lower than in a nonmodified peptide. To explain this phenomenon in UVPD, we note that a 193 nm photon can be absorbed either by a backbone amide bond or aromatic residues within the peptide^48,49^ or by N-acetyl group in saccharides.^50^ The absorption of a photon by the latter may lead to an intensive loss of an acetyl moiety (Supporting Information Figures S5 and S7), cleavage of labile glycan bonds due to intramolecular vibrational redistribution, or cross-ring fragmentation via unknown mechanisms. Similar reasonings can be applied to the EID of glycopeptides, even though its initiation mechanism is fundamentally different. Depending on the location and kinetics of the interaction between an electron and a glycopeptide ion, the precursor may undergo dissociation at either peptide backbone, amino acid side chain, or anywhere within the glycan. We conclude that large glycans absorb some portion of irradiating photons or electrons and thus reduce the signal originating from peptide backbone fragmentation.

**Figure 2:**
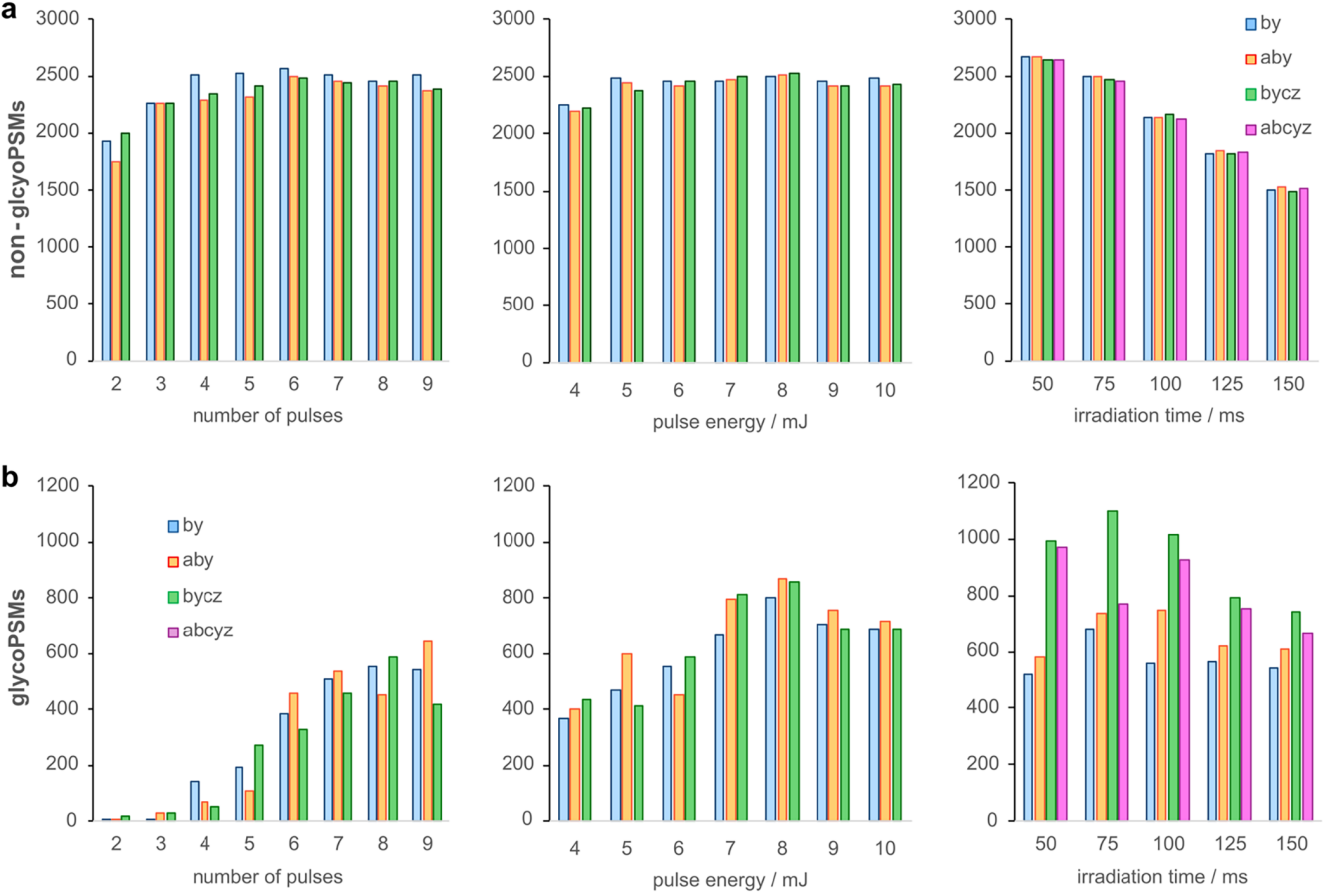
Numbers of identified nonglycoPSMs (a) and glycoPSMs (b) in UVPD in a range of numbers of laser pulses at 6 mJ/pulse (left) and pulse energies for 8 pulses (middle) and in EID data in a range of electron irradiation times (right).

In EID, the *b,y,c,z* combination showed noticeably better results in terms of identification numbers than *b,y* and *a,b,y* (Figure 2b). This was a somewhat unexpected observation, as no such difference between different combinations of peptide fragments was observed for nonglycoPSMs in the EID data and in the UVPD data on both glyco- and nonglycoPSM levels (Figure 2a,b). This implies that EID of glycopeptides produces on average more *c,z*-types of peptide fragments than UVPD. The number of nonglycoPSMs dropped as the irradiation time increased, potentially because of lower scan rates (Figure 2a). When using *b,y,c,z* peptide fragments for analysis, the number of glycoPSMs reached its maximum of 989 at 75 ms and then began to decline, also likely due to lower scan rate (Figure 2b).

### Depth of LCMS Analysis by ExD, UVPD, and sceHCD

We further compared the absolute numbers of identifications for each dissociation technique using optimal fragmentation parameters and MS1 *m*/*z* range of 900–2000 Th to minimize interference with nonglycosylated peptides. Comparing the final identification results (Figure 3a) allowed a number of observations to be made. SceHCD predictably produced the highest number of identifications, nearly twice as many as the second best, largely due to higher scan rate (Supporting Information Table S1). UVPD had the same efficiency per MS2 scan as sceHCD, 6.8 vs 6.7%, and EID fell behind with only 5.9% rate (Supporting Information Table S1). Further analysis revealed that HCD oversampled precursors with the same peptide and saccharide compositions more often compared to other techniques (Figure 3b), although it was not clear whether these oversampled precursors were entirely identical or represented isobaric glycan isomers. In terms of glycoforms per *N*-glycosylation site that differ in their saccharide composition, HCD showed the highest diversity reaching up to 43 glycoforms and the least proportion of glycosylation sites having only one, with EID being close second (Figure 3c). We attribute the richness of data provided by sceHCD to its superior scan rate, and we believe that the identification numbers and depth of analysis by the other techniques could be increased by implementing product-dependent (“pd”) approach, where ExD/UVPD events are triggered only following a detection of certain oxonium ions in a “scout” HCD scan (HCD-pd-ExD or HCD-pd-UVPD). ExD techniques would benefit the most, as they were nearly three times slower than sceHCD. Given that the rate of identification per MS2 spectra were similar across all techniques (Supporting Information Table S1), the total numbers of glycoPSMs in such HCD-triggered ExD or UVPD experiments may gain approximately 30%. One caveat in such estimations is the depth of glycopeptide enrichment, where lower proportions of glycosylated peptides among unmodified ones benefit the most from the product-dependent approach in terms of depth of analysis. For example, Sutherland et al. previously reported the comparison between sceHCD and sceHCD-pd-EThcD.^51^ Based on their data, we estimated that an EThcD spectrum had been on average four times slower than an sceHCD one, and the number of *N*-glycopeptides analyzed by sceHCD-pd-EThcD could have been two times greater than in EThcD alone. In our work, the scan rate of UVPD was on average half of that for sceHCD (Supporting Information Table S1); if one could replace nonglycoPSM UVPD scans by HCD and assuming the extra time would be spent on glycopeptide precursors, the number of additional glycoPSMs would be 893/2 = ∼446, which theoretically amounts to 446/1383 = 32% gain.

**Figure 3:**
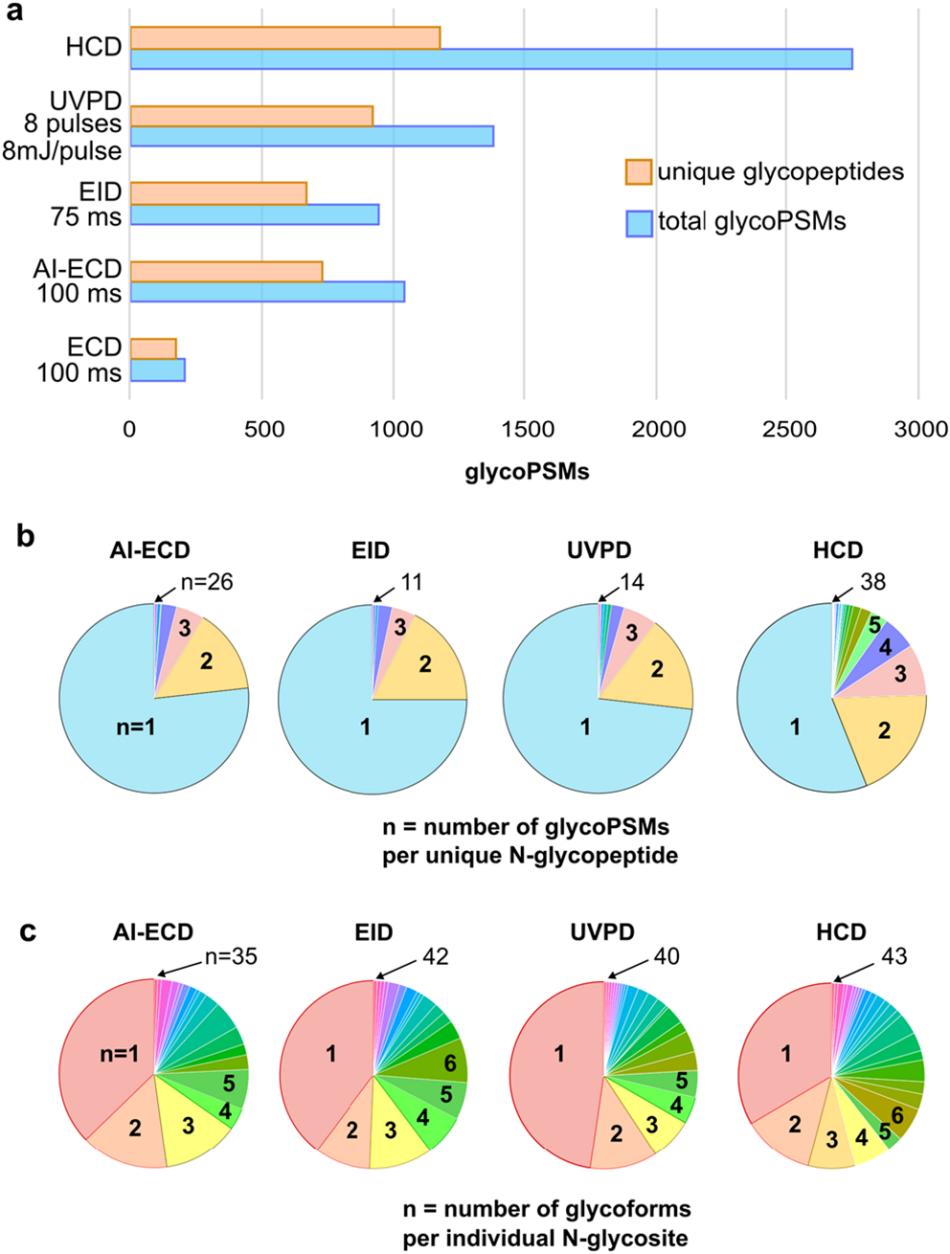
(a) Numbers of unique glycopeptides and glycoPSMs identified in a complex glycopeptide mixture by each fragmentation technique. In AI-ECD experiment, precursor ions were first coirradiated by electrons and IR light for 50 ms and then by electrons only for another 50 ms. (b) Pie charts showing the proportions of unique *N*-glycoPSMs (unique composition of peptide and glycan sequences) measured *n* times among all glycoPSMs. (c) Pie charts showing the proportions of individual *N*-glycosylation sites modified with *n* different glycoforms among all *N*-glycosylation sites.

ECD is a highly charge-dependent process favoring higher precursor-charge states and higher charge densities (Supporting Information Figure S16).^34^ In glycopeptide analysis, where charge densities are typically lower compared to bare peptides, the major products of ECD are often nondissociated charge-reduced ions, and the signal of *c*, *z*, oxonium, and *Y*-type fragments is poor, if at all detectable (e.g., Supporting Information Figure S3 and Figure 1a). This is not dissimilar to its sister technique, ETD, where additional activation in the form of collisions (ETciD or EThcD) or infrared light (AI-ETD) is required for obtaining sequencing signals and realizing the benefit of ETD.^13,25,52^ For automated analysis, oxonium ions are particularly important as most search engines rely on their presence in a spectrum to categorize it as a potential glycoPSM. ECD on its own becomes a very potent tool at higher charge states, being able to produce near-complete peptide sequence coverage by *c* and *z* ions retaining intact glycan and generate a few abundant oxonium and *Y*-type ions for 5+ precursors as exemplified in Supporting Information Figure S17. This makes it a compelling choice for highly charged precursors during LCMS acquisition, where EID and UVPD often perform worse due to signal splitting across multiple fragment charge states and highly congested spectra (Supporting Information Figure S16).^34^ ECD furthermore no longer requires supplemental IR activation for 5+ precursors (Figures 1g and S16), although such IR activation boosted the overall numbers of glycoPSMs identified in AI-ECD experiments more than five times compared to ECD alone (sum of all charge states where 3+ dominate) which was sufficient to provide parity to EID but not UVPD (Figure 3a). The efficiency of IRMPD also depends on the charge state and *m*/*z* (which are proxy for size) of a glycopeptide precursor.^21^ While insufficient activation results in too few oxonium and glycosidic fragments, too much IRMPD may lead to adverse fragmentation of *c,z* fragments generated by ECD. Both these outcomes are undesirable, and finding a sweet spot to enable data-dependent approach can be challenging but highly beneficial for ECD and AI-ECD of glycopeptides. Reassuringly, the charge-dependent performances of AI-ECD, EID, and UVPD were very similar to sceHCD (Supporting Information Figure S16), which implies no loss of information or precursor-charge bias among these techniques compared to the conventional collisional approach.

We then compared the level of information provided by UVPD, EID, and sceHCD LCMS spectra of a typical glycopeptide. The results can be exemplified by the triply charged peptide AALAAFNAQNNGSNFQLEEISR modified with a complex *N*-glycan [HexNAc4Hex5NeuAc2] for which we have annotated corresponding spectra (Figure 4). These spectra were acquired in the experiments where optimal parameters of fragmentation were used, namely, 8 pulses at 8 mJ/pulse for UVPD and 75 ms of irradiation time for EID. Noteworthy, the amount of information we observed in this UVPD and EID LCMS spectra was on par with direct-infusion experiments of glycopeptide standards (Supporting Information Figures S5–S8) which suggests that this instrument design is suitable for detailed structural characterization of glycopeptides on the LCMS scale. As expected, stepped collisional fragmentation generated a complete peptide sequence coverage by (deglycosylated) *b,y* ions and dissociated all glycosidic bonds within the glycan. UVPD and EID also confirmed the peptide identification by providing an abundance of main-series peptide fragments. We did not find any peptide fragments containing an intact glycan, and we only observed one deglycosylated *b*-ion in EID. Assuming the glycan composition given in Figure 4d, all three spectra contain cross-ring and glycan internal fragments (Supporting Information Figure S18), which are potentially useful in that they may allow one to determine types of glycosidic bonds. Despite their conceivably high utility for search engines, one first needs to perform their annotation to evaluate frequency, consistency, and prominence. Unfortunately, no such automated method currently exists, which requires the annotation to be done manually for each spectrum, the task which is practically impossible on large data sets and without prior knowledge of glycan structures. Furthermore, many fragment ions have multiple isobaric matches which further complicates their annotation, and the question persists whether one should consider annotations arising from two cross-ring cleavage events (Supporting Information Figure S9). In view of this, we were not able to confirm or discard the assumed composition of a glycan in Figure 4. We envisage though that mining these types of fragments may become one of the future directions of research and development of software suites for detailed glycopeptide analysis and *de novo* glycan sequencing. Coupled with advanced MS acquisition strategies such as charge- and *m*/*z*-dependent decision trees and product-dependent ExD/UVPD triggering, such in-depth MS characterization of glycoforms can significantly improve structural analysis of protein glycosylation.

**Figure 4:**
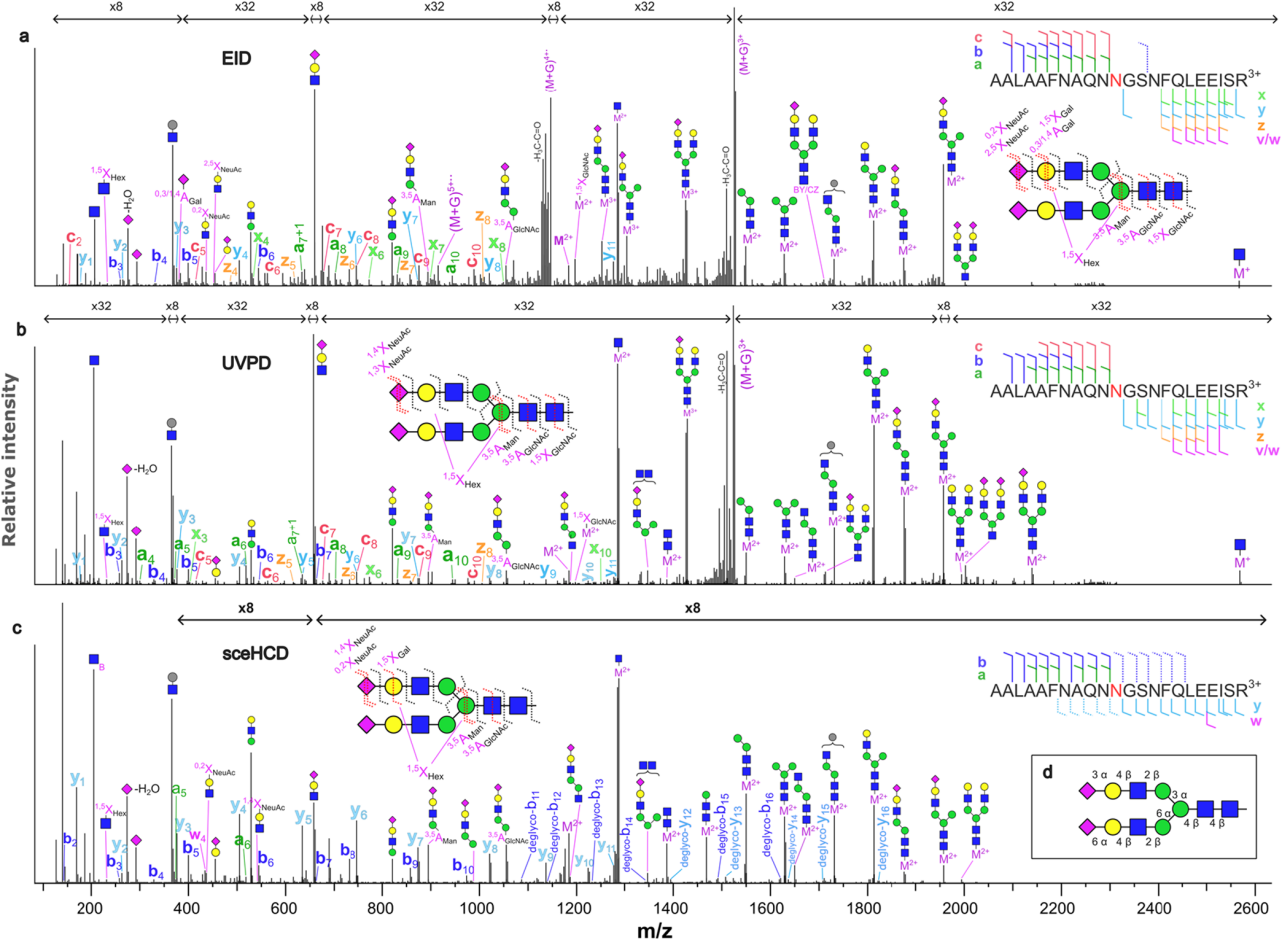
EID (a), UVPD (b), and sceHCD (c) spectra of *N*-glycosylated AALAAFNAQNNGSNFQLEEISR^3+^ peptide acquired in the LCMS analysis of a complex glycopeptide mixture. For visual clarity, only the most abundant ions were annotated, full annotation can be found in Supporting Information Figure S18. Intact peptide is denoted as M, and precursor ions are labeled as (M+G). All annotations of glycans correspond to *B* or *Y* fragments unless otherwise specified. Dashed labels correspond to fully deglycosylated peptide fragments. For UVPD, 8 pulses at 8 mJ/pulse were used; in EID, precursor ions were irradiated by 25 eV electrons for 75 ms. Assumed composition of the glycan is shown in panel (d).

### Ion Statistics in ExD, UVPD, and sceHCD LCMS Analyses

Next, we assessed how ExD and UVPD perform in the identification of amino acid and glycan parts of glycopeptides using MSFragger. For this purpose, we plotted the distributions of two types of scores generated by MSFragger—Glycan score and hyperscore—against each other for glycoPSMs acquired at optimal parameters of sceHCD, UVPD, and EID (Supporting Information Figure S19). We also plotted the distributions of hyperscores for glyco- and nonglycoPSMs against precursor *m*/*z* identified in the same experiments (Supporting Information Figure S20). Glycan score reflects the abundance of *Y*-type glycan fragments and oxonium ions,^39^ and hyperscore correlates with the number of peptide fragments.^36^ In sceHCD, the distribution of Glycan scores is by design centered at zero, with higher (positive) values indicating higher number of matched glycan-related peaks.^36^ Our sceHCD data were heavily biased toward higher Glycan Scores and has a long tail of IDs with low values of both scores (Supporting Information Figure S19a). We were pleased to see that UVPD and EID in our experiments produced very similar distributions with regards to Glycan score (Supporting Information Figure S19), which suggests sufficient fragmentation of glycosidic bonds to form abundant oxonium and *Y*-ions in these two processes. The hyperscores of glycoPSMs, on the other hand, were drastically different in sceHCD, where they reached the values of around 75, and in UVPD and EID, where they largely fell below 30 (Supporting Information Figure S20a,b) when *b,y* peptide fragments were used for data analysis. Note that while including additional ion series such as *c*, *z*, *a*, or *x* for UVPD and EID would increase hyperscores, the additional permutations (and the presence of glycan containing fragments) will lead to higher numbers of false positives that can result in fewer glycoPSMs reported at a given FDR (Figure 2). Further characterization and better predictive powers will help control FDR while better spectral annotation, similar to what has been demonstrated for unmodified peptides.^34^ Collisional dissociation is known to produce partially and fully deglycosylated peptide fragments^53,54^ that contribute to the hyperscore in sceHCD. Relatively low hyperscores in UVPD and EID suggest that these two processes generate fewer identifiable peptide fragments, in particular C-terminal from the glycosylation site for *y* types of fragments, and N-terminal—for *b* ions as observed in Figure 4. To test this hypothesis, we explored peptide fragmentation statistics in more detail (Supporting Information Figure S21). The number of dissociated bonds within those parts of peptide sequences that were free from glycan by main-series fragments were in line with earlier observation for nonmodified peptides.^34^ In particular, *y* ions were the most frequent among all types of fragments in UVPD, EID, and sceHCD, and EID exhibited more *a*, *x*, *c*, *z* ions than UVPD (Supporting Information Figure S21a), supporting our earlier search-based observation that EID produces more *c,z* types of peptide fragments (Figure 2b, see also Supporting Information Figure 20c,d). Beyond the glycosylation site, the peptide sequence coverage by either C- and N-terminal deglycosylated ions was very poor in AI-ECD, UVPD, and EID and reached 20–30% at best, with *b* ions showing the best results (Supporting Information Figure S21b). In contrast, sceHCD showed on average better coverage by deglycosylated *b* and *y* ions reaching as much as 100% sequence coverage in some cases by each type of ions (Supporting Information Figure S21b). The retention of an *N*-glycan was negligibly low in all fragmentation techniques (Supporting Information Figure S21c).

More detailed analysis of ion-type statistics revealed slightly different proportions of peptide, oxonium, and *Y*-types of fragment ions across the dissociation techniques (Figure 5). EID, UVPD, and sceHCD were overall similar to each other, with oxonium being by far the most abundant type of fragment (Figure 5a). EID showed the highest proportions of peptide–ion intensities, and sceHCD had the lowest, which can be linked to different varieties and abundances of fragments generated in these processes. The ratio between median intensities of oxonium and *Y*-type was the lowest in AI-ECD, which also had remarkably large spreads of intensities within each group of ions. This potentially arose from the inability to tune the laser power to charge and *m*/*z* of precursors resulting in either too low oxonium or excessive secondary dissociation. AI-ECD also exhibited a skewed distribution of the number of distinct types of oxonium ions per spectrum, peaking at six, whereas UVPD, EID, and sceHCD had a maximum at eight (Figure 5b). Relative intensities of a few common oxonium fragments showed similar trends for all techniques (Figure 5c), although sceHCD had less sialylated GlcNAcGalNeuAc (*m*/*z* 657) and slightly more abundant GlcNAc (*m*/*z* 204). The latter can be attributed to more favorable low-mass cutoff parameters in sceHCD experiments (125 *m*/*z* vs 150 *m*/*z* in other techniques). Binned histograms of mass accuracies (in ppm) for oxonium-, peptide-, and *Y*-type fragment ions followed normal distributions (Supporting Information Figure S22). The distributions of the three types of ions appear to correlate with size, oxonium distributions were narrower due to their small size as opposed to peptide- and *Y*-type ions whose masses are much larger and dependent on peptide sequence and glycan composition.

**Figure 5:**
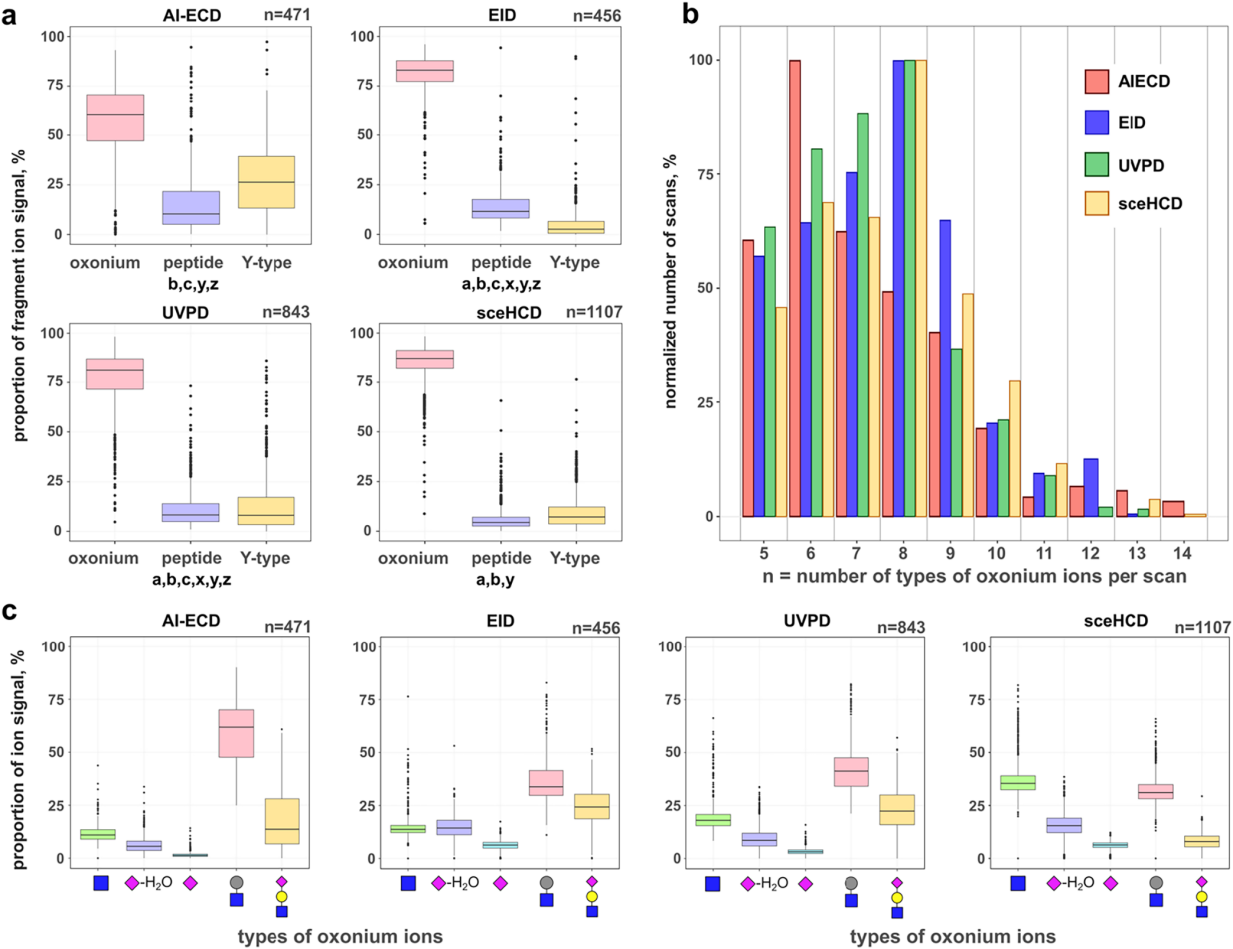
(a) Boxplots of intensities of oxonium-, peptide-, and *Y*-type fragment ions in AI-ECD, EID, UVPD, and sceHCD normalized to the highest intensity within these groups in each spectrum. Sample sizes are given as *n* numbers above the panels; (b) number of scans containing *n* distinct types of oxonium ions in AI-ECD, EID, UVPD, and sceHCD normalized to the maximum number of scans for each technique; (c) boxplots of intensities of five types of oxonium ions normalized to total oxonium signal in each spectrum. GlycoPSMs duplicates were filtered to keep the one with the highest hyperscore.

### Characterization of UVPD and ExD of O-Glycopeptides

As discussed above, the increase in energy required to fragment a glycopeptide over a nonmodified peptide suggests that the size of the glycan may influence the extent of fragmentation. The ability of UVPD to comprehensively sequence small *O*-glycopeptides and localize *O*-glycosylation sites was demonstrated conclusively by Brodbelt and co-workers.^24,27^ We hypothesized that in our experiments too, a peptide modified with a relatively small glycan had a higher chance of being retained upon EID or UVPD than a larger glycan. Although the enrichment method was not designed to capture *O*-glycopeptides, we still were able to find up to 162 *O*-glycoPSMs in our sample (Supporting Information Tables S2–S5). Figure 6 shows EID, UVPD, and sceHCD LCMS spectra of a triply charged 21-amino acid long *O*-glycopeptide TEHLASSSEDSTTPSAQTQEK with nine potential sites of *O*-glycosylation. SceHCD provided an excellent peptide and glycan sequence coverage by *b*, *y*, and *Y* fragments; it, however, exhibited substantial loss of the sugar leaving an intact amino acid behind which precludes site localization. The only two peptide fragments retaining a part of the glycan were b_13_ and b_14_ ions bearing a GalNAc fragment, which narrows down the list of possible glycosylation sites to seven amino acids (Figure 6c). UVPD and EID spectra contain a rich array of peptide main-series fragments in addition to a few products of glycosidic bond and cross-ring dissociation. Similar to Figure 4, we were not able to confirm the exact isoform of the glycan on the basis of the observed fragments. Importantly though, y_9_^2+^, b_13_^2+^, and a range of *a*-type fragments allow us to localize the *O*-glycan to the Thr13 residue (Figure 6a,b). In addition, the presence of the fragment d_13_^2+^ (see Supporting Information Figure S14 for definition) in both EID and UVPD might possibly serve as indirect evidence of the presence of a modification at Thr13. AI-ECD of the same glycopeptide produced a nearly complete peptide sequence coverage by *c* and *z* ions retaining the glycan (Supporting Information Figure S23).

**Figure 6:**
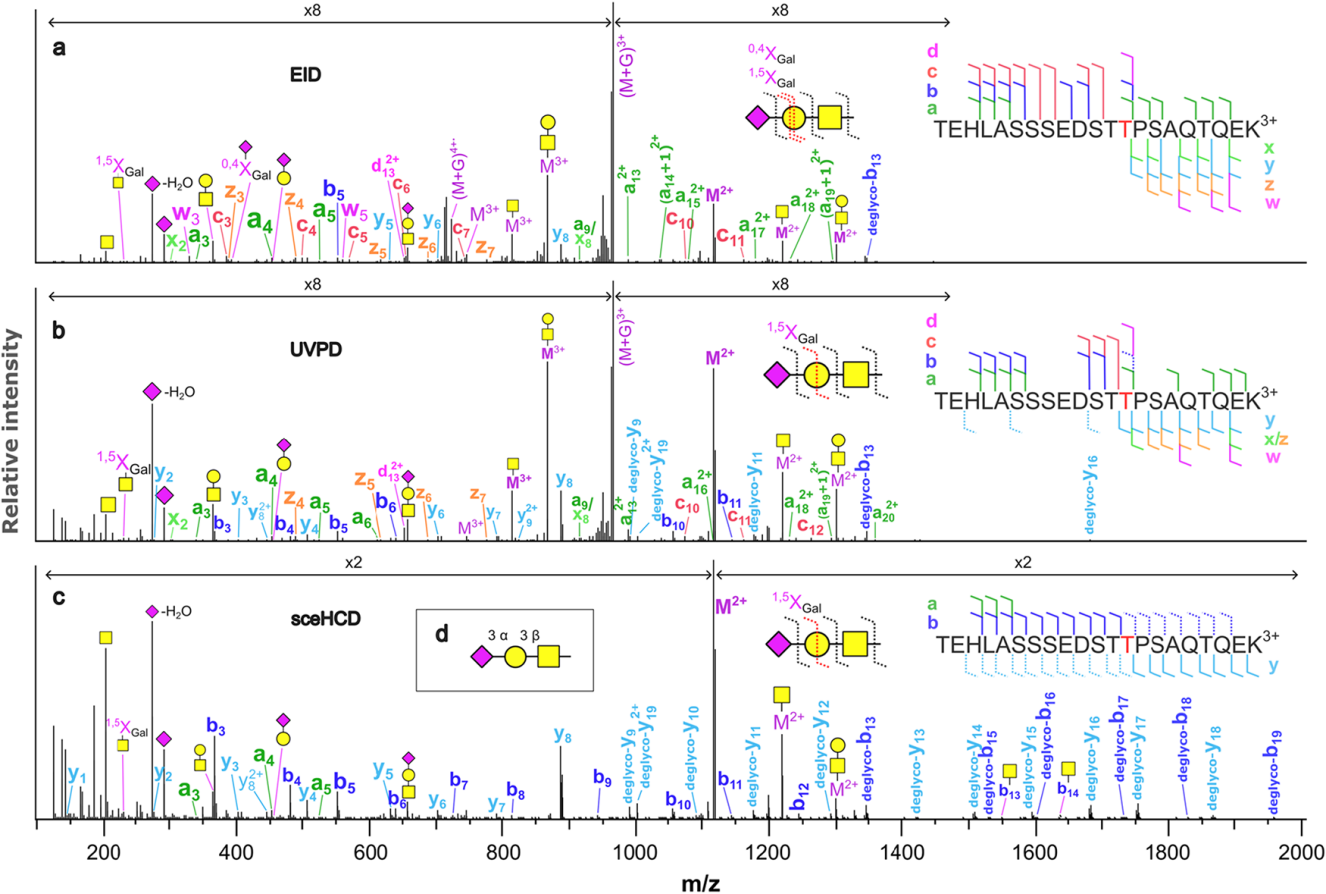
UVPD (a), EID (b), and sceHCD (c) spectra of *O*-glycosylated TEHLASSSEDSTTPSAQTQEK^3+^ acquired in the LCMS analysis of a complex glycopeptide mixture. For visual clarity, only the most abundant ions were annotated, full annotation can be found in Supporting Information Figure S24. Intact peptide is denoted as M, and precursor ions are annotated as (M+G). All annotations of glycans correspond to *B* or *Y* fragments unless otherwise specified. Dashed labels correspond to fully deglycosylated peptide fragments. For UVPD, 8 pulses at 8 mJ/pulse were used; in EID, precursor ions were irradiated by 25 eV electrons for 100 ms. Assumed composition of the glycan is shown in (d).

## Conclusions

This study establishes Omnitrap as a potent technology that allows for acquisition of high-quality detailed UVPD and EID spectra of complex *N*-glycopeptides in both direct-infusion and LCMS modes. Through systematic evaluation of UVPD and EID parameters, we showed that these alternative activation modes provide enhanced fragmentation of peptide backbones and glycan moieties of glycopeptides beyond what collisional methods can achieve, but at the same time highlight the need for development of optimized acquisition strategies for optimal sequencing of both peptide and glycan parts of the analyte. We furthermore showed that ECD alone suffers from low dissociation yields, which can be rectified by supplemental activation by IR light. Finally, we demonstrated that the scoring patterns produced by a search engine for EID and UVPD glycopeptide data are substantially different compared to collisional data, which suggests further software development may be required for efficient analysis of such types of data. In current N-glycopeptide workflows, sceHCD remains the preferred routine method for high-throughput glycopeptide identification and glycan-composition assignment. The practical value of UVPD and ExD lies instead in targeted or product-dependent experiments, where additional peptide backbone, glycosidic, internal, and cross-ring fragments are needed to interrogate spectra that are compositionally ambiguous, potentially isomeric, or structurally undetermined by collisional dissociation alone.

## Supplementary Material



## Data Availability

Raw LCMS data have been uploaded to the ProteomeXchange Consortium via the PRIDE partner repository with the data set identifiers PXD071023.

## Notes

Glycan nomenclatures; compositions of standard glycopeptides; GlyCounter search parameters; additional direct-infusion and LCMS spectra of glycopeptides; ion statistics in direct-infusion and LCMS experiments; nonglycoPSMs and glycoPMSs identification statistics; definitions of d-, v-, and w-ions; fully annotated Figures 1, 4, and 6; glycan scores vs hyperscores; hyperscores *vs m*/*z* (PDF)
