# Supplementary material for: Evaluating the Performance of Photon- and Electron-Based Fragmentation Methods in Omnitrap-LCMS Analysis of N‑Glycopeptides
Source: Anal Chem. 2026 Jun 26;98(27):20043–54. doi: 10.1021/acs.analchem.6c00435 (PMC13373915; doi:10.1021/acs.analchem.6c00435)
Supplement: Supplementary file 1 [file ac6c00435_si_001.pdf]

Supporting Information

Evaluating the performance of photon- and electron-based fragmentation methods in Omnitrap-LCMS analysis of N-glycopeptides

Nikita Levin[1,2*], Daniel A. Polasky[3], Kai Li[4], Alexey I. Nesvizhskii[3,4], Shabaz Mohammed[1,5,6*]

[1] Rosalind Franklin Institute, Harwell Campus, OX11 0QX Didcot, United Kingdom

[2] Department of Pharmacology, University of Oxford, OX1 3QT Oxford, United Kingdom

[3] Department of Pathology, University of Michigan, Ann Arbor, Michigan 48109, United States

[4] Gilbert S. Omenn Department of Computational Medicine and Bioinformatics, University of Michigan, Ann Arbor, Michigan 48109, United States

[5] Department of Biochemistry, University of Oxford, OX1 3QU Oxford, United Kingdom

[6] Department of Chemistry, University of Oxford, OX1 3TA Oxford, United Kingdom

* Email: shabaz.mohammed@chem.ox.ac.uk and nikita.levin@bioch.ox.ac.uk

Table of contents

		Page number:
**Supplementary Figure S1**	Glycopeptide nomenclature, fragment nomenclature, compositions of glycopeptide standards	S3
**Supplementary Figure S2**	GlyCounter parameters	S4
**Supplementary Figure S3**	Direct-infusion ECD of double and triply charged A2G2S2 glycoform of a standard glycopeptide	S5
**Supplementary Figure S4**	Direct-infusion AI-ECD and ECD of A2G2S2 glycoform of a standard glycopeptide	S6
**Supplementary Figure S5**	Direct-infusion UVPD of two glycoforms (A2G2S2 and FA2) of a standard glycopeptide	S7
**Supplementary Figure S6**	Direct-infusion EID of two glycoforms (A2G2S2 and FA2) of a standard glycopeptide	S8
**Supplementary Figure S7**	Direct-infusion UVPD of double and triply charged A2G2S2 glycoform of a standard glycopeptide	S9
**Supplementary Figure S8**	Direct-infusion EID of double and triply charged A2G2S2 glycoform of a standard glycopeptide	S10
**Supplementary Figure S9**	Potential ambiguity in assignment of cross-ring glycan fragments	S11
**Supplementary Figure S10**	Absolute intensities of selected glycopeptide fragments under different UVPD parameters (doubly charged A2G2S2 glycoform)	S12
**Supplementary Figure S11**	Absolute intensities of selected glycopeptide fragments under different UVPD parameters (triply charged A2G2S2 glycoform)	S13
**Supplementary Figure S12**	Absolute intensities of selected glycopeptide fragments under different EID irradiation times (triply charged A2G2S2 and doubly charged FA2 glycoforms)	S14
**Supplementary Figure S13**	M/z-binned distributions of the numbers of non-glycoPSMs and glycoPMSs in sceHCD analysis	S15
**Supplementary Figure S14**	Definitions of *d*-, *v*- and *w*-ions	S16
**Supplementary Figure S15**	Complete annotations of ECD and AI-ECD spectra of the N-glycopeptide from Figure 1	S17
**Supplementary Figure S16**	Hyperscores and *m/z*-binned counts of glycoPSMs separated by charge state of precursors	S18
**Supplementary Figure S17**	ECD spectrum of a 5+ precursor of an N-glycopeptide acquired in ECD LCMS experiment	S19
**Supplementary Figure S18**	Complete annotations of EID, UVPD and sceHCD spectra of the N-glycopeptide from Figure 4	S20
**Supplementary Figure S19**	Glycan scores plotted against hyperscores for glycoPSMs identified in UVPD and EID under different experimental parameters	S21
**Supplementary Figure S20**	Hyperscores plotted against *m/z* of glycoPSMs and non-glycoPSMs identified in UVPD and EID under different experimental parameters	S22
**Supplementary Figure S21**	Binned coverages of peptide sequences by main-series ions in UVPD, ExD and sceHCD	S23
**Supplementary Figure S22**	Mass accuracies of oxonium-, peptide- and Y-type 1+ and 2+ fragment ions in AI-ECD, EID, UVPD and sceHCD	S24
**Supplementary Figure S23**	AI-ECD spectrum of AALAAFNAQ**N**NGSNFQLEEISR^{3+} glycopeptide from Figure 4	S25
**Supplementary Figure S24**	Complete annotations of EID, UVPD and sceHCD spectra of the O-glycopeptide from Figure 6	S26
**Supplementary Table S1**	Numbers of non-glycoPSMs and glycoPSMs identified under optimal experimental parameters	S27
**Supplementary Table S2-S5**	Lists of O-glycoPSMs identified in LCMS AI-ECD, EID, UVPD and sceHCD experiments	S28-S33

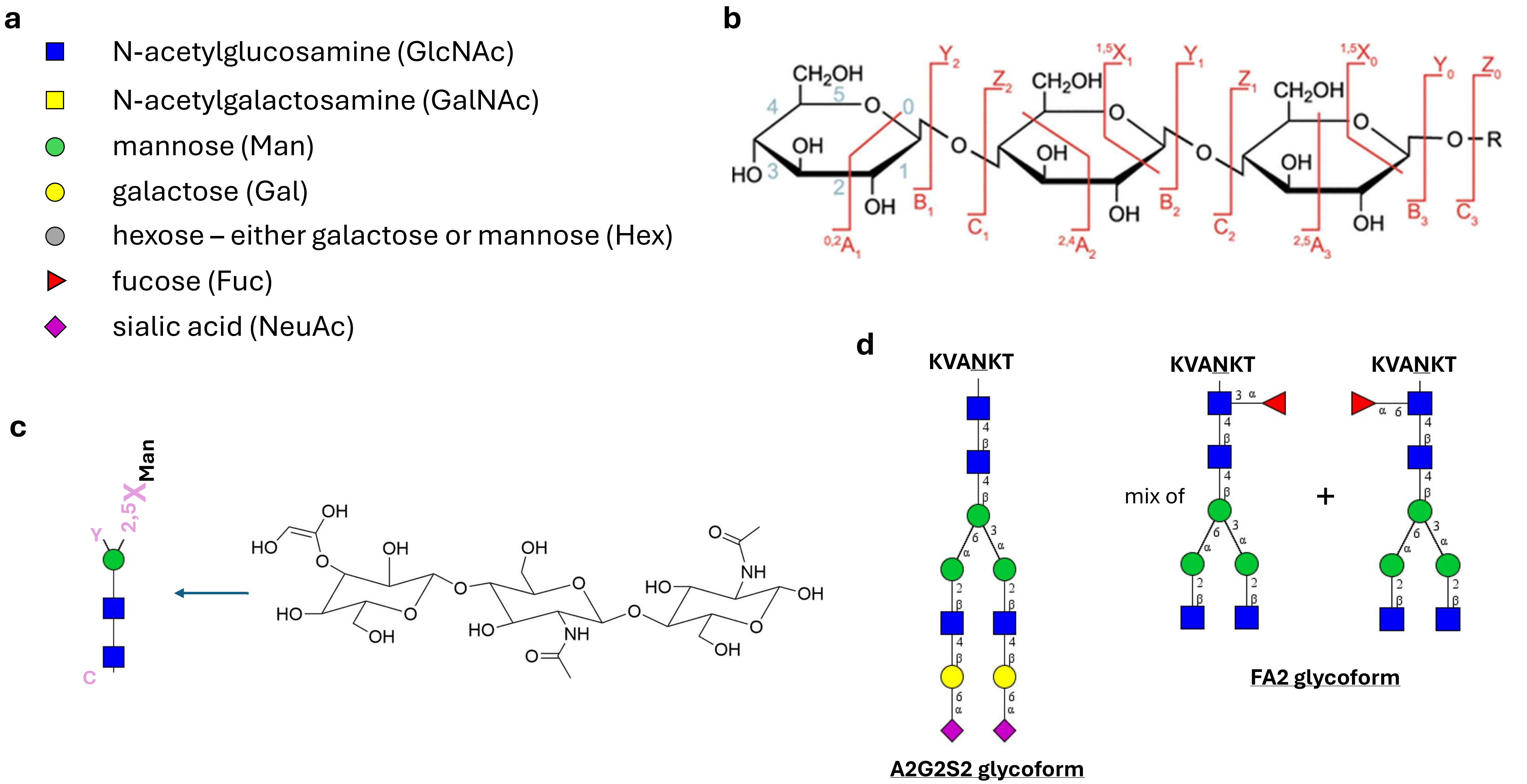

Supplementary Figure S1. **a)** Monosaccharide symbol nomenclature; **b)** glycan fragmentation nomenclature as proposed by Domon and Costello [Domon, Costello *Glycoconjugate J*., 5 (1988), p. 397], figure adopted from [Grabarics, Pagel et al., *Chem*. *Rev*. 2022, 122, 8, 7840-7908]; **c)** example of annotation of an internal glycan fragment used throughout this paper; **d)** compositions of two standard glycopeptides used in this work in direct infusion experiments.

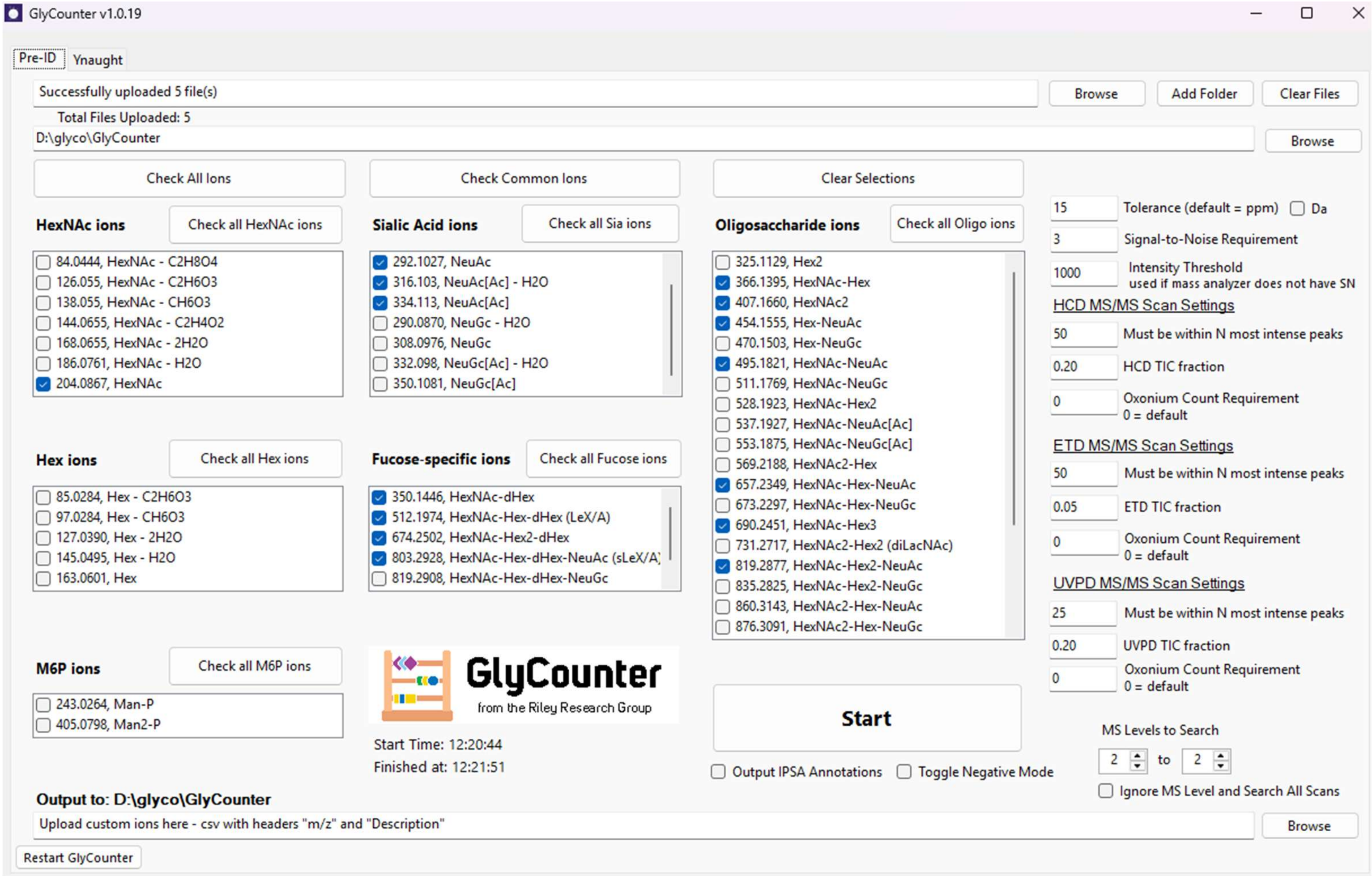

Supplementary Figure S2. GlyCounter parameters used for extracting glycan fragment ions.

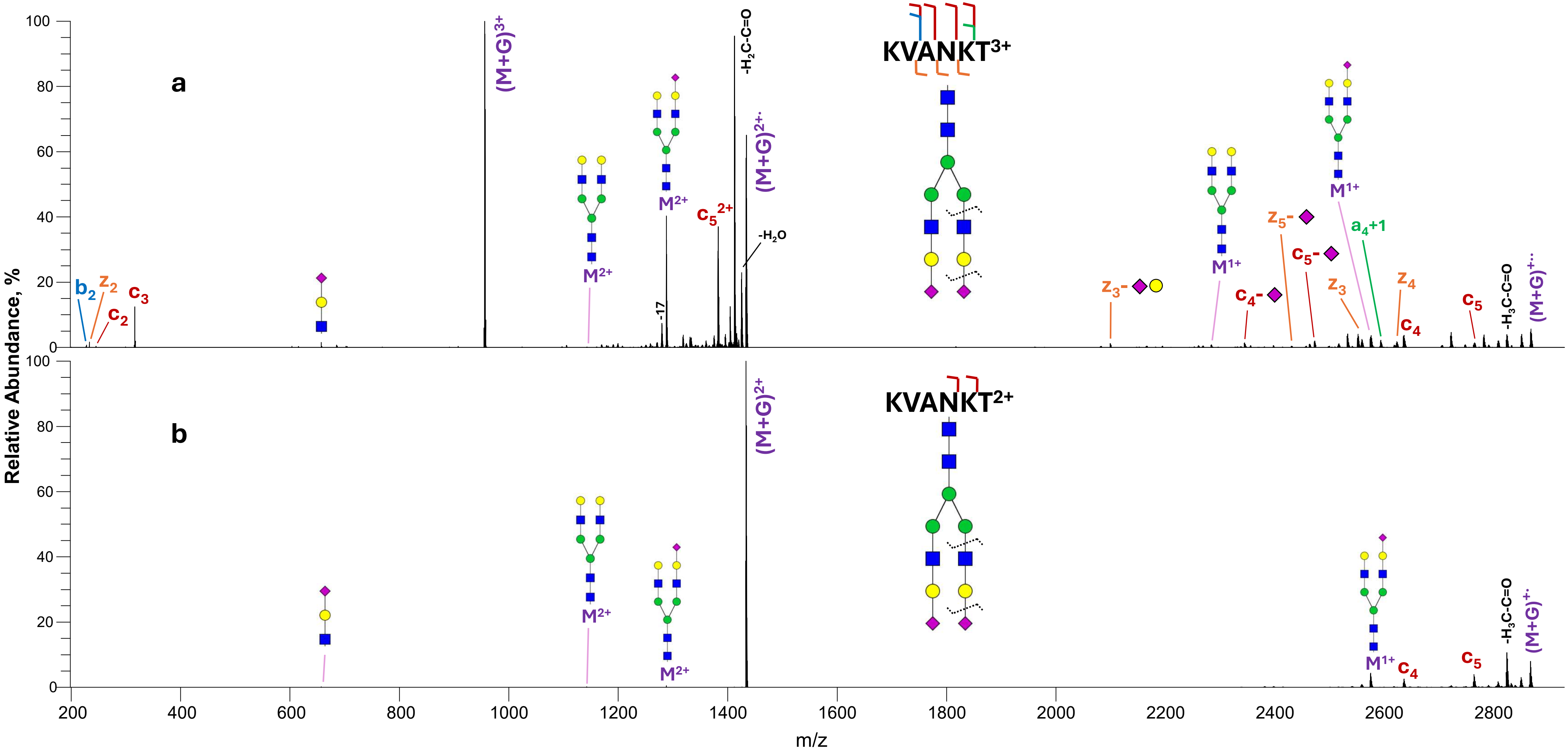

Supplementary Figure S3. Averaged MS2 ECD mass spectra of triply (**a**) and doubly (**b**) charged A2G2S2 glycoform of the standard glycopeptide, acquired following 150 ms of irradiation by 0-2 eV electrons. Intact peptide is marked with M, and precursor ions are annotated as (M+G). All annotations of glycans correspond to *B* or *Y* fragments.

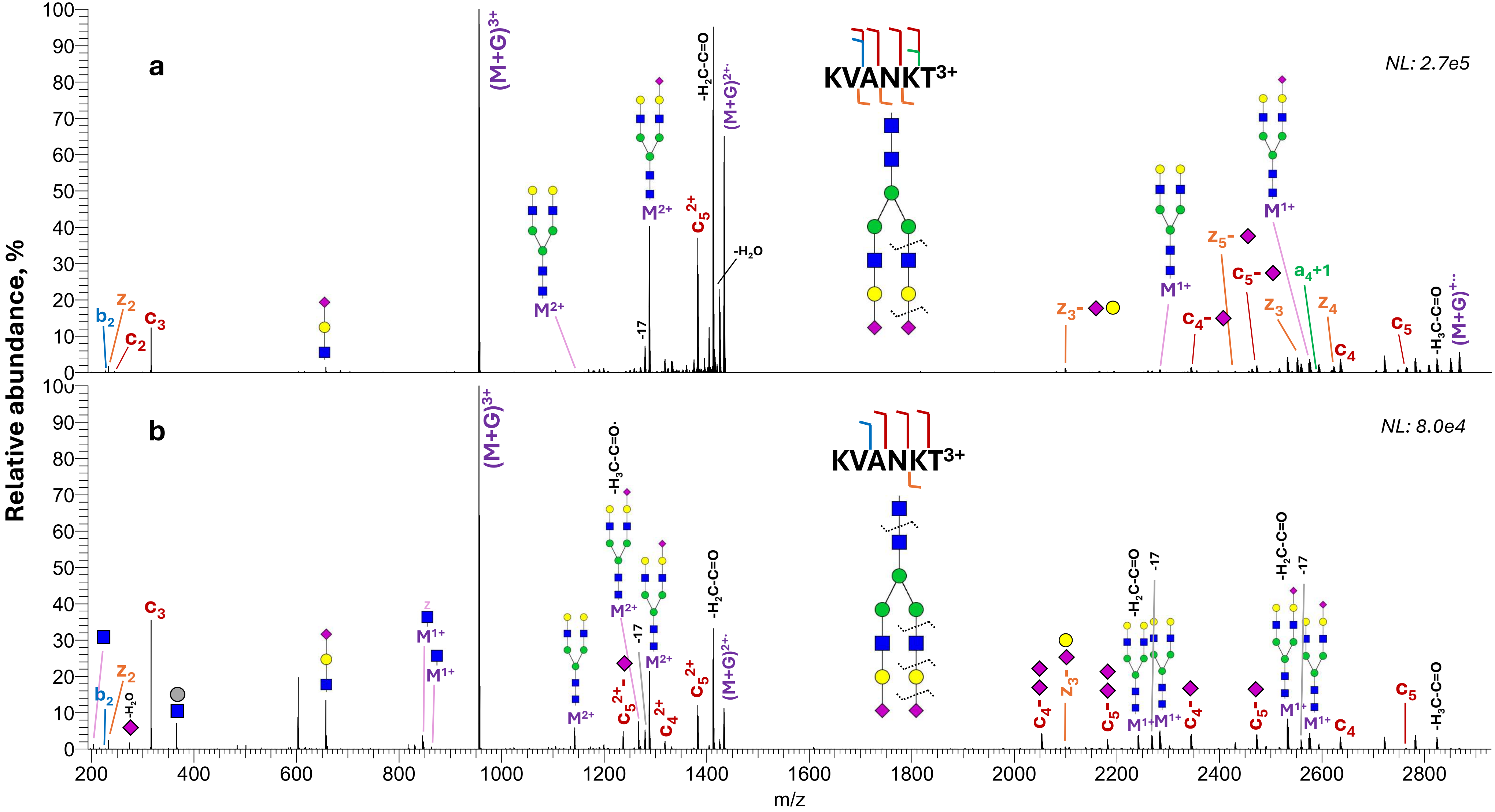

Supplementary Figure S4. Averaged MS2 ECD **(a)** and AI-ECD **(b)** mass spectra of triply charged A2G2S2 glycoform of the standard glycopeptide, acquired following 150 ms of irradiation by 0-2 eV electrons. In AI-ECD, precursor ions were in addition co-irradiated by IR light at 15 % of the laser duty cycle for the first 50 ms of ECD. Intact peptide is marked with M, and precursor ions are annotated as (M+G). All annotations of glycans correspond to *B* or *Y* fragments unless specified otherwise.

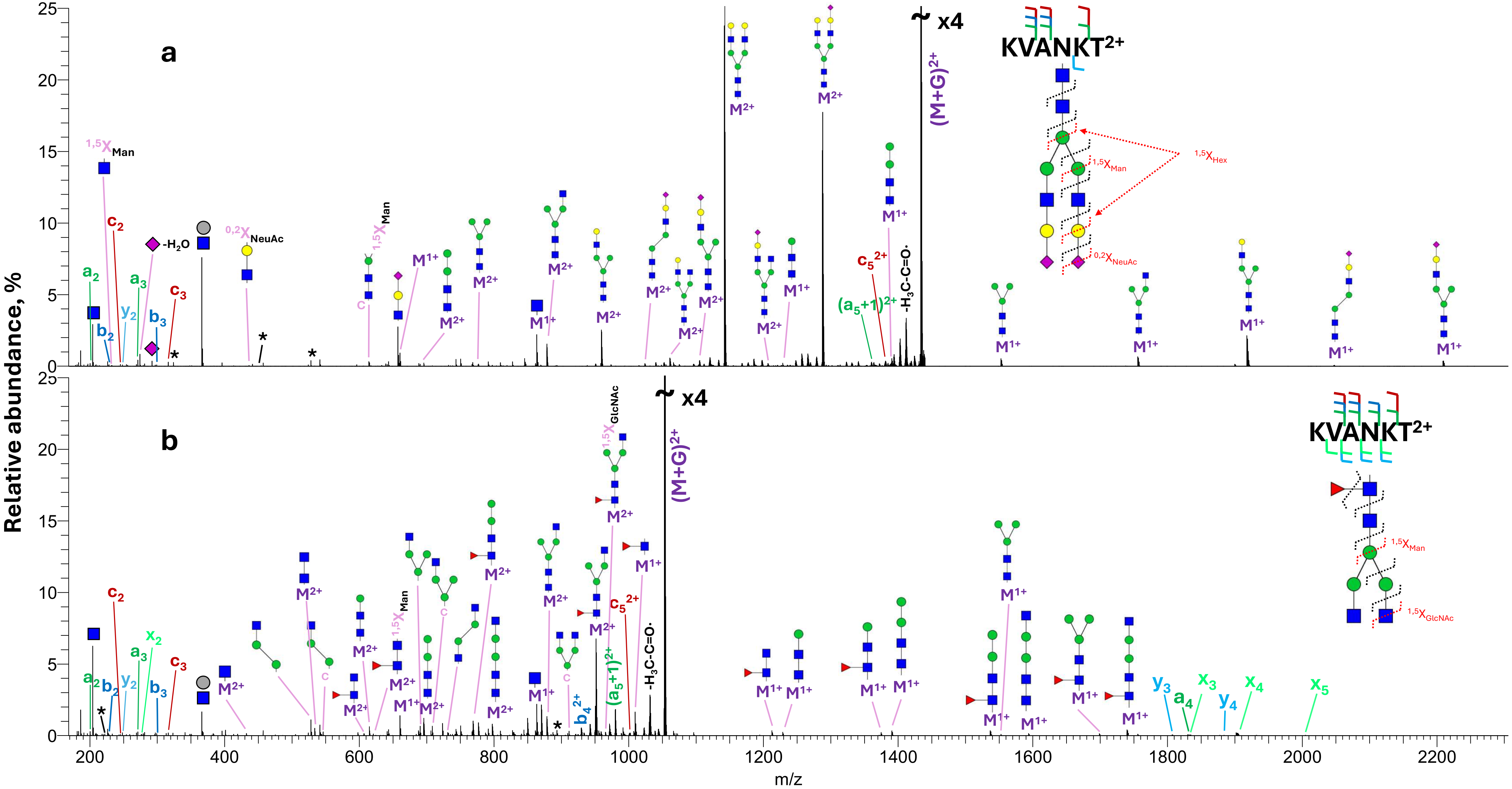

Supplementary Figure S5. Averaged MS2 UVPD mass spectra of doubly charged A2G2S2 (**a**) and FA2 (**b**) glycoforms of the standard glycopeptide, acquired using 10 laser pulses and 10 mJ/pulse. M/z values matching to multiple isobaric internal glycan fragmentation products are marked with asterisks. Intact peptide is denoted as M, and precursor ions are annotated as (M+G). All annotations of glycans correspond to *B* or *Y* fragments unless otherwise specified. Fragments matching neutral losses of water and –CO were not annotated.

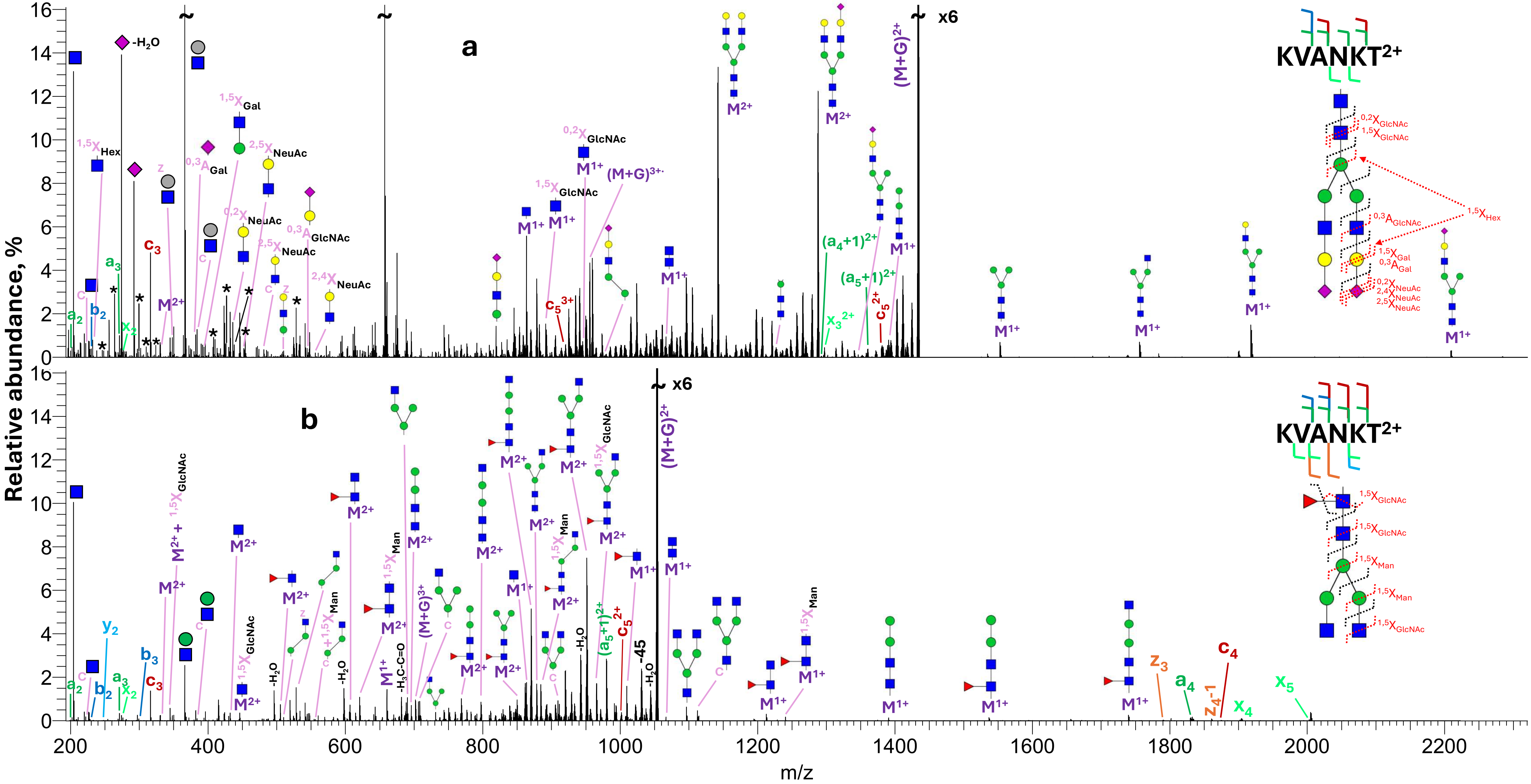

Supplementary Figure S6. Averaged MS2 EID mass spectra of doubly charged A2G2S2 (**a**) and FA2 (**b**) glycoforms of the standard glycopeptide, acquired after irradiating the precursor ions for 150 ms by 25 eV electrons. M/z values matching to multiple isobaric internal glycan fragmentation products are marked with asterisks. Intact peptide is denoted as M, and precursor ions are annotated as (M+G). All annotations of glycans correspond to *B* or *Y* fragments unless otherwise specified. Fragments matching neutral losses of water were not labelled. Typically, only one charge state of a fragment was annotated.

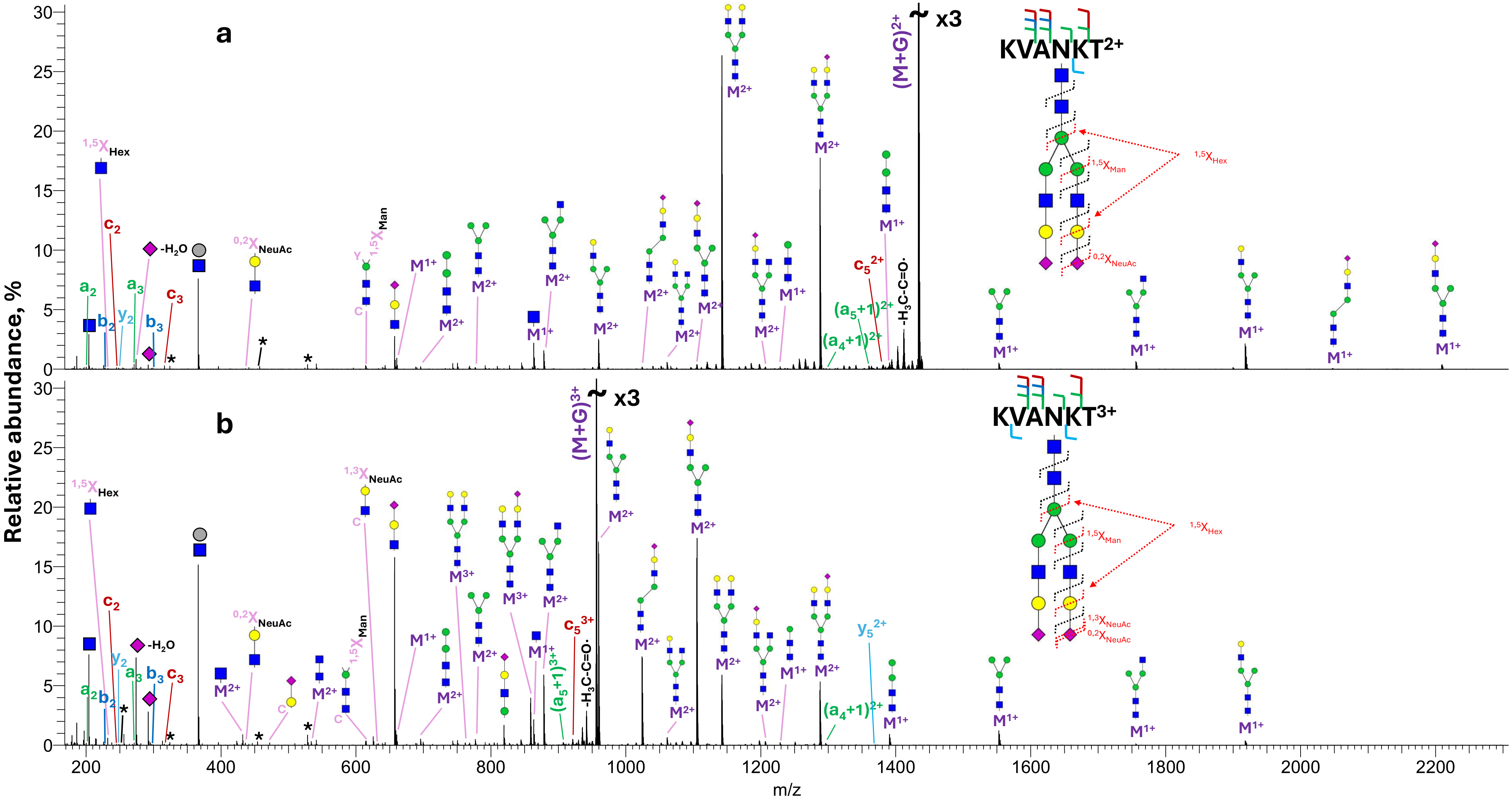

Supplementary Figure S7. Averaged MS2 UVPD mass spectra of doubly (**a**) and triply (**b**) charged A2G2S2 glycoform of the standard glycopeptide, acquired using 10 laser pulses and 10 mJ/pulse. M/z values matching to multiple isobaric internal glycan fragmentation products are marked with asterisks. Intact peptide is denoted as M, and precursor ions are annotated as (M+G). All annotations of glycans correspond to *B* or *Y* fragments unless otherwise specified. Fragments matching neutral losses of water and –CO were not annotated.

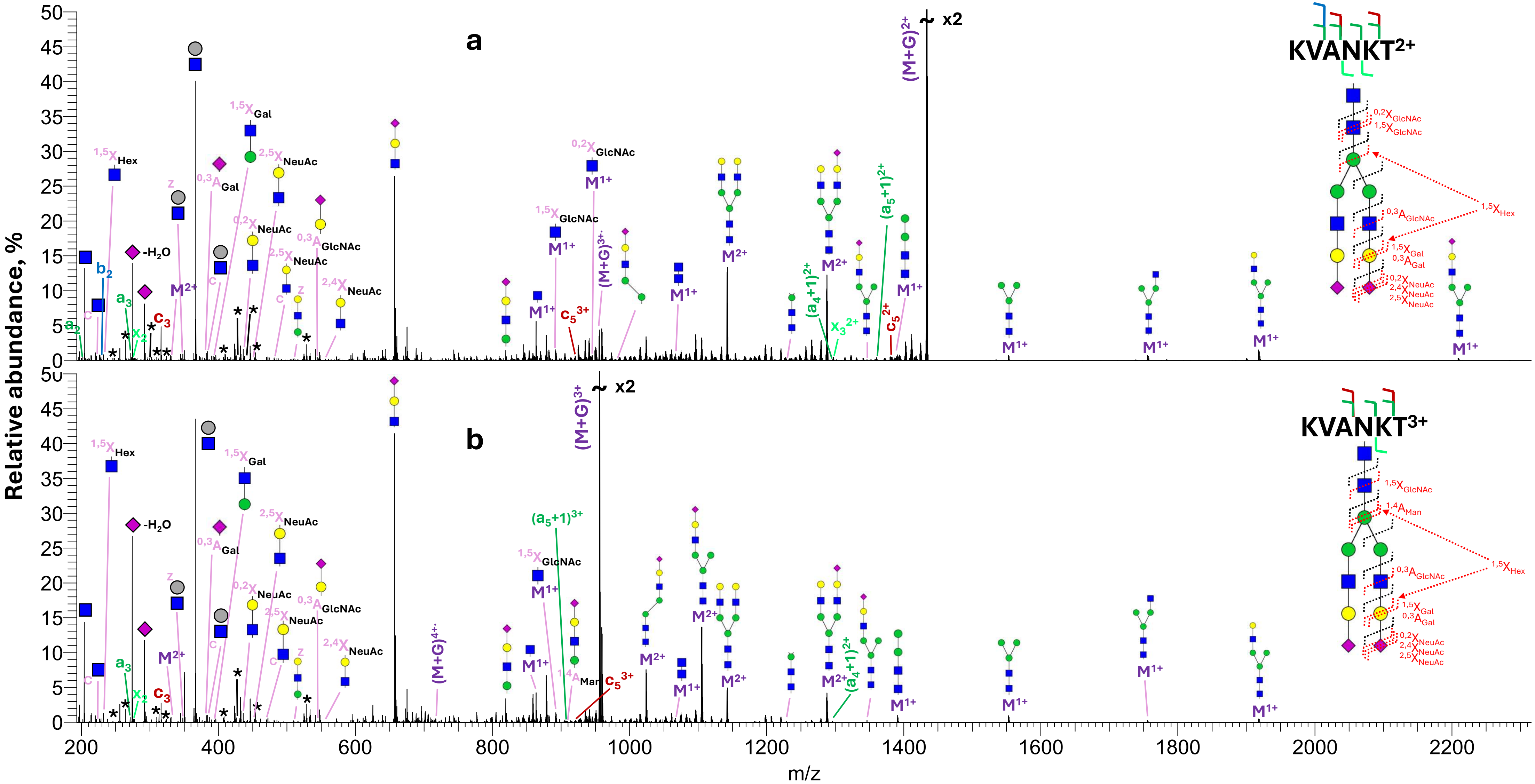

Supplementary Figure S8. Averaged MS2 EID mass spectra of doubly (**a**) and triply (**b**) charged A2G2S2 glycoform of the standard glycopeptide, acquired after irradiating the precursor ions for 150 ms (**a**) or 100 ms (**b**) by 25 eV electrons. M/z values matching to multiple isobaric internal glycan fragmentation products are marked with asterisks. Intact peptide is denoted as M, and precursor ions are annotated as (M+G). All annotations of glycans correspond to *B* or *Y* fragments unless otherwise specified. Fragments matching neutral losses of water were not labelled. Typically, only one charge state of a fragment was annotated.

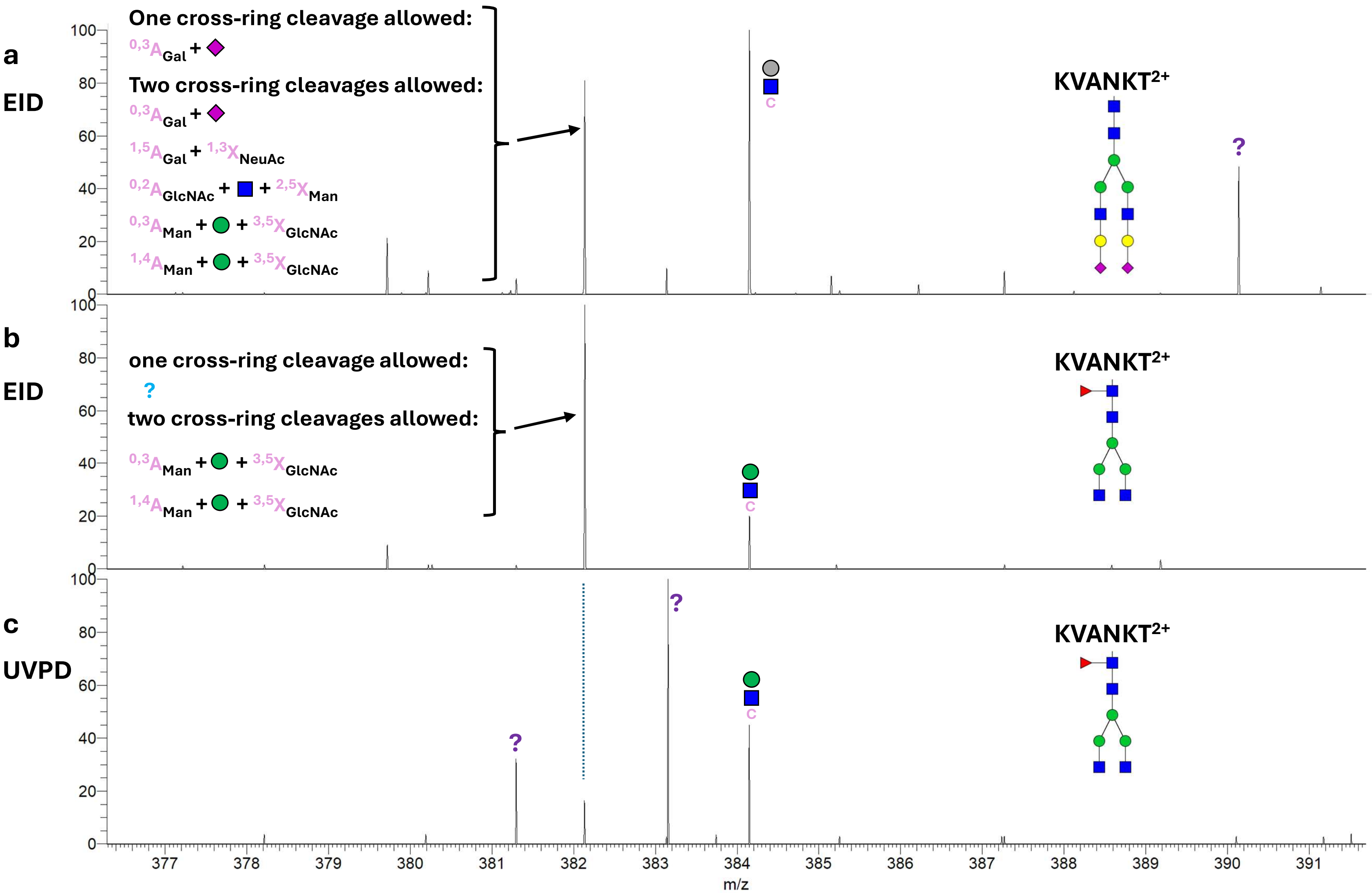

Supplementary Figure S9. Zoomed-in parts of mass spectra shown in Figures S6A **(a)**, S6B **(b)**, and S4B **(c)**, covering the m/z range between 377 and 391 Th. The fragment at m/z 382.134 may correspond either to $^{0,3}A_{Gal}$ fragment if only one cross-ring dissociation event is allowed or to multiple isobaric structures if two cross-ring dissociation events are allowed. All assignments are within 3 ppm mass accuracy.

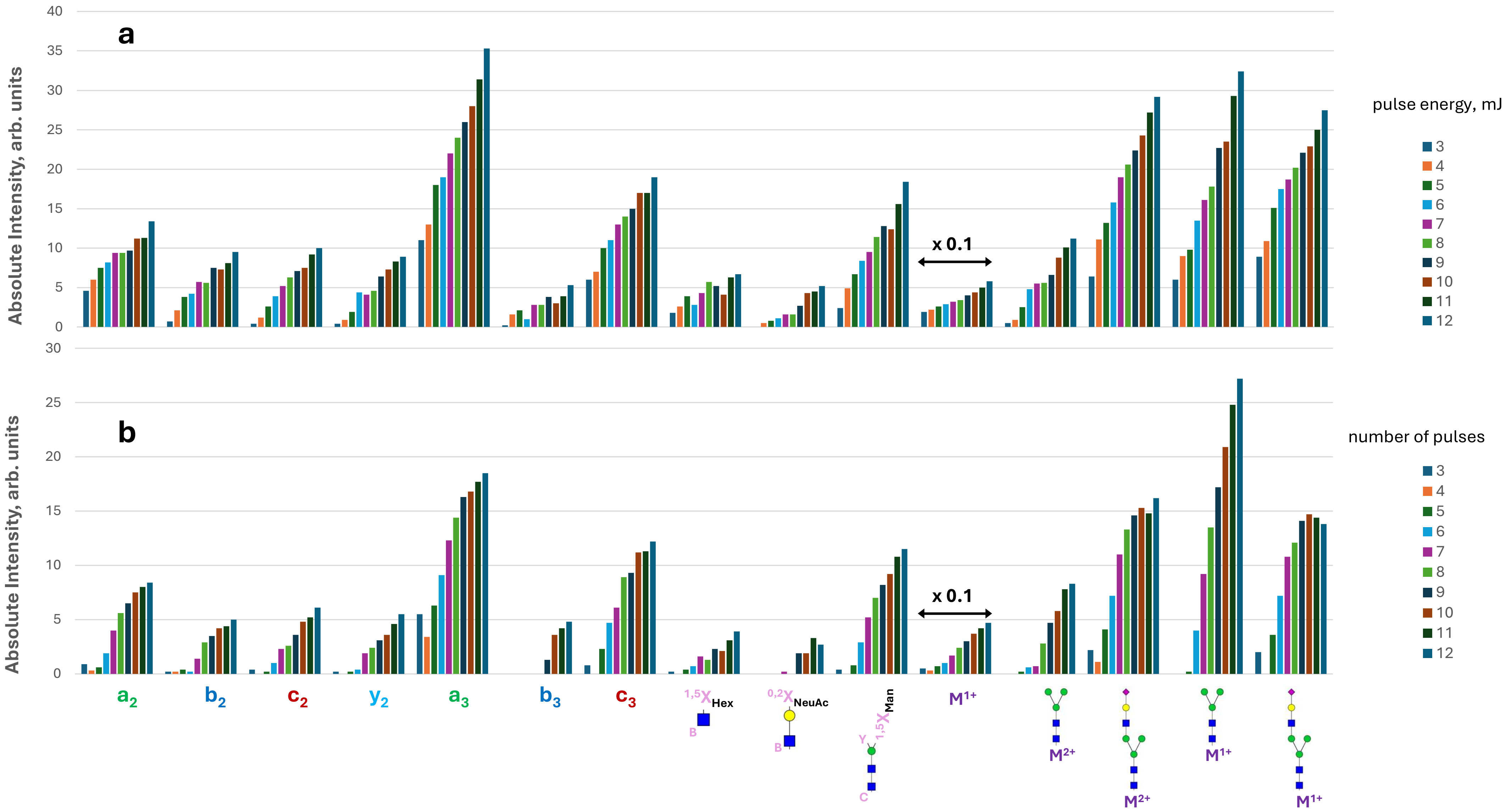

Supplementary Figure S10. Intensities of few selected fragments observed in averaged spectra acquired in direct infusion UVPD of doubly charged A2G2S2 glycoform of the standard glycopeptide using 8 laser pulses and different pulse energies (**a**) and different number of laser pulses at 10 mJ/pulse (**b**).

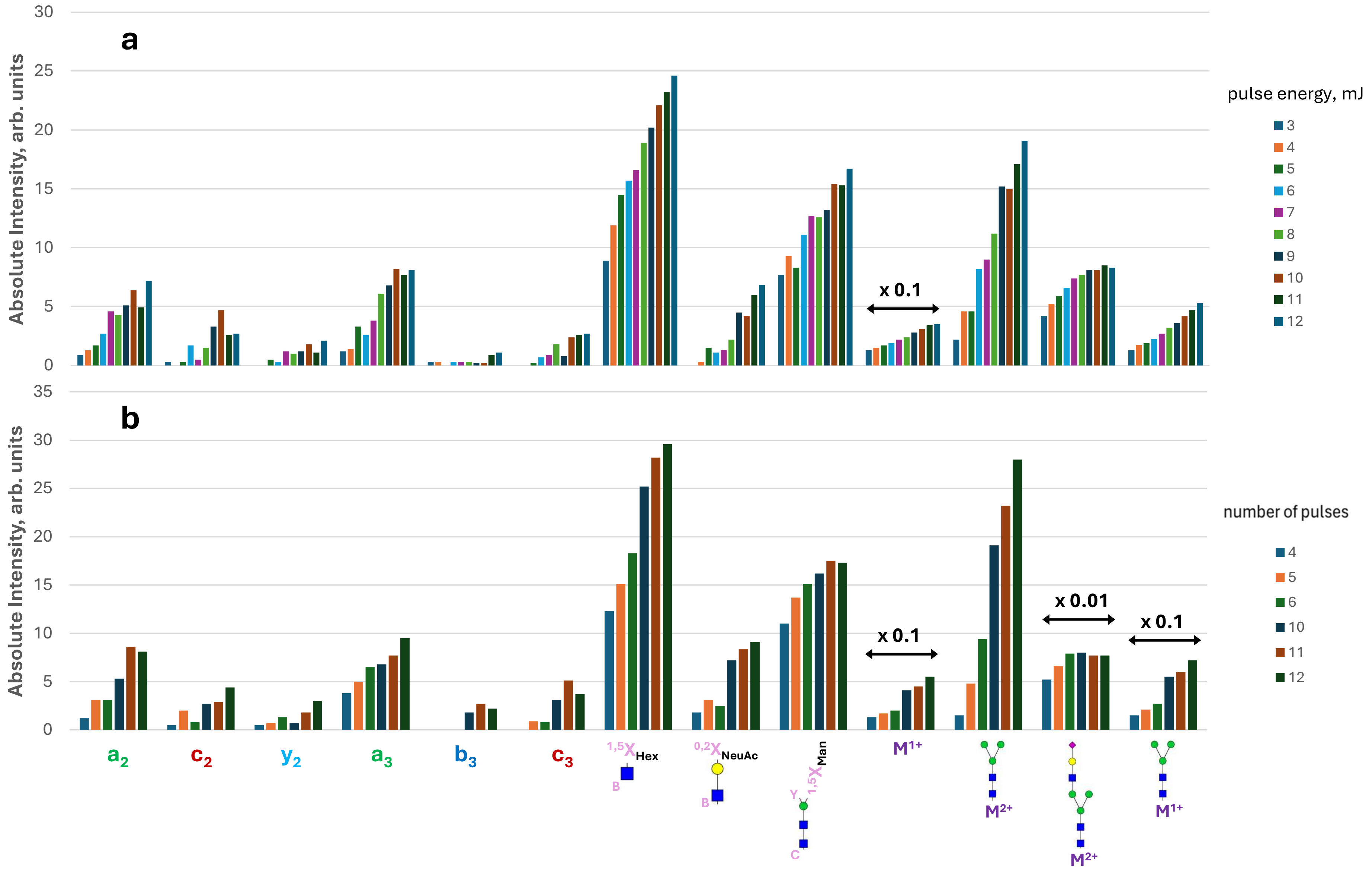

Supplementary Figure S11. Intensities of few selected fragments observed in averaged spectra acquired in direct infusion UVPD of triply charged A2G2S2 glycoform of the standard glycopeptide using 8 laser pulses and different pulse energies (**a**) and different number of laser pulses at 10 mJ/pulse (**b**).

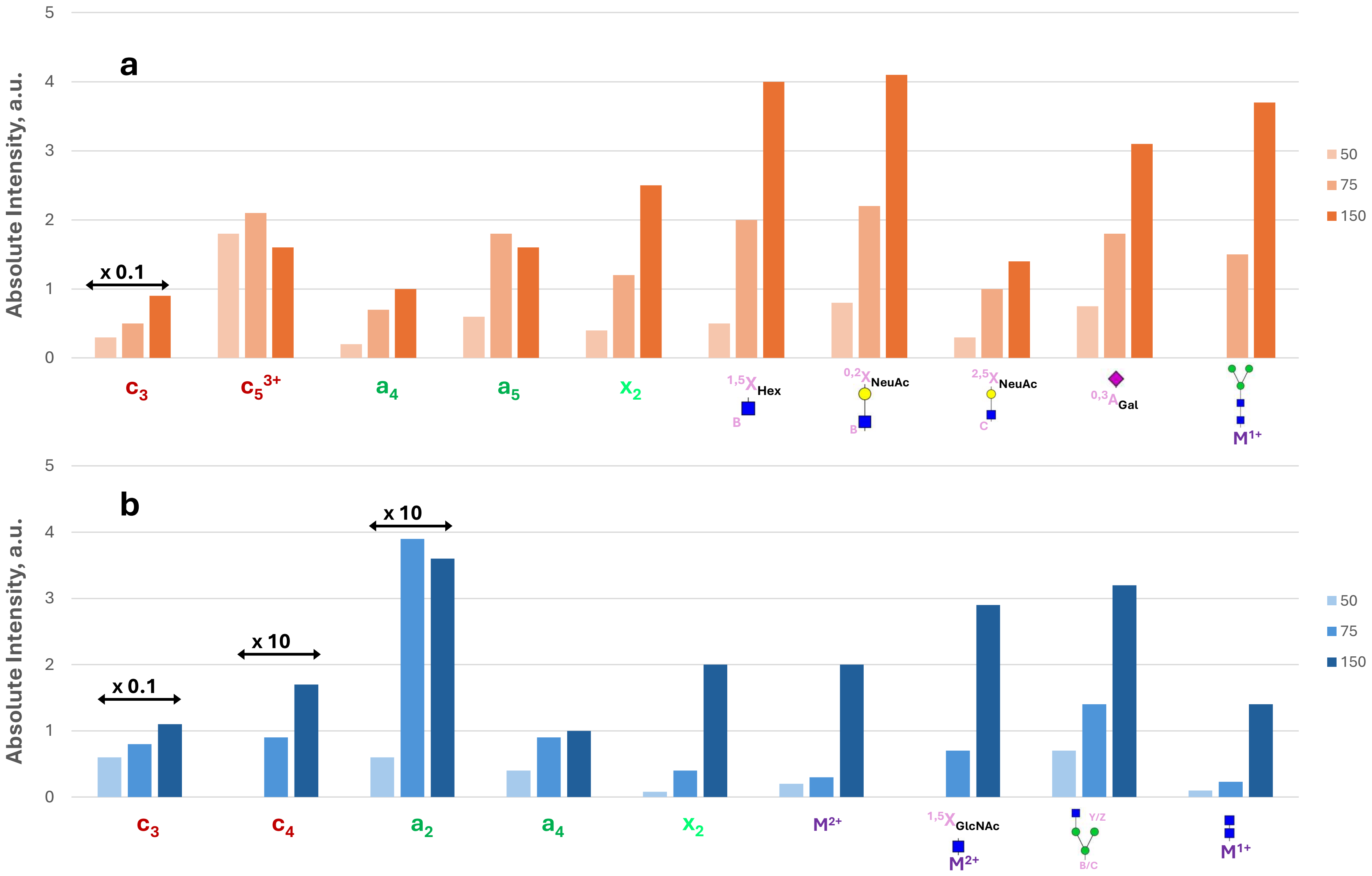

Supplementary Figure S12. Intensities of few selected fragments observed in averaged spectra acquired in direct infusion EID of triply charged A2G2S2 **(a)** and doubly charged FA2 **(b)** glycoforms of the standard glycopeptide using different irradiation times.

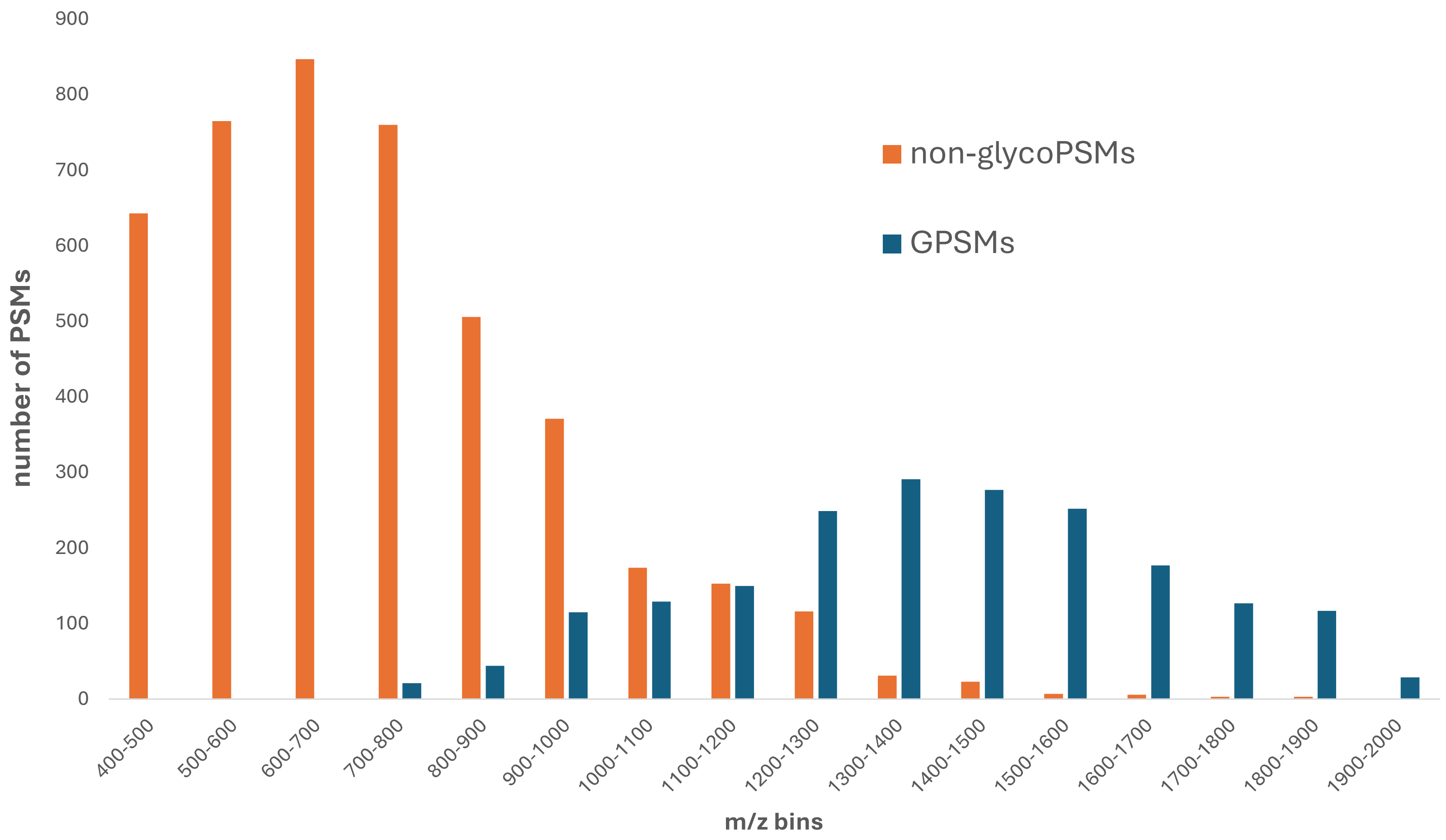

Supplementary Figure S13. M/z-binned distributions of the numbers of non-glycoPSMs (orange) and glycoPMSs (blue) identified in sceHCD analysis of complex glycopeptide mixture.

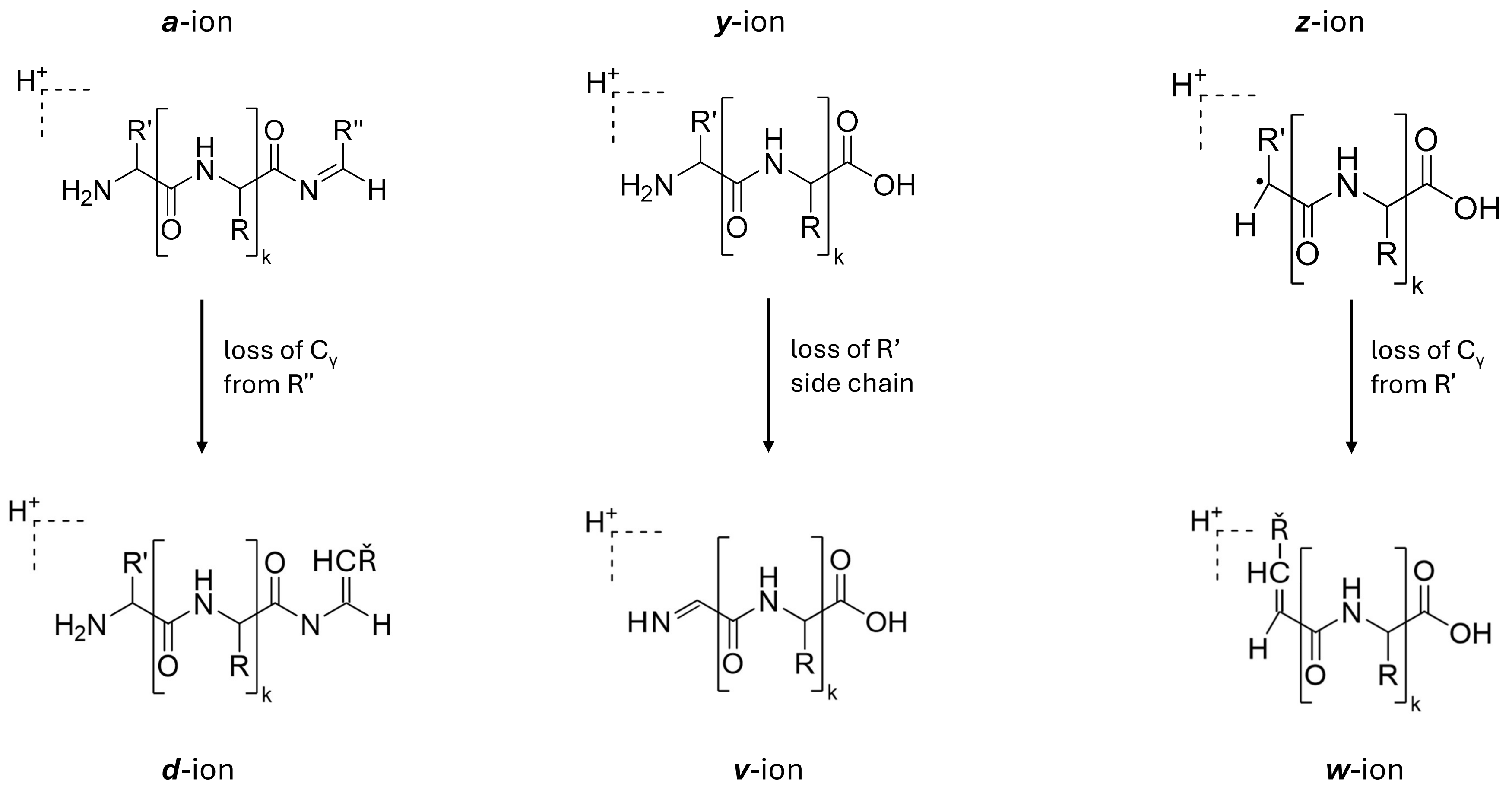

Supplementary Figure S14. Definitions of *d*-, *v*- and *w*-ions. Ř is the substituent (if any) of the side-chain C_β.

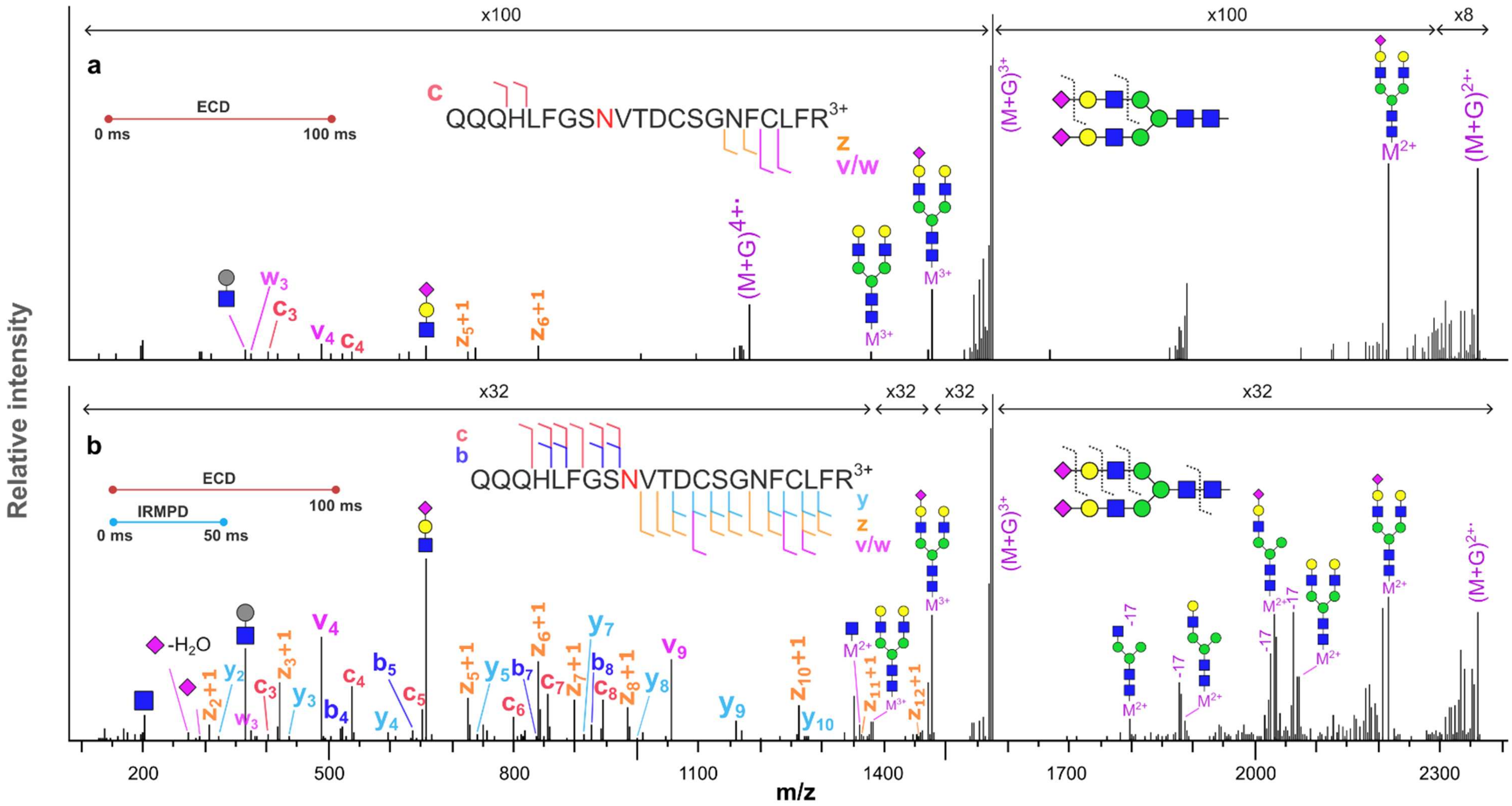

Supplementary Figure S15. ECD **(a)** and AI-ECD **(b)** spectra of N-glycosylated QQQHLFGSNVTDCSGNFCLFR^{3+} acquired in LCMS of a complex glycopeptide mixture. Intact peptide is denoted as M, and precursor ions are annotated as (M+G). All annotations of glycans correspond to *B* or *Y* fragments unless otherwise specified. Precursor ions were irradiated by electrons for 100 ms. In AI-ECD, precursor ions were in addition co-irradiated by IR light at 13 % of the laser duty cycle for the first 50 ms of ECD.

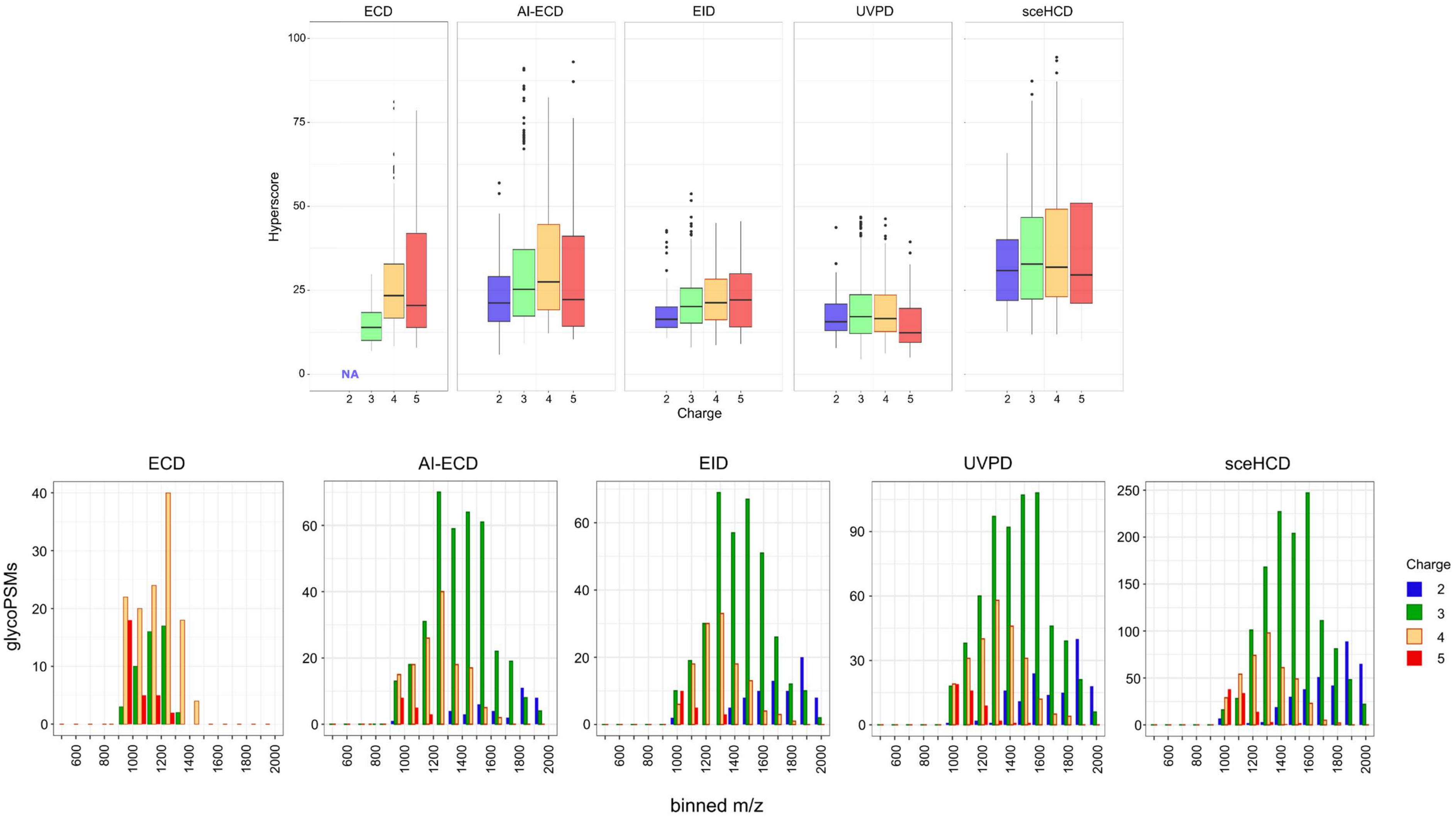

Supplementary Figure S16. **Top:** boxplots of hyperscores per charge state per dissociation technique. **Bottom:** binned distributions of glycoPSM counts per charge state per dissociation technique. Data taken from Supplementary Table S1.

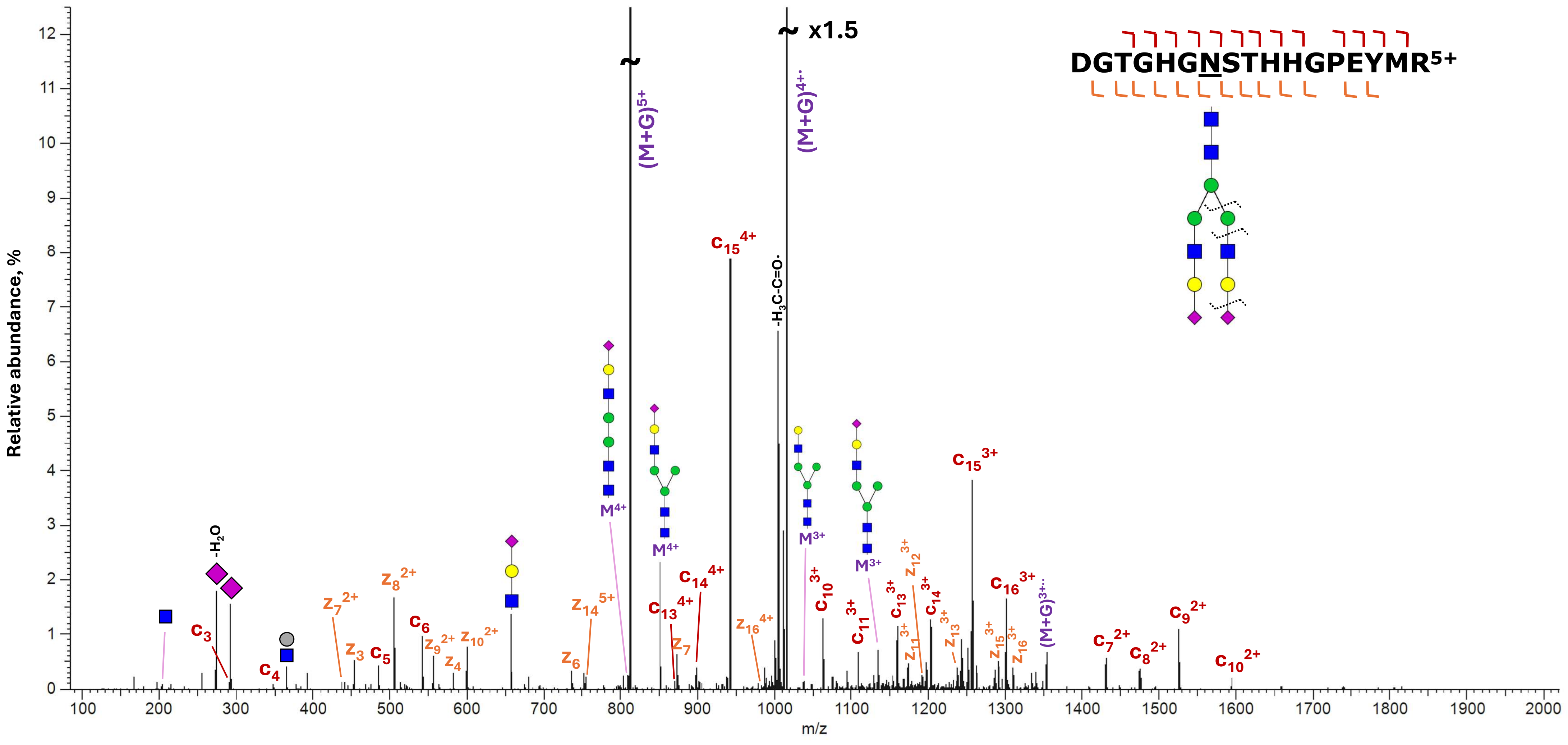

Supplementary Figure S17. ECD spectrum of N-glycosylated NGTGHGNSTHHGPEYMR^{5+} acquired in LCMS analysis of a complex glycopeptide mixture. Precursor ions were irradiated by electrons for 100 ms. Intact peptide is denoted as M, and precursor ions are annotated as (M+G). All annotations of glycans correspond to *B* or *Y* fragments.

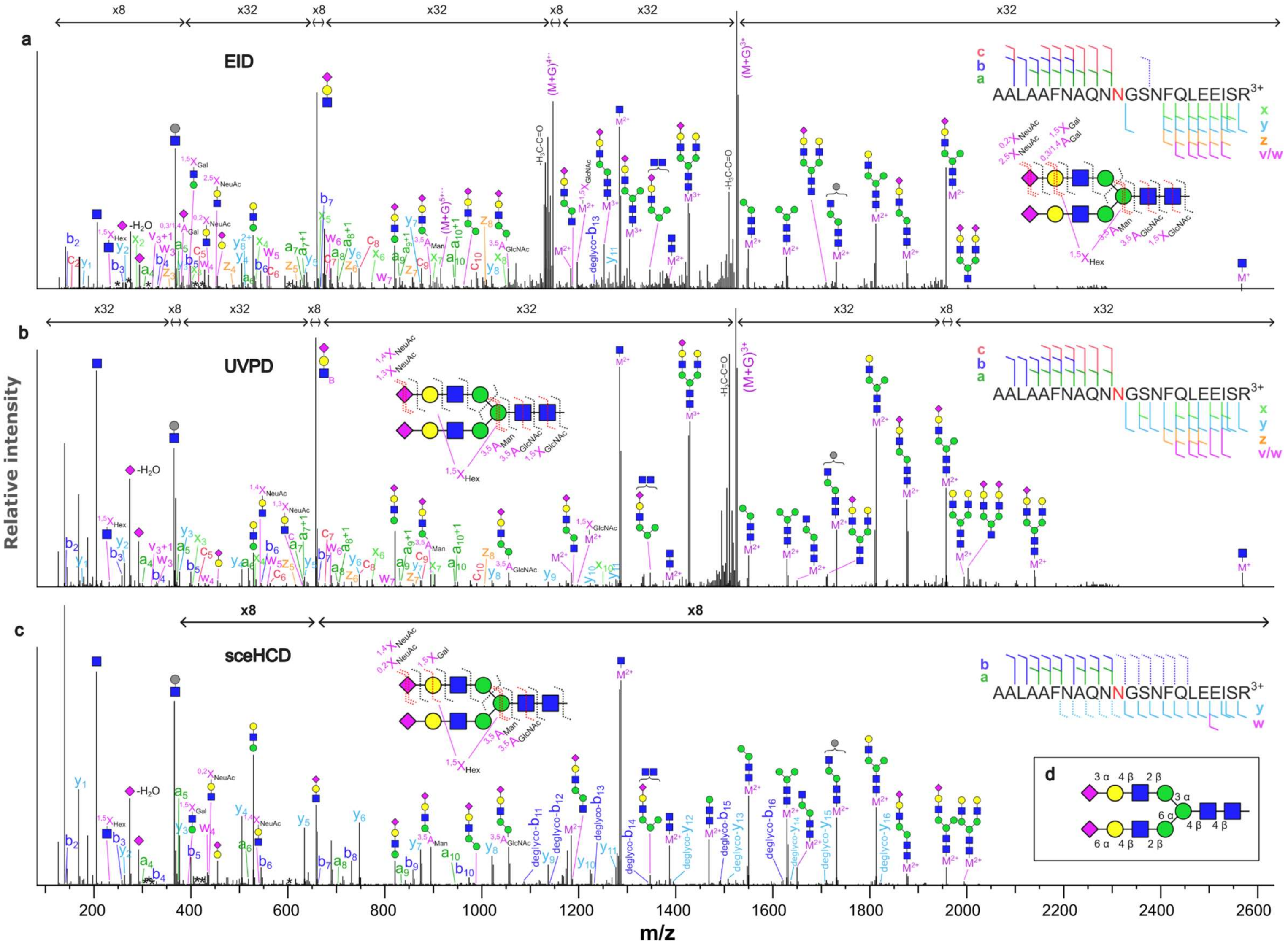

Supplementary Figure S18. EID **(a)**, UVPD **(b)**, and sceHCD **(c)** spectra of N-glycosylated AALAAFNAQNNGSNFQLEEISR^{3+} peptide acquired in the LCMS analysis of a complex glycopeptide mixture. M/z values matching to multiple isobaric internal glycan fragmentation products are marked with asterisks. Intact peptide is denoted as M, and precursor ions are labelled as (M+G). All annotations of glycans correspond to *B* or *Y* fragments unless otherwise specified. Dashed labels correspond to fully deglycosylated peptide fragments. For UVPD, 8 pulses at 8 mJ/pulse were used; in EID, precursor ions were irradiated by 25 eV electrons for 75 ms. Assumed composition of the glycan is shown in (**d**).

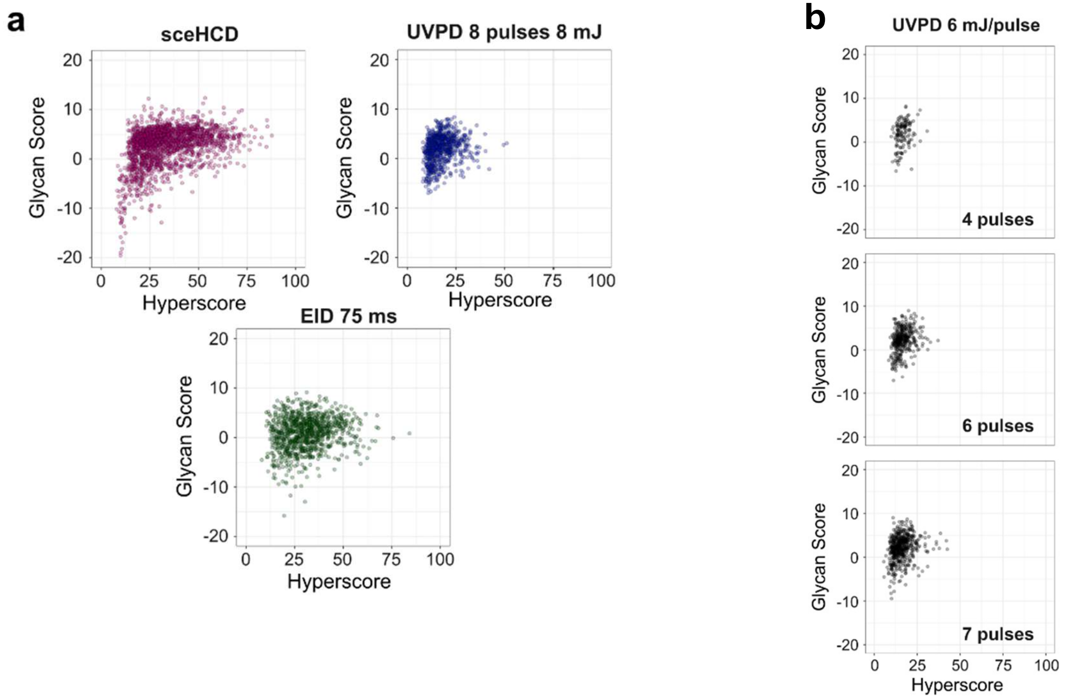

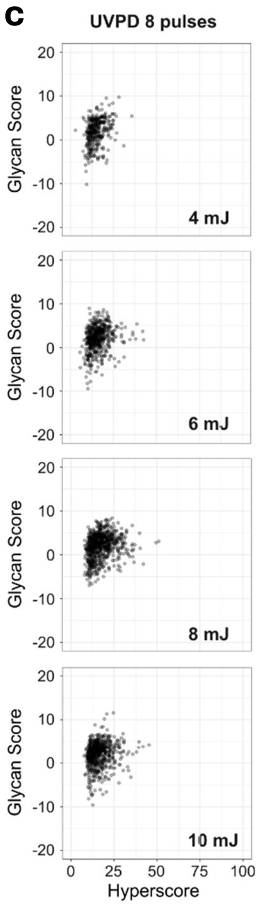

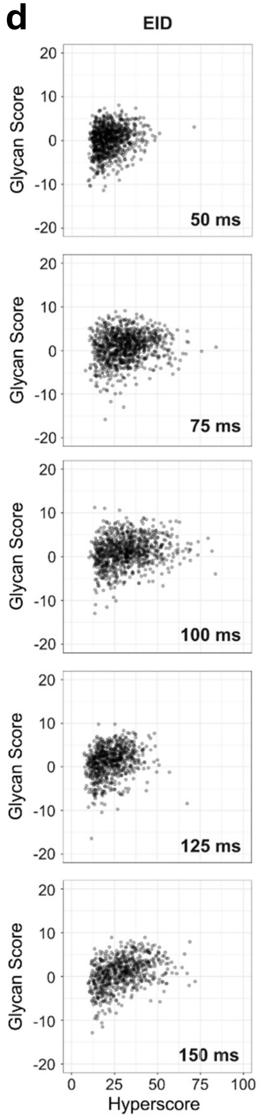

Supplementary Figure S19. a) Glycan scores plotted against hyperscores for glycoPSMs identified in sceHCD experiments, UVPD experiments using b,y peptide fragments for analysis and in EID experiments using b,y,c,z peptide fragments for analysis. **b-d**) Glycan scores plotted against hyperscores for glycoPSMs identified in UVPD using different numbers of laser pulses at 6 mJ/pulse (**b**), 8pulses at various pulse energies (**c**) and in EID experiments in a range of electron irradiation times (**d**). *B,y* types of ions were used in UVPD data analysis, and *b,y,c,z* peptide fragments were used in in the analysis of EID data.

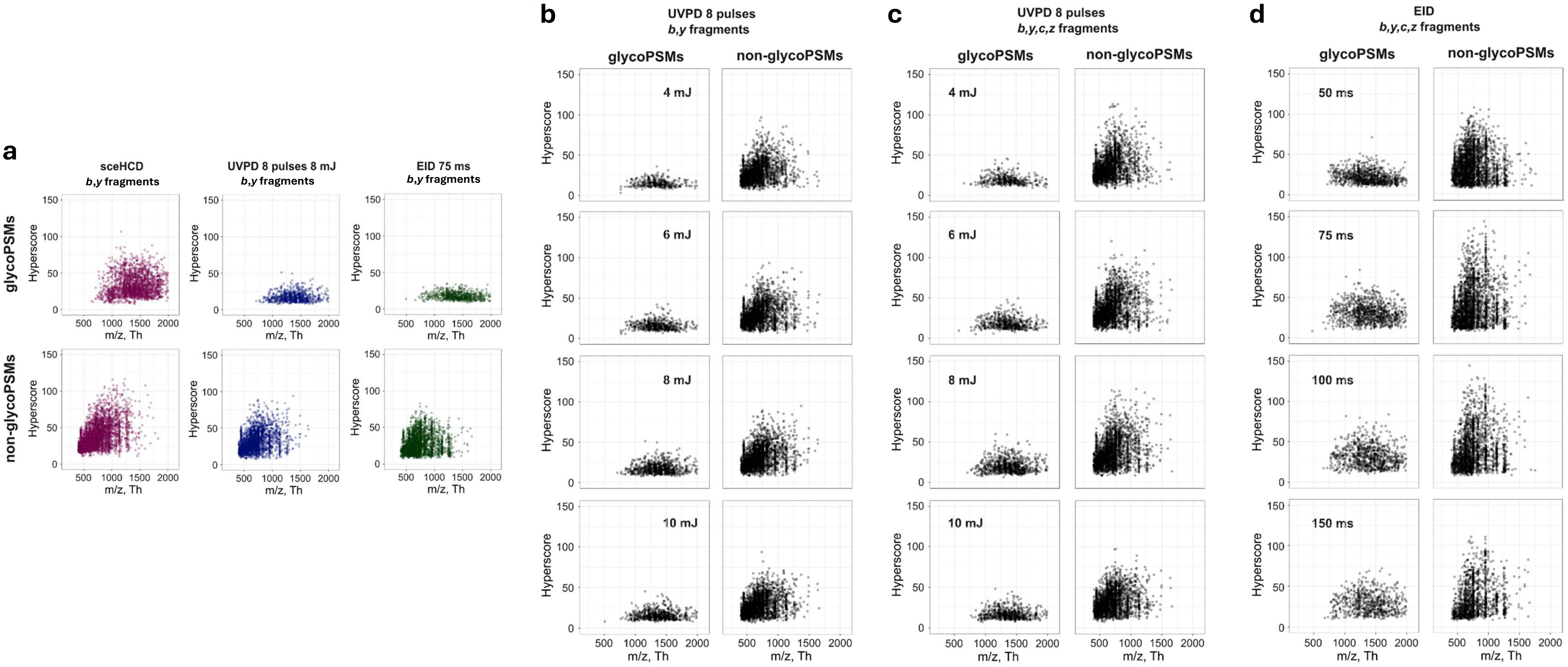

Supplementary Figure S20. Hyperscores plotted against *m/z* of glycoPSMs and non-glycoPSMs identified in sceHCD, UVPD and EID using optimal parameters (**a**), UVPD using 8 pulses at various pulse energies and *b,y* fragments for analysis (**b**), 8 pulses at various pulse energies and *b,y,c,z* fragments for analysis (**c**) and in EID experiments in a range of electron irradiation times and *b,y,c,z* fragments for analysis (**d**).

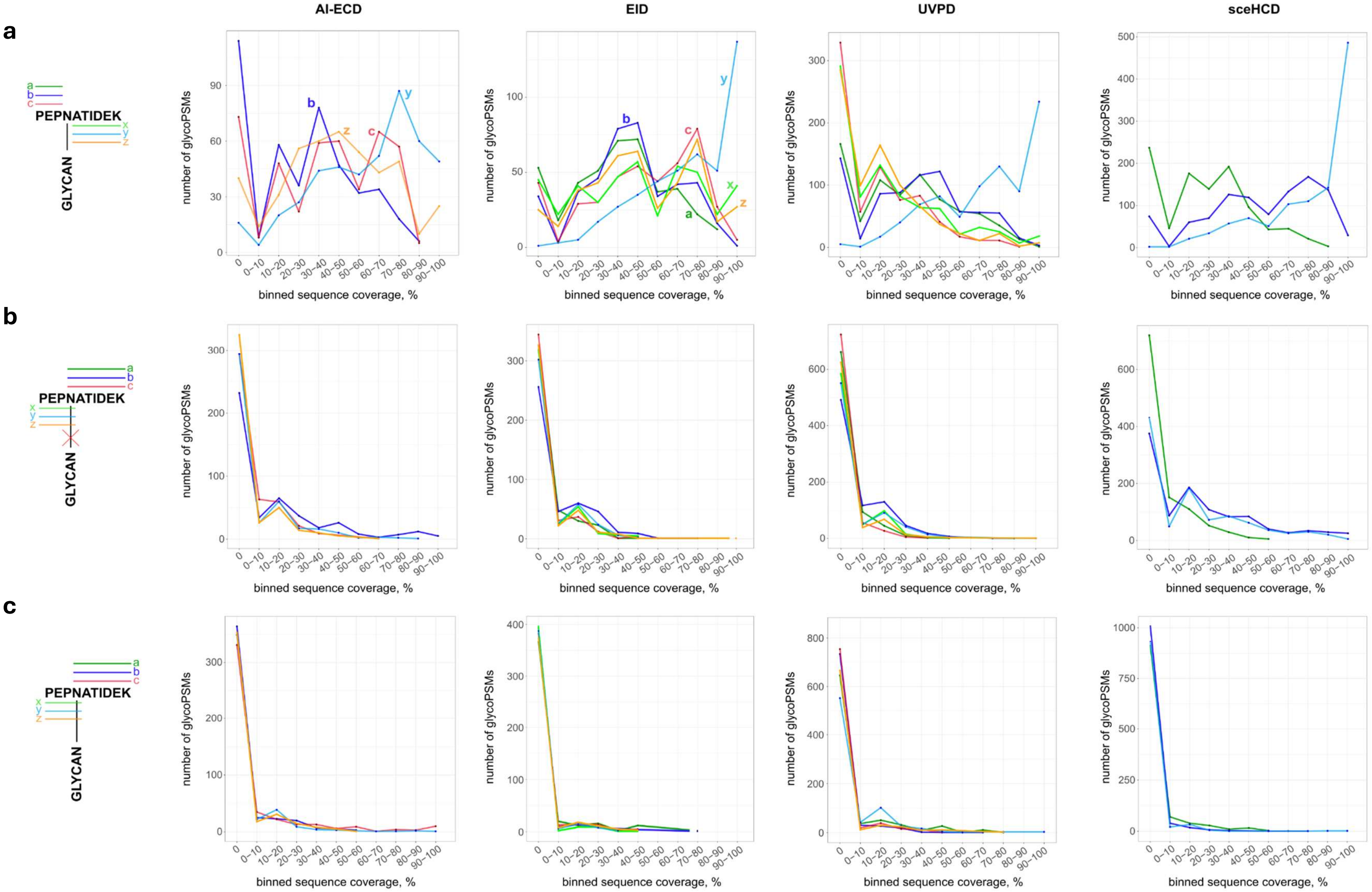

Supplementary Figure S21. Binned coverage of a peptide sequence **a**) N-terminal (by *a*,*b*,*c*, ions) and C-terminal (by *x*,*y*,*z* ions) to an N-glycosylation site; **b**) C-terminal (by *a*,*b*,*c*, ions) and N-terminal (by *x*,*y*,*z* ions) to an N-glycosylation site by deglycosylated ions; **c**) C-terminal (by *a*,*b*,*c*, ions) and N-terminal (by *x*,*y*,*z* ions) to an N-glycosylation site by ions retaining the glycan. Only singly and doubly charged fragment ions were considered for analysis. GlycoPSMs duplicates were filtered to keep the one with the highest hyperscore.

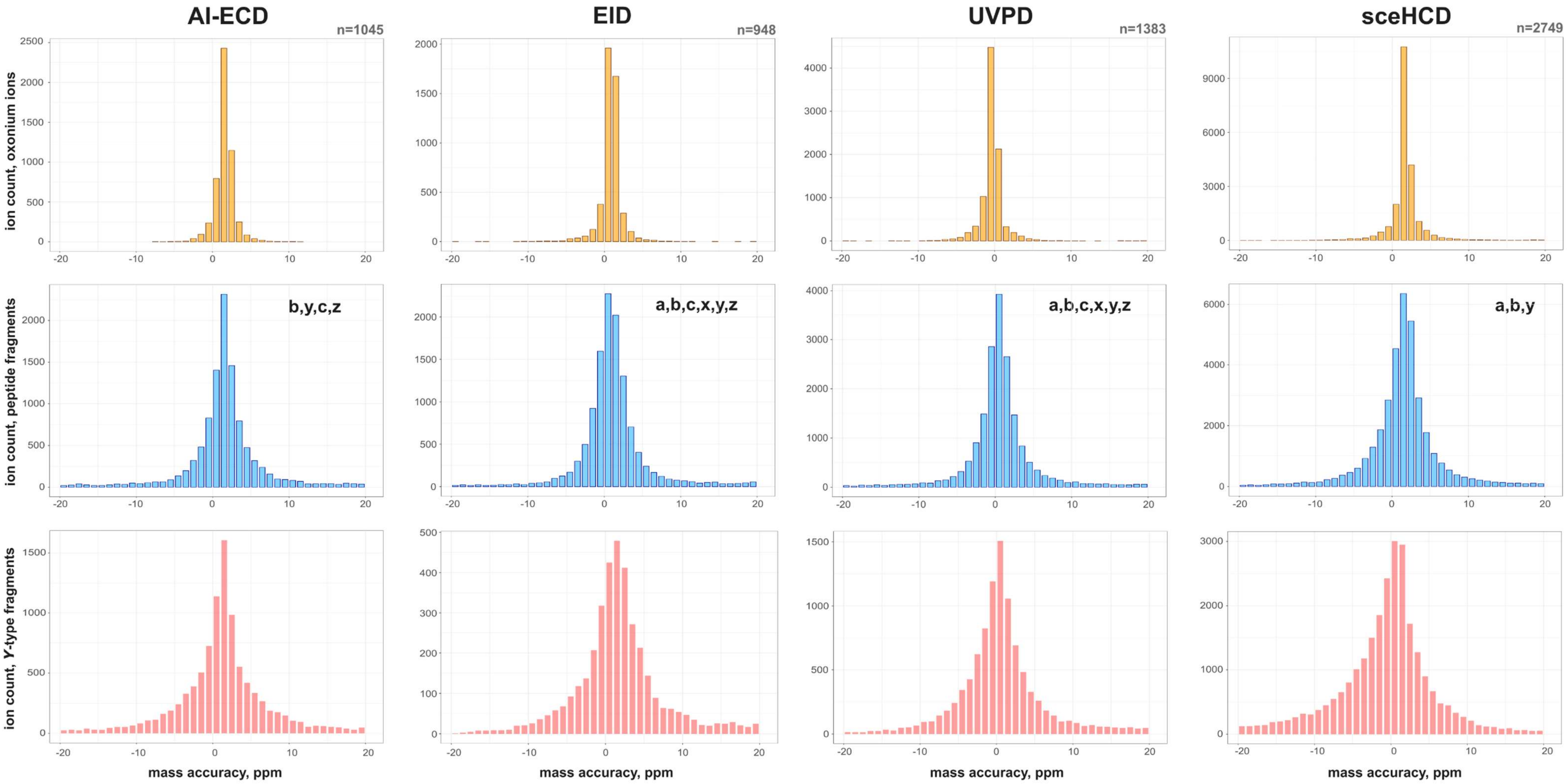

Supplementary Figure S22. Binned mass accuracies (in ppm) of oxonium (top), peptide (middle) and *Y*-type (bottom) 1+ and 2+ fragment ions in AI-ECD, EID, UVPD and sceHCD experiments where optimal parameters and MS1 *m/z* range were used. Only (non-unique) glycoPSMs were analyzed. Types of annotated peptide fragments are given in the middle-row boxes. Only deglycosylated peptide fragments were annotated.

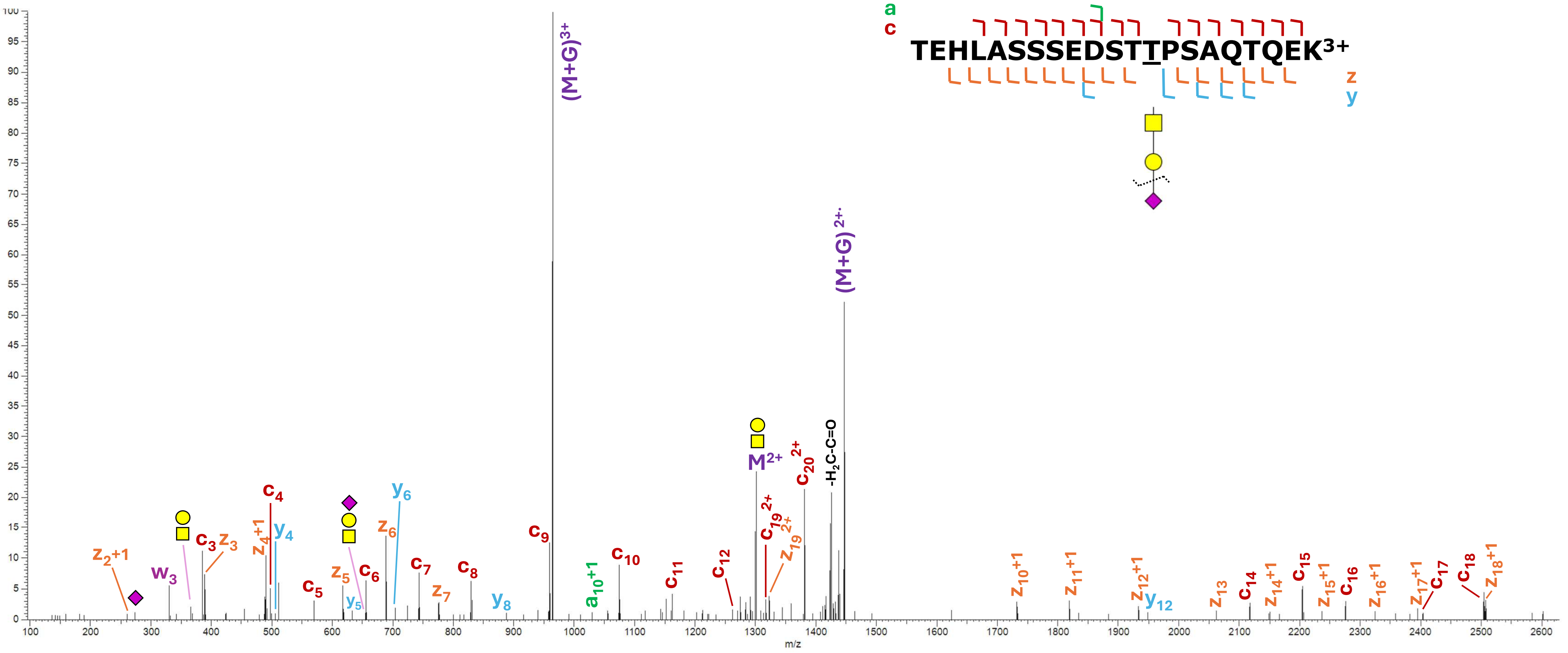

Supplementary Figure S23. AI-ECD spectrum of O-glycosylated TEHLASSSEDST<u>T</u>PSAQTQEK^{3+} acquired in the LCMS analysis of a complex glycopeptide mixture. Intact peptide is denoted as M, and precursor ions are annotated as (M+G). All annotations of glycans correspond to *B* or *Y* fragments. Precursor ions were irradiated by electrons for 100 ms and co-irradiated by IR light at 13 % of the laser duty cycle for the first 50 ms of ECD.

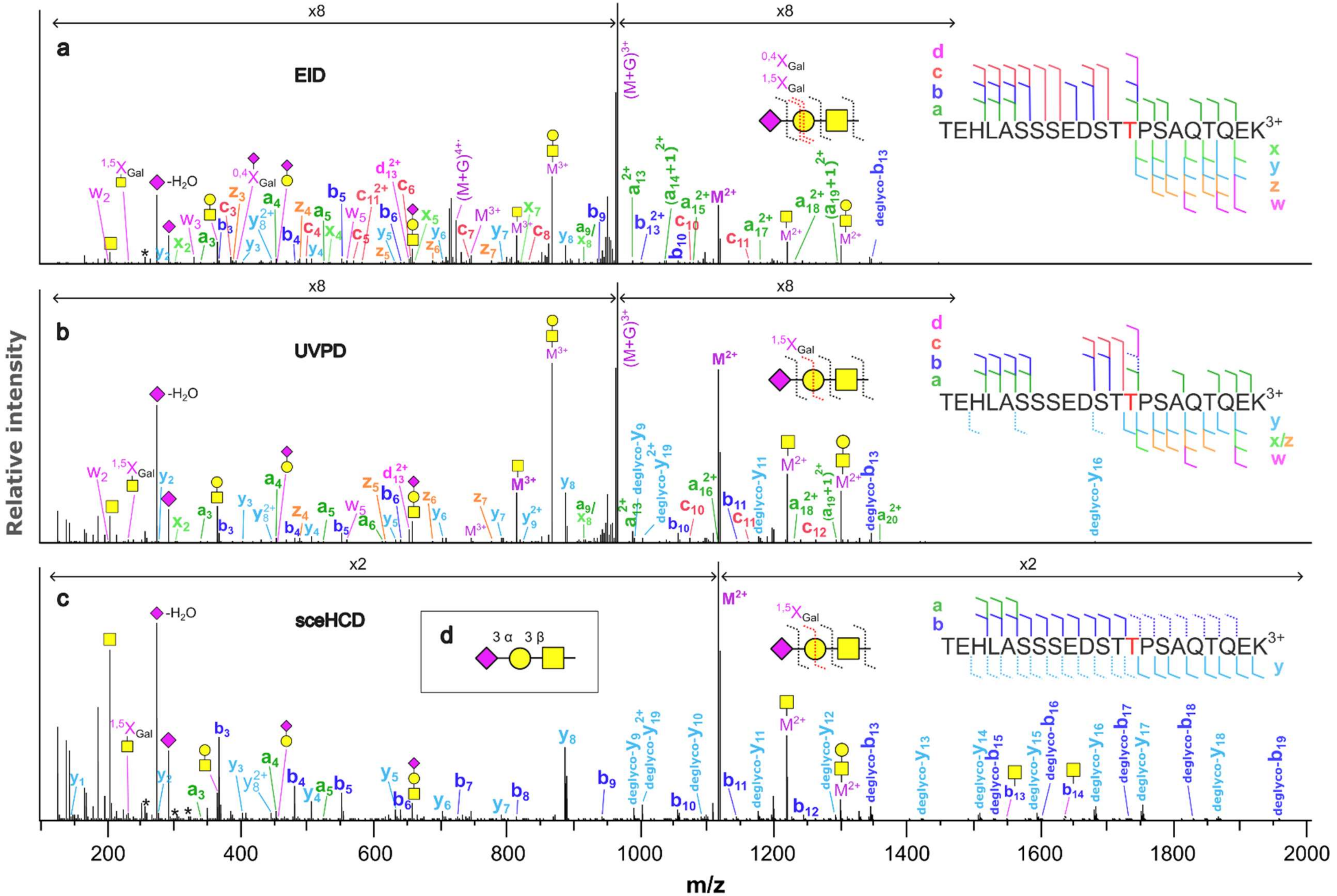

Supplementary Figure S24. UVPD **(a),** EID **(b),** and sceHCD **(c)** spectra of O-glycosylated TEHLASSSEDST<u>T</u>PSAQTQEK^{3+} acquired in the LCMS analysis of a complex glycopeptide mixture. M/z values matching to multiple isobaric internal glycan fragmentation products are marked with asterisks. Intact peptide is denoted as M, and precursor ions are annotated as (M+G). All annotations of glycans correspond to *B* or *Y* fragments unless otherwise specified. Dashed labels correspond to fully deglycosylated peptide fragments. For UVPD, 8 pulses at 8 mJ/pulse were used; in EID, precursor ions were irradiated by 25 eV electrons for 100 ms. Assumed composition of the glycan is shown in (**d**).

Supplementary Table S1. Numbers of non-glycoPSMs and glycoPSMs identified in a complex glycopeptide mixture by each fragmentation technique under optimal experimental parameters.

	fragmentation parameters	non-glycoPSMs	glycoPSMs	number of MS2 scans	glycoPSMs per MS2 scan, %	peptide fragment types used for data analysis
sceHCD	20, 30, 40% NCE	1480	2749	40805	6.7	b,y
ECD	100 ms irradiation	305	206	14683	1.4	b,y,c,z
AI-ECD	50 ms co-irradiation, then 50 ms just ECD	474	1045	14834	7.0	b,y,c,z
UVPD	8 pulses, 8 mJ/pulse	893	1383	20270	6.8	b,y
EID	75 ms irradiation, 25 eV	482	948	16100	5.9	b,y,c,z

Supplementary Table S2. List of O-glycoPSMs identified in a complex glycopeptide mixture by AI-ECD in a standard MS1 range (400-2000 *m/z*) using *b*,*y*,*c*,*z* ions.

Peptide	Charge	Delta Mass	MSFragger Localization
AVGDKLPECEAGATLINEQWLLTTAK	4	1501.5205	AVGDKLPECEAGAtLINEQWLLTTAK
AVGDKLPECEAGATLINEQWLLTTAK	4	1792.6186	AVGDKLPECEAGAtLINEQWLLTTAK
AVGDKLPECEAGATLINEQWLLTTAK	4	2157.7568	AVGDKLPECEAGAtLINEQWLLTTAK
AVGDKLPECEAGATLINEQWLLTTAK	4	1792.6186	AVGDKLPECEAGAtLINEQWLLTTAK
AVGDKLPECEAGATLINEQWLLTTAK	4	1792.6186	AVGDKLPECEAGAtLINEQWLLTTAK
AVGDKLPECEAGATLINEQWLLTTAK	4	2885.998	AVGDKLPECEAGAtLINEQWLLTTAK
AVGDKLPECEAGATLINEQWLLTTAK	3	1792.6123	AVGDKLPECEAGAtLINEQWLLTTAK
CLDGPNTYTCVCTEGYTGTHCEVDIDECDPDPCHYGSCK	4	1395.5004	CLDGPNTYTCVCTEGYTGtHCEVDIDECDPDPCHYGSCK
CLDGPNTYTCVCTEGYTGTHCEVDIDECDPDPCHYGSCK	5	1685.5761	CLDGPNtYtCVCTEGYTGTHCEVDIDECDPDPCHYGSCK
CLDGPNTYTCVCTEGYTGTHCEVDIDECDPDPCHYGSCK	4	1395.5004	CLDGPNTYTCVCTEGYTGTHCEVDIDECDPDPCHYGsCK
EETTVSPPHTSMAPAQDEER	3	1340.4509	EEttVsPPHTSMAPAQDEER
EETTVSPPHTSMAPAQDEER	3	1021.3654	EEttVsPPHtsMAPAQDEER
HYTNPSQDVTVPCPVPPPPPCCHPR	4	2961.1713	HYTNPSQDVtVPCPVPPPPPCCHPR
HYTNPSQDVTVPCPVPPPPPCCHPR	4	2905.1479	HYTNPSQDVtVPCPVPPPPPCCHPR
HYTNPSQDVTVPCPVPSTPPTPSPSTPPTPSPSCCHPR	4	1501.5537	HYTNPSQDVTVPCPVPstPPtPsPstPPtPSPSCCHPR
HYTNPSQDVTVPCPVPSTPPTPSPSTPPTPSPSCCHPR	4	1460.5273	HYTNPSQDVTVPCPVPstPPtPsPSTPPTPSPSCCHPR
HYTNPSQDVTVPCPVPSTPPTPSPSTPPTPSPSCCHPR	4	1501.5507	HYTNPSQDVTVPCPVPStPPtPsPstPPtPsPsCCHPR
HYTNPSQDVTVPCPVPSTPPTPSPSTPPTPSPSCCHPR	4	1663.6079	HYTNPSQDVTVPCPVPstPPtPsPstPPtPsPsCCHPR
HYTNPSQDVTVPCPVPSTPPTPSPSTPPTPSPSCCHPR	4	1298.4799	HYTNPSQDVTVPCPVPstPPtPsPstPPtPsPsCCHPR
HYTNPSQDVTVPCPVPSTPPTPSPSTPPTPSPSCCHPR	4	1751.6108	HYTNPSQDVTVPCPVPstPPtPsPstPPTPSPSCCHPR
HYTNPSQDVTVPCPVPSTPPTPSPSTPPTPSPSCCHPR	4	1954.665	HYTNPSQDVTVPCPVPstPPtPSPSTPPTPSPSCCHPR
HYTNPSQDVTVPCPVPSTPPTPSPSTPPTPSPSCCHPR	4	1825.6557	HYTNPSQDVTVPCPVPstPPtPsPstPPtPsPsCCHPR
HYTNPSQDVTVPCPVPSTPPTPSPSTPPTPSPSCCHPR	4	1589.5703	HYTNPSQDVTVPCPVPstPPtPsPstPPtPsPsCCHPR
HYTNPSQDVTVPCPVPSTPPTPSPSTPPTPSPSCCHPR	4	2098.7045	HYTNPSQDVTVPCPVPstPPtPsPstPPtPsPsCCHPR
HYTNPSQDVTVPCPVPSTPPTPSPSTPPTPSPSCCHPR	4	1792.6416	HYTNPSQDVTVPCPVPstPPtPSPSTPPTPSPSCCHPR
HYTNPSQDVTVPCPVPSTPPTPSPSTPPTPSPSCCHPR	4	1880.6567	HYTNPSQDVTVPCPVPstPPtPSPSTPPTPSPSCCHPR
HYTNPSQDVTVPCPVPSTPPTPSPSTPPTPSPSCCHPR	4	1954.6831	HYTNPSQDVTVPCPVPstPPtPsPstPPtPsPsCCHPR
HYTNPSQDVTVPCPVPSTPPTPSPSTPPTPSPSCCHPR	4	2273.7871	HYTNPSQDVTVPCPVPstPPtPsPstPPtPsPsCCHPR
HYTNPSQDVTVPCPVPSTPPTPSPSTPPTPSPSCCHPR	4	2083.7314	HYTNPSQDVTVPCPVPstPPtPsPstPPtPsPsCCHPR
HYTNPSQDVTVPCPVPSTPPTPSPSTPPTPSPSCCHPR	4	2070.706	HYTNPSQDVTVPCPVPstPPtPsPstPPtPsPsCCHPR
HYTNPSQDVTVPCPVPSTPPTPSPSTPPTPSPSCCHPR	4	2333.7988	HYTNPSQDVTVPCPVPstPPtPsPstPPtPsPsCCHPR
HYTNPSQDVTVPCPVPSTPPTPSPSTPPTPSPSCCHPR	4	2042.7055	HYTNPSQDVTVPCPVPstPPtPsPstPPTPSPSCCHPR
HYTNPSQDVTVPCPVPSTPPTPSPSTPPTPSPSCCHPR	4	2273.7871	HYTNPSQDVTVPCPVPstPPtPsPstPPtPsPsCCHPR
HYTNPSQDVTVPCPVPSTPPTPSPSTPPTPSPSCCHPR	4	2171.746	HYTNPSQDVTVPCPVPstPPtPsPstPPtPsPsCCHPR
HYTNPSQDVTVPCPVPSTPPTPSPSTPPTPSPSCCHPR	4	1807.6098	HYTNPSQDVTVPCPVPstPPtPsPstPPtPsPsCCHPR
HYTNPSQDVTVPCPVPSTPPTPSPSTPPTPSPSCCHPR	4	1908.6518	HYTNPSQDVTVPCPVPstPPtPsPstPPtPsPsCCHPR
HYTNPSQDVTVPCPVPSTPPTPSPSTPPTPSPSCCHPR	4	2300.7876	HYTNPSQDVTVPCPVPstPPtPsPstPPtPsPsCCHPR
HYTNPSQDVTVPCPVPSTPPTPSPSTPPTPSPSCCHPR	4	2273.7866	HYTNPSQDVTVPCPVPstPPtPsPstPPtPsPsCCHPR
HYTNPSQDVTVPCPVPSTPPTPSPSTPPTPSPSCCHPR	4	2245.791	HYTNPSQDVTVPCPVPstPPtPsPstPPtPsPsCCHPR
HYTNPSQDVTVPCPVPSTPPTPSPSTPPTPSPSCCHPR	4	2070.7055	HYTNPSQDVTVPCPVPstPPtPsPstPPtPsPsCCHPR
HYTNPSQDVTVPCPVPSTPPTPSPSTPPTPSPSCCHPR	4	2564.8808	HYTNPSQDVTVPCPVPstPPtPsPstPPtPsPsCCHPR
HYTNPSQDVTVPCPVPSTPPTPSPSTPPTPSPSCCHPR	4	2301.788	HYTNPSQDVTVPCPVPstPPtPsPstPPtPsPsCCHPR
HYTNPSQDVTVPCPVPSTPPTPSPSTPPTPSPSCCHPR	4	2407.8388	HYTNPSQDVTVPCPVPstPPtPsPstPPtPsPsCCHPR
HYTNPSQDVTVPCPVPSTPPTPSPSTPPTPSPSCCHPR	4	2564.8881	HYTNPSQDVTVPCPVPstPPtPsPstPPtPsPsCCHPR
HYTNPSQDVTVPCPVPSTPPTPSPSTPPTPSPSCCHPR	4	2361.8051	HYTNPSQDVTVPCPVPstPPtPsPstPPtPsPsCCHPR
HYTNPSQDVTVPCPVPSTPPTPSPSTPPTPSPSCCHPR	4	1460.5376	HYTNPSQDVTVPCPVPstPPtPsPstPPtPsPsCCHPR
HYTNPSQDVTVPCPVPSTPPTPSPSTPPTPSPSCCHPR	4	1663.6044	HYTNPSQDVTVPCPVPstPPtPsPstPPtPsPsCCHPR

Peptide	Charge	Delta Mass	MSFragger Localization
HYTNSSQDVTVPCR	3	2262.8173	HYtNSSQDVTVPCR
ITELTEAGETK	3	1298.5155	ITELtEAGETK
ITELTEAGETK	3	1298.5155	ITELtEAGETK
ITELTEAGETK	3	1298.5155	ITELtEAGETK
ITELTEAGETK	3	1298.5167	ITELtEAGETK
ITELTEAGETK	3	1298.5172	ITELtEAGETK
ITELTEAGETK	3	1589.6141	ITELtEAGETK
ITELTEAGETK	3	1298.5157	ITELtEAGETK
ITELTEAGETK	3	1298.5155	ITELtEAGETK
LCTDVDECSSGQHQCDSSTVCFNTVGSYSCR	4	2699.9157	LCTDVDECSSGQHQCDSSTVCFNTVGsYsCR
LCTDVDECSSGQHQCDSSTVCFNTVGSYSCR	5	2739.9504	LCTDVDECSSGQHQCDSstVCFNtVGsYsCR
LCTDVDECSSGQHQCDSSTVCFNTVGSYSCR	4	2740.9343	LCTDVDECssGQHQCDsstVCFNtVGsYsCR
LKECCEKPLLEKSHCIAEVENDEMPADLPSLAADFVESK	5	2557.9565	LKECCEKPLLEKsHCIAEVENDEMPADLPSLAADFVESK
LPECEAGATLINEQWLLTTAK	4	2361.9338	LPECEAGAtLINEQWLLTTAK
LPECEAGATLINEQWLLTTAK	4	2262.8674	LPECEAGAtLINEQWLLTTAK
LPECEAGATLINEQWLLTTAK	4	2652.0432	LPECEAGAtLINEQWLLTTAK
LPECEAGATLINEQWLLTTAK	4	2566.0168	LPECEAGAtLINEQWLLTTAK
LPECEAGATLINEQWLLTTAK	4	2652.0407	LPECEAGAtLINEQWLLTTAK
LPECEAGATLINEQWLLTTAK	4	2652.0402	LPECEAGAtLINEQWLLTTAK
LPECEAGATLINEQWLLTTAK	4	2360.9367	LPECEAGAtLINEQWLLTTAK
RSSTDESTYR	3	1460.4957	RsstDESTYR
RTFVLSALQPSPTHSSSNTQR	4	656.2348	RTFVLSALQPsPtHSSSNTQR
SGKQLSDAVYHYDYVHSVR	4	1168.4821	SGKQLsDAVYHYDYVHSVR
SSTDESTYR	4	2259.7348	sstDESTYR
SSTDESTYR	4	1968.6334	sstDESTYR
SSTTKPPFKPHGSR	4	656.2265	SSttKPPFKPHGSR
SSTTKPPFKPHGSR	4	656.2299	sstTKPPFKPHGSR
SSTTKPPFKPHGSR	3	365.1325	SSTtKPPFKPHGSR
SSTTKPPFKPHGSR	4	1312.4558	SSTtKPPFKPHGSR
SSTTKPPFKPHGSR	4	947.3064	ssttKPPFKPHGSR
SWDFGAFQSSSATDSSMEEIFQHLMAYHQEASKR	5	771.2485	SWDFGAFQSSSATDssMEEIFQHLMAYHQEAsKR
TEHLASSSEDSTTPSAQTQEK	3	656.2299	TEHLASSSEDSTtPSAQTQEK
TEHLASSSEDSTTPSAQTQEK	3	1312.4519	TEHLASSSEDsttPsAQTQEK
TFVLSALQPSPTHSSSNTQR	3	656.2268	TFVLSALQPSPtHSSSNTQR
TPEVTCVVVDVSHEDPEVK	4	1706.7168	TPEVTCVVVDVsHEDPEVK
VSSCCGSVSSEQSCGLENCCRPSCCQTTCCR	4	2555.9543	VSSCCGSVSSEQSCGLENCCRPSCCQtTCCR
WECPWHHCDVCGKPSTSFCHLCPNSFCK	5	2869.936	WECPWHHCDVCGKPSTSFCHLCPNsFCK

Supplementary Table S3. List of O-glycoPSMs identified in a complex glycopeptide mixture by 75-ms EID in a standard MS1 range (400-2000 *m/z*) using *b*,*y*,*c*,*z* ions. Table continues on the next page

Peptide	Charge	Delta Mass	MSFragger Localization
RTFVLSALQPSPTHSSSNTQR	4	656.227	RTFVLSALQPsPtHSSSNTQR
IGEIKEETTVSPPHTSMAPAQDEER	4	1312.4565	IGEIKEETTVSPPHtsMAPAQDEER
HYTNPSQDVTVPCPVPSTPPTPSPSTPPTPSPSCCHPR	4	2448.9003	HYTNPSQDVTVPCPVPstPPtPsPSTPPTPSPSCCHPR
IGEIKEETTVSPPHTSMAPAQDEER	4	1968.6867	IGEIKEETTVSPPHtSMAPAQDEER
HYTNPSQDVTVPCPVPSTPPTPSPSTPPTPSPSCCHPR	4	2286.8286	HYTNPSQDVTVPCPVPstPPtPsPSTPPTPSPSCCHPR
HYTNPSQDVTVPCPVPSTPPTPSPSTPPTPSPSCCHPR	4	2698.9707	HYTNPSQDVTVPCPVPstPPtPsPstPPTPSPSCCHPR
HYTNPSQDVTVPCPVPSTPPTPSPSTPPTPSPSCCHPR	4	1663.6167	HYTNPSQDVTVPCPVPstPPtPsPstPPTPSPSCCHPR
LKECCEKPLLEKSHCIAEVENDEMPADLPSLAADFVESK	4	1543.5376	LKECCEKPLLEKsHCIAEVENDEMPADLPSLAADFVESK
IGEIKEETTVSPPHTSMAPAQDEER	4	1312.4587	IGEIKEETTVSPPHtsMAPAQDEER
SHCIAEVENDEMPADLPSLAADFVESK	5	2050.7697	SHCIAEVENDEMPADLPSLAADFVEsK
FSVATQTCQITPAEGPVVTAQYDCLGCVHPISTQSPDLEPILR	4	1312.4482	FSVATQTCQITPAEGPVVtAQYDCLGCVHPIstQsPDLEPILR
IECVSAETTEDCIAK	3	859.3497	IECVsAETTEDCIAK
IECVSAETTEDCIAK	3	859.3497	IECVsAETTEDCIAK
RTFVLSALQPSPTHSSSNTQR	3	656.2299	RTFVLSALQPsPtHSSSNTQR
HYTNPSQDVTVPCPVPSTPPTPSPSTPPTPSPSCCHPR	4	1225.4921	HYTNPSQDVTVPCPVPstPPtPsPstPPTPSPSCCHPR
HYTNPSQDVTVPCPVPSTPPTPSPSTPPTPSPSCCHPR	4	1995.7275	HYTNPSQDVTVPCPVPstPPtPsPstPPtPSPSCCHPR
HYTNPSQDVTVPCPVPSTPPTPSPSTPPTPSPSCCHPR	4	2448.9003	HYTNPSQDVTVPCPVPstPPtPsPsTPPTPSPSCCHPR
HYTNPSQDVTVPCPVPSTPPTPSPSTPPTPSPSCCHPR	4	1386.5	HYTNPSQDVTVPCPVPStPPtPsPstPPTPSPSCCHPR
HYTNPSQDVTVPCPVPSTPPTPSPSTPPTPSPSCCHPR	4	2536.9013	HYTNPSQDVTVPCPVPstPPtPsPstPPtPSPSCCHPR
HYTNPSQDVTVPCPVPSTPPTPSPSTPPTPSPSCCHPR	5	2827.9838	HYTNPSQDVTVPCPVPstPPtPsPstPPtPsPSCCHPR
HYTNPSQDVTVPCPVPSTPPTPSPSTPPTPSPSCCHPR	5	2067.6743	HYTNPSQDVTVPCPVPstPPtPsPstPPTPSPSCCHPR
HYTNPSQDVTVPCPVPSTPPTPSPSTPPTPSPSCCHPR	4	1427.5322	HYTNPSQDVTVPCPVPstPPtPsPstPPtPsPSCCHPR
ELADLSQTSNVLVTPTSVAMAFAMLSLGTK	4	2274.8022	ELADLsQtsNVLVtPtsVAMAFAMLSLGTK
HYTNPSQDVTVPCPVPSTPPTPSPSTPPTPSPSCCHPR	4	1922.6547	HYTNPSQDVTVPCPVPstPPtPsPstPPtPSPSCCHPR
HYTNPSQDVTVPCPVPSTPPTPSPSTPPTPSPSCCHPR	4	2666.9296	HYTNPSQDVTVPCPVPstPPtPsPstPPtPsPsCCHPR
HYTNPSQDVTVPCPVPSTPPTPSPSTPPTPSPSCCHPR	4	1298.4848	HYTNPSQDVTVPCPVPstPPtPsPstPPtPSPSCCHPR
HYTNPSQDVTVPCPVPSTPPTPSPSTPPTPSPSCCHPR	4	1704.6455	HYTNPSQDVTVPCPVPstPPtPsPstPPtPsPSCCHPR
HYTNPSQDVTVPCPVPSTPPTPSPSTPPTPSPSCCHPR	4	2698.8867	HYTNPSQDVTVPCPVPstPPtPsPstPPTPSPSCCHPR
ECCEKPLLEKSHCIAEVENDEMPADLPSLAADFVESK	4	2376.8877	ECCEKPLLEKsHCIAEVENDEMPADLPSLAADFVESK
FSVATQTCQITPAEGPVVTAQYDCLGCVHPISTQSPDLEPILR	3	656.2348	FSVATQTCQITPAEGPVVtAQYDCLGCVHPISTQSPDLEPILR
HYTNPSQDVTVPCPVPSTPPTPSPSTPPTPSPSCCHPR	4	1501.5512	HYTNPSQDVTVPCPVPstPPtPsPstPPtPsPsCCHPR
HYTNPSQDVTVPCPVPSTPPTPSPSTPPTPSPSCCHPR	4	1866.6743	HYTNPSQDVTVPCPVPstPPtPsPstPPtPsPSCCHPR
HYTNPSQDVTVPCPVPSTPPTPSPSTPPTPSPSCCHPR	4	2157.7861	HYTNPSQDVTVPCPVPstPPtPsPstPPtPsPSCCHPR
HYTNPSQDVTVPCPVPSTPPTPSPSTPPTPSPSCCHPR	4	2333.8286	HYTNPSQDVTVPCPVPstPPtPsPstPPTPSPSCCHPR
LHVPLMPAQPAPPKPQPTTRTPPQSQTPGALPAK	5	2624.924	LHVPLMPAQPAPPKPQPttRtPPQsQtPGALPAK
HYTNPSQDVTVPCPVPSTPPTPSPSTPPTPSPSCCHPR	4	1663.6113	HYTNPSQDVTVPCPVPstPPtPsPstPPTPSPSCCHPR
HYTNPSQDVTVPCPVPSTPPTPSPSTPPTPSPSCCHPR	5	2649.852	HYTNPSQDVTVPCPVPstPPtPsPstPPTPSPSCCHPR
HYTNPSQDVTVPCPVPSTPPTPSPSTPPTPSPSCCHPR	4	2286.8286	HYTNPSQDVTVPCPVPstPPtPsPstPPTPSPSCCHPR
HYTNPSQDVTVPCPVPSTPPTPSPSTPPTPSPSCCHPR	4	1339.4702	HYTNPSQDVTVPCPVPstPPtPsPstPPtPSPSCCHPR
HYTNPSQDVTVPCPVPSTPPTPSPSTPPTPSPSCCHPR	4	2259.7788	HYTNPSQDVTVPCPVPstPPtPsPsTPPTPSPSCCHPR
HYTNPSQDVTVPCPVPSTPPTPSPSTPPTPSPSCCHPR	4	2333.8281	HYTNPSQDVTVPCPVPstPPtPsPstPPtPsPsCCHPR
HYTNPSQDVTVPCPVPSTPPTPSPSTPPTPSPSCCHPR	4	2739.9921	HYTNPSQDVTVPCPVPstPPtPsPstPPtPsPsCCHPR
SHCIAEVENDEMPADLPSLAADFVESK	5	2067.7888	SHCIAEVENDEMPADLPSLAADFVEsK
HYTNPSQDVTVPCPVPSTPPTPSPSTPPTPSPSCCHPR	4	2245.7773	HYTNPSQDVTVPCPVPstPPtPsPstPPtPSPSCCHPR
HYTNPSQDVTVPCPVPSTPPTPSPSTPPTPSPSCCHPR	4	2319.8378	HYTNPSQDVTVPCPVPstPPtPsPstPPtPsPSCCHPR
HYTNPSQDVTVPCPVPSTPPTPSPSTPPTPSPSCCHPR	4	2828.0268	HYTNPSQDVTVPCPVPstPPtPsPstPPTPSPSCCHPR
HYTNPSQDVTVPCPVPSTPPTPSPSTPPTPSPSCCHPR	5	2869.0273	HYTNPSQDVTVPCPVPstPPtPsPstPPtPSPSCCHPR
HYTNPSQDVTVPCPVPSTPPTPSPSTPPTPSPSCCHPR	4	1792.6577	HYTNPSQDVTVPCPVPstPPtPsPstPPtPSPSCCHPR
HYTNPSQDVTVPCPVPSTPPTPSPSTPPTPSPSCCHPR	4	1954.7158	HYTNPSQDVTVPCPVPstPPtPsPstPPtPsPSCCHPR
HYTNPSQDVTVPCPVPSTPPTPSPSTPPTPSPSCCHPR	4	1880.666	HYTNPSQDVTVPCPVPstPPtPsPstPPTPSPSCCHPR
LHVPLMPAQPAPPKPQPTTRTPPQSQTPGALPAK	5	1968.6943	LHVPLMPAQPAPPKPQPttRtPPQsQtPGALPAK
HYTNPSQDVTVPCPVPSTPPTPSPSTPPTPSPSCCHPR	4	1630.5908	HYTNPSQDVTVPCPVPstPPtPsPstPPTPSPSCCHPR
HYTNPSQDVTVPCPVPSTPPTPSPSTPPTPSPSCCHPR	4	1460.5263	HYTNPSQDVTVPCPVPstPPtPsPstPPtPsPsCCHPR
HYTNPSQDVTVPCPVPSTPPTPSPSTPPTPSPSCCHPR	4	1954.7148	HYTNPSQDVTVPCPVPstPPtPsPstPPtPSPSCCHPR

Peptide	Charge	Delta Mass	MSFragger Localization
YLHTAVIVSGTMLVFGGNTHNDTSMSHGAK	5	1864.639	YLHTAVIVSGTMLVFGGNtHNDTSMSHGAK
HYTNPSQDVTVPCPVPSTPPTPSPSTPPTPSPSCCHPR	4	1677.588	HYTNPSQDVTVPCPVPStPPtPsPstPPTPSPSCCHPR
HYTNPSQDVTVPCPVPSTPPTPSPSTPPTPSPSCCHPR	4	2042.724	HYTNPSQDVTVPCPVPstPPtPsPstPPTPSPSCCHPR
HYTNPSQDVTVPCPVPSTPPTPSPSTPPTPSPSCCHPR	4	2116.766	HYTNPSQDVTVPCPVPstPPtPsPstPPTPSPSCCHPR
HYTNPSQDVTVPCPVPSTPPTPSPSTPPTPSPSCCHPR	5	2625.905	HYTNPSQDVTVPCPVPstPPtPsPstPPTPSPSCCHPR
HYTNPSQDVTVPCPVPSTPPTPSPSTPPTPSPSCCHPR	4	2901.038	HYTNPSQDVTVPCPVPstPPtPsPstPPtPSPSCCHPR
HYTNPSQDVTVPCPVPSTPPTPSPSTPPTPSPSCCHPR	4	2698.975	HYTNPSQDVTVPCPVPstPPtPsPstPPtPsPsCCHPR
HYTNPSQDVTVPCPVPSTPPTPSPSTPPTPSPSCCHPR	4	2245.812	HYTNPSQDVTVPCPVPstPPtPsPstPPtPsPSCCHPR
HYTNPSQDVTVPCPVPSTPPTPSPSTPPTPSPSCCHPR	4	2157.786	HYTNPSQDVTVPCPVPstPPtPsPstPPtPSPSCCHPR
HYTNPSQDVTVPCPVPSTPPTPSPSTPPTPSPSCCHPR	4	1825.663	HYTNPSQDVTVPCPVPstPPtPsPstPPtPsPSCCHPR
HYTNPSQDVTVPCPVPSTPPTPSPSTPPTPSPSCCHPR	4	2462.875	HYTNPSQDVTVPCPVPstPPtPsPstPPtPSPSCCHPR
VQYECATGYYTAGGK	4	1664.612	VQYECAtGYYTAGGK
ALVLIAFAQYLQQCPFEDHVKLVNEVTEFAK	3	1340.599	ALVLIAFAQYLQQCPFEDHVKLVNEVtEFAK
HYTNPSQDVTVPCPVPSTPPTPSPSTPPTPSPSCCHPR	4	2083.753	HYTNPSQDVTVPCPVPstPPtPsPstPPTPSPSCCHPR
HYTNPSQDVTVPCPVPSTPPTPSPSTPPTPSPSCCHPR	4	1460.535	HYTNPSQDVTVPCPVPstPPtPsPstPPtPSPSCCHPR
HYTNPSQDVTVPCPVPSTPPTPSPSTPPTPSPSCCHPR	4	2374.858	HYTNPSQDVTVPCPVPstPPtPsPstPPTPSPSCCHPR
IGEIKEETTVSPPHTSMAPAQDEER	3	2608.916	IGEIKEEttVsPPHtsMAPAQDEER
HYTNPSQDVTVPCPVPSTPPTPSPSTPPTPSPSCCHPR	4	2917.017	HYTNPSQDVTVPCPVPstPPtPsPstPPTPSPSCCHPR
HYTNPSQDVTVPCPVPSTPPTPSPSTPPTPSPSCCHPR	4	1751.628	HYTNPSQDVTVPCPVPstPPtPsPstPPtPSPSCCHPR
HYTNPSQDVTVPCPVPSTPPTPSPSTPPTPSPSCCHPR	4	2828	HYTNPSQDVTVPCPVPstPPtPsPstPPtPsPsCCHPR
VDNALQSGNSQESVTEQDSKDSTYSLSSTLTLSK	4	2101.781	VDNALQSGNSQESVTEQDsKDSTYSLSSTLTLSK
HYTNPSQDVTVPCPVPSTPPTPSPSTPPTPSPSCCHPR	4	2740.016	HYTNPSQDVTVPCPVPstPPtPsPstPPtPsPsCCHPR
HYTNPSQDVTVPCPVPSTPPTPSPSTPPTPSPSCCHPR	4	2990.063	HYTNPSQDVTVPCPVPstPPtPsPstPPtPsPSCCHPR
HYTNPSQDVTVPCPVPSTPPTPSPSTPPTPSPSCCHPR	4	2610.966	HYTNPSQDVTVPCPVPstPPtPsPstPPtPsPsCCHPR
VKWEYCDVSACSAQDVAYPEESPTEPSTKLPGFDSCGK	4	656.2265	VKWEYCDVSACSAQDVAYPEEsPtEPstKLPGFDSCGK
HYTNPSQDVTVPCPVPSTPPTPSPSTPPTPSPSCCHPR	4	1095.406	HYTNPSQDVTVPCPVPstPPtPsPstPPTPSPSCCHPR
HYTNPSQDVTVPCPVPSTPPTPSPSTPPTPSPSCCHPR	4	2407.874	HYTNPSQDVTVPCPVPstPPtPsPstPPtPsPsCCHPR
ELADLSQTSNVLVTPTSVAMAFAMLSLGTK	3	2274.805	ELADLsQtsNVLVtPtsVAMAFAMLSLGTK
EAKVQWKVDNALQSGNSQESVTEQDSK	3	1265.48	EAKVQWKVDNALQsGNSQESVTEQDSK
LHVPLMPAQPAPPKPQPTTRTPPQSQTPGALPAK	5	2624.935	LHVPLMPAQPAPPKPQPttRtPPQsQtPGALPAK
HYTNPSQDVTVPCPVPSTPPTPSPSTPPTPSPSCCHPR	4	1515.539	HYTNPSQDVTVPCPVPstPPtPsPstPPtPSPSCCHPR
HYTNPSQDVTVPCPVPSTPPTPSPSTPPTPSPSCCHPR	4	2245.812	HYTNPSQDVTVPCPVPstPPtPsPstPPtPsPsCCHPR
LPECEAGATLINEQWLLTTAK	4	2525.016	LPECEAGAtLINEQWLLTTAK
HYTNPSQDVTVPCPVPSTPPTPSPSTPPTPSPSCCHPR	4	2565.892	HYTNPSQDVTVPCPVPstPPtPsPstPPtPsPsCCHPR
HYTNPSQDVTVPCPVPSTPPTPSPSTPPTPSPSCCHPR	4	2624.946	HYTNPSQDVTVPCPVPstPPtPsPstPPTPSPSCCHPR
LHVPLMPAQPAPPKPQPTTRTPPQSQTPGALPAK	4	2624.912	LHVPLMPAQPAPPKPQPttRtPPQsQtPGALPAK
HYTNPSQDVTVPCPVPSTPPTPSPSTPPTPSPSCCHPR	4	2083.753	HYTNPSQDVTVPCPVPstPPtPsPstPPtPSPSCCHPR
HYTNPSQDVTVPCPVPSTPPTPSPSTPPTPSPSCCHPR	4	2536.922	HYTNPSQDVTVPCPVPstPPtPsPstPPtPSPSCCHPR
HYTNPSQDVTVPCPVPSTPPTPSPSTPPTPSPSCCHPR	4	2042.724	HYTNPSQDVTVPCPVPstPPtPsPstPPtPsPsCCHPR
QWINKAVGDKLPECEAGATLINEQWLLTTAK	5	2449.861	QWINKAVGDKLPECEAGAtLINEQWLLTTAK
HYTNPSQDVTVPCPVPSTPPTPSPSTPPTPSPSCCHPR	4	2577.947	HYTNPSQDVTVPCPVPstPPtPsPstPPtPsPsCCHPR
AVGDKLPECEAGATLINEQWLLTTAK	4	2436.866	AVGDKLPECEAGAtLINEQWLLTTAK
FDEFFSEGCAPGSK	4	2885.089	FDEFFsEGCAPGSK
ELADLSQTSNVLVTPTSVAMAFAMLSLGTK	3	2436.861	ELADLsQtsNVLVtPtsVAMAFAMLSLGTK
HYTNPSQDVTVPCPVPSTPPTPSPSTPPTPSPSCCHPR	4	2374.855	HYTNPSQDVTVPCPVPstPPtPsPstPPtPsPsCCHPR
HYTNPSQDVTVPCPVPSTPPTPSPSTPPTPSPSCCHPR	4	2624.935	HYTNPSQDVTVPCPVPstPPtPsPstPPtPsPsCCHPR
GVAHNNLMAMAQETGDNLYWGSVTGSQSNAVSPTPAPR	3	947.3217	GVAHNNLMAMAQETGDNLYWGsVtGsQsNAVSPTPAPR
HYTNPSQDVTVPCPVPSTPPTPSPSTPPTPSPSCCHPR	4	2171.768	HYTNPSQDVTVPCPVPstPPtPsPstPPtPSPSCCHPR
EKHYYIGIIETTWDYASDHGEK	4	2138.838	EKHYYIGIIEttWDYASDHGEK
HYTNPSQDVTVPCPVPSTPPTPSPSTPPTPSPSCCHPR	4	2333.828	HYTNPSQDVTVPCPVPstPPtPsPstPPtPSPSCCHPR
HYTNPSQDVTVPCPVPSTPPTPSPSTPPTPSPSCCHPR	4	1589.573	HYTNPSQDVTVPCPVPstPPtPsPstPPtPsPSCCHPR
ELADLSQTSNVLVTPTSVAMAFAMLSLGTK	3	2304.808	ELADLsQtsNVLVtPtsVAMAFAMLSLGTK
HYTNPSQDVTVPCPVPSTPPTPSPSTPPTPSPSCCHPR	4	1792.659	HYTNPSQDVTVPCPVPstPPtPsPstPPtPsPSCCHPR
HYTNPSQDVTVPCPVPSTPPTPSPSTPPTPSPSCCHPR	4	2990.063	HYTNPSQDVTVPCPVPstPPtPsPstPPtPsPsCCHPR

Supplementary Table S3 (continue). List of O-glycoPSMs identified in a complex glycopeptide mixture by 75-ms EID in a standard MS1 range (400-2000 *m/z*) using *b*,*y*,*c*,*z* ions.

Peptide	Charge	Delta Mass	MSFragger Localization
LPECEAGATLINEQWLLTTAK	4	2508.9387	LPECEAGAtLINEQWLLTTAK
LPECEAGATLINEQWLLTTAK	4	2525.0153	LPECEAGAtLINEQWLLTTAK
MAAAITDMADLEELSR	3	2272.7961	MAAAItDMADLEELSR
LPECEAGATLINEQWLLTTAK	4	2525.0158	LPECEAGAtLINEQWLLTTAK
AGALNSNDAFVLKTPSAAYLWVGTGASEAEK	6	2375.852	AGALNsNDAFVLKTPSAAYLWVGTGASEAEK
RPCFSALEVDETYVPK	3	1104.4423	RPCFsALEVDETYVPK
RGHLFLQTDQPIYNPGQR	4	1544.6359	RGHLFLQtDQPIYNPGQR
HYTNPSQDVTVPCPVPSTPPTPSPSTPPTPSPSCCHPR	4	2828.0263	HYTNPSQDVTVPCPVPstPPtPsPstPPtPsPsCCHPR
SPYEMFGDEEVMCLNGNWTEPPQCK	3	2189.7722	SPYEMFGDEEVMCLNGNWtEPPQCK
HYTNPSQDVTVPCPVPSTPPTPSPSTPPTPSPSCCHPR	4	2407.874	HYTNPSQDVTVPCPVPstPPtPsPstPPtPsPsCCHPR
AVGDKLPECEAGATLINEQWLLTTAK	4	1940.6508	AVGDKLPECEAGAtLINEQWLLTTAK
TPEVTCVVVDVSHEDPEVK	4	1705.7136	TPEVTCVVVDVsHEDPEVK
LSWEYCDLAQCQTPTQAAPPTPVSPR	3	1968.6828	LSWEYCDLAQCQtPtQAAPPTPVSPR
HYTNPSQDVTVPCPVPSTPPTPSPSTPPTPSPSCCHPR	5	2333.8037	HYTNPSQDVTVPCPVPstPPtPsPstPPTPSPSCCHPR
ELADLSQTSNVLVTPTSVAMAFAMLSLGTK	3	2436.8527	ELADLsQtsNVLVtPtsVAMAFAMLSLGTK
TGGGEGEDR	4	1225.4705	tGGGEGEDR
RTFVLSALQPSPTHSSSNTQR	4	947.3371	RTFVLSALQPsPtHsssNTQR
IGEIKEETTVSPPHTSMAPAQDEER	4	2624.9157	IGEIKEEttVsPPHtsMAPAQDEER
HYTNPSQDVTVPCPVPSTPPTPSPSTPPTPSPSCCHPR	5	2374.8344	HYTNPSQDVTVPCPVPstPPtPsPstPPtPsPSCCHPR
FVSVTCDFFESQAPATGSENSAVNQKPTNLPK	3	656.2309	FVSVTCDFFESQAPAtGsENsAVNQKPTNLPK
AVGDKLPECEAGATLINEQWLLTTAK	4	2071.7075	AVGDKLPECEAGAtLINEQWLLTTAK
FPNDHKYTPTLRNGLEENFCR	5	1894.6892	FPNDHKYtPtLRNGLEENFCR
AVGDKLPECEAGATLINEQWLLTTAK	4	2872.9995	AVGDKLPECEAGAtLINEQWLLTTAK
LPECEAGATLINEQWLLTTAK	4	2524.9567	LPECEAGAtLINEQWLLTTAK
RPSEIVIGQCK	3	2319.7556	RPsEIVIGQCK
SCDTPPPCPR	2	947.3209	SCDtPPPCPR
DAALFHSDDTDHCSFSTGCPCSSLAR	4	1394.4526	DAALFHsDDTDHCSFSTGCPCSSLAR
LPECEAGATLINEQWLLTTAK	4	2524.9528	LPECEAGAtLINEQWLLTTAK
ALQAVYSMMSWPDDVPPEGWNR	3	2188.8044	ALQAVYSMMsWPDDVPPEGWNR
AVGDKLPECEAGATLINEQWLLTTAK	5	2099.6762	AVGDKLPECEAGAtLINEQWLLTTAK
MAGLLGAFSPTPAPR	3	2553.9594	MAGLLGAFsPTPAPR
LSPPHGGSSRLLPTQPHLSSPLSK	4	1064.3879	LSPPHGGssRLLPtQPHLSSPLSK
HYTNPSQDVTVPCPVPSTPPTPSPSTPPTPSPSCCHPR	4	2171.7675	HYTNPSQDVTVPCPVPstPPtPsPsTPPTPSPSCCHPR
HSQGQGALGPRPSLAGTSSLAHSTCR	4	1064.3501	HSQGQGALGPRPsLAGtsSLAHSTCR
HYTNPSQDVTVPCPVPSTPPTPSPSTPPTPSPSCCHPR	5	2245.8027	HYTNPSQDVTVPCPVPstPPtPsPstPPtPsPSCCHPR
HYTNPSQDVTVPCPVPSTPPTPSPSTPPTPSPSCCHPR	4	1833.6704	HYTNPSQDVTVPCPVPstPPtPsPstPPtPsPsCCHPR
EVGILVSPDNDIYIAGGYRPSSSEVSIDHK	6	2978.1123	EVGILVSPDNDIYIAGGYRPsSSEVSIDHK
ALQAVYSMMSWPDDVPPEGWNR	3	2188.7907	ALQAVYSMMsWPDDVPPEGWNR
AVGDKLPECEAGATLINEQWLLTTAK	4	2099.6733	AVGDKLPECEAGAtLINEQWLLTTAK
HYTNASDGLCTRLSRPCQTQKPQKPWWEDEWEVPR	6	2050.6142	HYTNASDGLCtRLsRPCQtQKPQKPWWEDEWEVPR
ALQAVYSMMSWPDDVPPEGWNR	3	2172.7651	ALQAVYsMMsWPDDVPPEGWNR
HYTNPSQDVTVPCPVPSTPPTPSPSTPPTPSPSCCHPR	5	2374.8276	HYTNPSQDVTVPCPVPstPPtPsPstPPtPsPsCCHPR
TARSNMDNMFESYINNLR	4	2976.9948	tARsNMDNMFESYINNLR
VVPDSTPSWANPSPTPVISMLAQGSQVLESTPPPHVMR	4	2608.9189	VVPDSTPSWANPsPtPVIsMLAQGsQVLEstPPPHVMR
RLAILPASAPPATSNPDPAVSR	3	2259.8027	RLAILPAsAPPAtsNPDPAVSR
HYTNPSQDVTVPCPVPSTPPTPSPSTPPTPSPSCCHPR	5	2536.8369	HYTNPSQDVTVPCPVPstPPtPsPstPPtPsPsCCHPR
APVDLLGVAHNNLMAMAQETGDNLYWGSVTGSQSNAVSPTPAPR	3	947.3295	APVDLLGVAHNNLMAMAQETGDNLYWGsVtGsQsNAVSPTPAPR
FSVATQTCQITPAEGPVVTAQYDCLGCVHPISTQSPDLEPILR	4	656.2343	FSVATQTCQITPAEGPVVtAQYDCLGCVHPIstQsPDLEPILR
RLAILPASAPPATSNPDPAVSR	3	2259.7187	RLAILPAsAPPAtsNPDPAVSR
HSQGQGALGPRPSLAGTSSLAHSTCR	4	1064.3452	HSQGQGALGPRPsLAGtssLAHSTCR
WQCIECKSCSLCGTSENDGASWAGLTPQDQLLFCDDCDR	4	610.1879	WQCIECKSCSLCGTSENDGASWAGLtPQDQLLFCDDCDR
HYTNPSQDVTVPCPVPSTPPTPSPSTPPTPSPSCCHPR	4	1968.6806	HYTNPSQDVTVPCPVPstPPtPsPstPPtPsPsCCHPR
EESSSVAMLVPDIGEQEAILTAESIISPSLEIDEQR	5	989.3996	EESSSVAMLVPDIGEQEAILTAEsIISPSLEIDEQR
SNMDNMFESYINNLR	3	2962.018	sNMDNMFESYINNLR

Peptide	Charge	Total Glycan Composition	MSFragger Localization
AVGDKLPECEAGATLINEQWLLTTAK	4	HexNAc(5)Hex(3)NeuAc(1) % 1792.6507	AVGDKLPECEAGAtLINEQWLLTTAK
CVPPGDCASHPCQNGGSCEPGLHSGFTCSCPDSHTGR	4	HexNAc(4)Hex(2)NeuAc(4) % 2300.8048	CVPPGDCASHPCQNGGSCEPGLHSGFTCSCPDsHTGR
EETTVSPPHTSMAPAQDEER	3	HexNAc(2)Hex(2)NeuAc(2) % 1312.4552	EEttVSPPHTSMAPAQDEER
EETTVSPPHTSMAPAQDEER	3	HexNAc(2)Hex(2)NeuAc(1) % 1021.3598	EETTVSPPHtsMAPAQDEER
EETTVSPPHTSMAPAQDEER	3	HexNAc(2)Hex(2)NeuAc(1) % 1021.3598	EEttVsPPHTSMAPAQDEER
EETTVSPPHTSMAPAQDEER	3	HexNAc(2)Hex(2)NeuAc(2) % 1312.4552	EEttVsPPHtSMAPAQDEER
EETTVSPPHTSMAPAQDEER	3	HexNAc(2)Hex(2)NeuAc(2) % 1312.4552	EEttVsPPHTSMAPAQDEER
ELADLSQTSNVLVTPTSVAMAFAMLSLGTK	3	HexNAc(4)Hex(2)NeuAc(4) % 2300.8048	ELADLsQtsNVLVtPtsVAMAFAMLSLGTK
HYTNPSQDVTVPCPVPSTPPTPSPSTPPTPSPSCCHPR	4	HexNAc(6)Hex(4) % 1866.6875	HYTNPSQDVTVPCPVPstPPtPsPstPPtPSPSCCHPR
HYTNPSQDVTVPCPVPSTPPTPSPSTPPTPSPSCCHPR	4	HexNAc(5)Hex(5) % 1825.6610	HYTNPSQDVTVPCPVPstPPtPsPstPPtPSPSCCHPR
HYTNPSQDVTVPCPVPSTPPTPSPSTPPTPSPSCCHPR	4	HexNAc(5)Hex(4) % 1663.6081	HYTNPSQDVTVPCPVPstPPtPsPstPPtPsPSCCHPR
HYTNPSQDVTVPCPVPSTPPTPSPSTPPTPSPSCCHPR	4	HexNAc(6)Hex(3) % 1704.6347	HYTNPSQDVTVPCPVPstPPtPsPstPPTPSPSCCHPR
HYTNPSQDVTVPCPVPSTPPTPSPSTPPTPSPSCCHPR	3	HexNAc(5)Hex(4) % 1663.6081	HYTNPSQDVTVPCPVPstPPtPsPstPPTPSPSCCHPR
HYTNPSQDVTVPCPVPSTPPTPSPSTPPTPSPSCCHPR	4	HexNAc(5)Hex(4) % 1663.6081	HYTNPSQDVTVPCPVPstPPtPsPstPPtPSPSCCHPR
HYTNPSQDVTVPCPVPSTPPTPSPSTPPTPSPSCCHPR	4	HexNAc(5)Hex(3) % 1501.5553	HYTNPSQDVTVPCPVPstPPtPsPstPPtPSPSCCHPR
HYTNPSQDVTVPCPVPSTPPTPSPSTPPTPSPSCCHPR	4	HexNAc(5)Hex(5)NeuAc(1) % 2116.7564	HYTNPSQDVTVPCPVPstPPtPSPSTPPTPSPSCCHPR
HYTNPSQDVTVPCPVPSTPPTPSPSTPPTPSPSCCHPR	4	HexNAc(6)Hex(4)NeuAc(1) % 2157.7829	HYTNPSQDVTVPCPVPstPPtPsPstPPTPSPSCCHPR
HYTNPSQDVTVPCPVPSTPPTPSPSTPPTPSPSCCHPR	4	HexNAc(5)Hex(5)NeuAc(1) % 2116.7564	HYTNPSQDVTVPCPVPstPPtPsPstPPtPsPSCCHPR
HYTNPSQDVTVPCPVPSTPPTPSPSTPPTPSPSCCHPR	4	HexNAc(4)Hex(3) % 1298.4759	HYTNPSQDVTVPCPVPstPPtPsPstPPtPSPSCCHPR
HYTNPSQDVTVPCPVPSTPPTPSPSTPPTPSPSCCHPR	4	HexNAc(3)Hex(3) % 1095.3966	HYTNPSQDVTVPCPVPstPPtPsPSTPPTPSPSCCHPR
HYTNPSQDVTVPCPVPSTPPTPSPSTPPTPSPSCCHPR	4	HexNAc(5)Hex(2) % 1339.5025	HYTNPSQDVTVPCPVPstPPtPsPstPPTPSPSCCHPR
HYTNPSQDVTVPCPVPSTPPTPSPSTPPTPSPSCCHPR	4	HexNAc(4)Hex(4)NeuAc(1) % 1751.6242	HYTNPSQDVTVPCPVPstPPtPsPstPPTPSPSCCHPR
HYTNPSQDVTVPCPVPSTPPTPSPSTPPTPSPSCCHPR	4	HexNAc(5)Hex(3)NeuAc(1) % 1792.6507	HYTNPSQDVTVPCPVPstPPtPsPstPPTPSPSCCHPR
HYTNPSQDVTVPCPVPSTPPTPSPSTPPTPSPSCCHPR	4	HexNAc(6)Hex(4)NeuAc(3) % 2739.9738	HYTNPSQDVTVPCPVPstPPtPsPstPPtPSPSCCHPR
HYTNPSQDVTVPCPVPSTPPTPSPSTPPTPSPSCCHPR	4	HexNAc(6)Hex(3)NeuAc(2) % 2286.8255	HYTNPSQDVTVPCPVPstPPtPsPstPPtPSPSCCHPR
HYTNPSQDVTVPCPVPSTPPTPSPSTPPTPSPSCCHPR	5	HexNAc(5)Hex(4)NeuAc(2) % 2245.7990	HYTNPSQDVTVPCPVPstPPtPsPstPPTPSPSCCHPR
HYTNPSQDVTVPCPVPSTPPTPSPSTPPTPSPSCCHPR	4	HexNAc(4)Hex(2) % 1136.4231	HYTNPSQDVTVPCPVPstPPTPSPSTPPTPSPSCCHPR
HYTNPSQDVTVPCPVPSTPPTPSPSTPPTPSPSCCHPR	4	HexNAc(5)Hex(3)NeuAc(2) % 2083.7462	HYTNPSQDVTVPCPVPstPPtPsPstPPtPSPSCCHPR
HYTNPSQDVTVPCPVPSTPPTPSPSTPPTPSPSCCHPR	4	HexNAc(5)Hex(4)NeuAc(2) % 2245.7990	HYTNPSQDVTVPCPVPstPPtPsPstPPtPSPSCCHPR
HYTNPSQDVTVPCPVPSTPPTPSPSTPPTPSPSCCHPR	4	HexNAc(4)Hex(4)NeuAc(1) % 1751.6242	HYTNPSQDVTVPCPVPstPPtPsPSTPPTPSPSCCHPR
HYTNPSQDVTVPCPVPSTPPTPSPSTPPTPSPSCCHPR	5	HexNAc(5)Hex(4)NeuAc(3) % 2536.8944	HYTNPSQDVTVPCPVPstPPtPsPstPPTPSPSCCHPR
HYTNPSQDVTVPCPVPSTPPTPSPSTPPTPSPSCCHPR	4	HexNAc(3)Hex(3)NeuAc(1) % 1386.4920	HYTNPSQDVTVPCPVPstPPtPsPstPPTPSPSCCHPR
HYTNPSQDVTVPCPVPSTPPTPSPSTPPTPSPSCCHPR	4	HexNAc(5)Hex(3)NeuAc(2) % 2083.7462	HYTNPSQDVTVPCPVPstPPtPsPstPPtPSPSCCHPR
HYTNPSQDVTVPCPVPSTPPTPSPSTPPTPSPSCCHPR	4	HexNAc(4)Hex(4)NeuAc(1) % 1751.6242	HYTNPSQDVTVPCPVPstPPtPsPstPPTPSPSCCHPR
HYTNPSQDVTVPCPVPSTPPTPSPSTPPTPSPSCCHPR	4	HexNAc(4)Hex(3)NeuAc(1) % 1589.5714	HYTNPSQDVTVPCPVPstPPtPsPSTPPTPSPSCCHPR
HYTNPSQDVTVPCPVPSTPPTPSPSTPPTPSPSCCHPR	4	HexNAc(4)Hex(4)NeuAc(3) % 2333.8150	HYTNPSQDVTVPCPVPstPPtPsPstPPtPSPSCCHPR
HYTNPSQDVTVPCPVPSTPPTPSPSTPPTPSPSCCHPR	4	HexNAc(4)Hex(3)NeuAc(2) % 1880.6668	HYTNPSQDVTVPCPVPstPPtPsPstPPtPSPSCCHPR
HYTNPSQDVTVPCPVPSTPPTPSPSTPPTPSPSCCHPR	4	HexNAc(4)Hex(2)NeuAc(1) % 1427.5185	HYTNPSQDVTVPCPVPstPPTPSPSTPPTPSPSCCHPR
HYTNPSQDVTVPCPVPSTPPTPSPSTPPTPSPSCCHPR	4	HexNAc(3)Hex(2)NeuAc(1) % 1224.4392	HYTNPSQDVTVPCPVPstPPtPsPstPPTPSPSCCHPR
HYTNPSQDVTVPCPVPSTPPTPSPSTPPTPSPSCCHPR	4	HexNAc(5)Hex(3)NeuAc(3) % 2374.8416	HYTNPSQDVTVPCPVPstPPtPsPstPPtPSPSCCHPR
HYTNPSQDVTVPCPVPSTPPTPSPSTPPTPSPSCCHPR	4	HexNAc(4)Hex(3)NeuAc(3) % 2171.7622	HYTNPSQDVTVPCPVPstPPtPsPSTPPTPSPSCCHPR
HYTNPSQDVTVPCPVPSTPPTPSPSTPPTPSPSCCHPR	4	HexNAc(5)Hex(4)NeuAc(4) % 2827.9898	HYTNPSQDVTVPCPVPstPPtPsPstPPTPSPSCCHPR
HYTNPSQDVTVPCPVPSTPPTPSPSTPPTPSPSCCHPR	4	HexNAc(4)Hex(4)NeuAc(2) % 2042.7196	HYTNPSQDVTVPCPVPstPPtPsPstPPtPsPsCCHPR
HYTNPSQDVTVPCPVPSTPPTPSPSTPPTPSPSCCHPR	4	HexNAc(4)Hex(3)NeuAc(2) % 1880.6668	HYTNPSQDVTVPCPVPstPPtPsPstPPtPSPSCCHPR
HYTNPSQDVTVPCPVPSTPPTPSPSTPPTPSPSCCHPR	4	HexNAc(4)Hex(1)NeuAc(5) % 2429.8474	HYTNPSQDVTVPCPVPstPPtPsPstPPTPSPSCCHPR
RTFVLSALQPSPTHSSSNTQR	4	HexNAc(1)Hex(1)NeuAc(1) % 656.2276	RTFVLSALQPsPTHSSSNTQR
SCDTPPPCPR	2	HexNAc(1)Hex(1)NeuAc(2) % 947.3230	SCDtPPPCPR
SHCIAEVENDEMPADLPSLAADFVESK	3	HexNAc(3)Hex(2)NeuAc(2) % 1515.5346	SHCIAEVENDEMPADLPsLAADFVESK
SPDESTPELSAEPTPK	2	HexNAc(1)Hex(1)NeuAc(1) % 656.2276	SPDESTPELSAEPtPK
SSTTKPPFKPHGSR	4	HexNAc(1)Hex(1)NeuAc(1) % 656.2276	ssTTKPPFKPHGSR
SSTTKPPFKPHGSR	3	HexNAc(1)Hex(1) % 365.1322	ssttKPPFKPHGSR
SSVITLNTNAELFNQSDIVAHLLSSSSSVIDALQYK	3	HexNAc(6)Hex(4) % 1866.6875	SSVITLNTNAELFNQsDIVAHLLssSSSVIDALQYK
TEHLASSSEDSTTPSAQTQEK	3	HexNAc(2)Hex(2)NeuAc(1) % 1021.3598	TEHLASSSEDsttPSAQTQEK
TEHLASSSEDSTTPSAQTQEK	3	HexNAc(2)Hex(2)NeuAc(2) % 1312.4552	TEHLASSSEDSTtPSAQTQEK
TEHLASSSEDSTTPSAQTQEK	3	HexNAc(2)Hex(2)NeuAc(3) % 1603.5506	TEHLASSSEDSTTPsAQTQEK
TEHLASSSEDSTTPSAQTQEK	3	HexNAc(1)Hex(1)NeuAc(2) % 947.3230	TEHLASSSEDSTtPSAQTQEK
TEHLASSSEDSTTPSAQTQEK	3	HexNAc(1)Hex(1)NeuAc(1) % 656.2276	TEHLASSSEDstTPSAQTQEK
TEHLASSSEDSTTPSAQTQEK	3	HexNAc(1)Hex(1)NeuAc(1) % 656.2276	TEHLASSSEDstTPSAQTQEK
TFVLSALQPSPTHSSSNTQR	3	HexNAc(1)Hex(1)NeuAc(1) % 656.2276	TFVLSALQPsPTHSSSNTQR
TVLTPATNHMGNVTFTIPANR	3	HexNAc(4)Hex(1)NeuAc(2) % 1556.5611	TVLTPAtNHMGNVTFTIPANR
TVLTPATNHMGNVTFTIPANR	3	HexNAc(4)Hex(4) % 1460.5288	TVLtPAtNHMGNVTFTIPANR

Supplementary Table S4. List of O-glycoPSMs identified in a complex glycopeptide mixture by UVPD (8 pulses, 8mJ/pulse) in a standard MS1 range (400-2000 *m/z*) using *b*,*y* ions.

Supplementary Table S5. List of O-glycoPSMs identified in a complex glycopeptide mixture by sceHCD in a standard MS1 range (400-2000 *m/z*) using *b*,*y* ions. Table continues on the next page

Peptide	Charge	Total Glycan Composition	MSFragger Localization	Peptide	Charge	Total Glycan Composition	MSFragger Localization
AAAPNTPK	2	HexNAc(1)Hex(1)NeuAc(1) % 656.2276	AAAPNtPK	HYTNPSQDVTVPCPVPSTPPTPSPSTPPTPSPSCCHPR	4	HexNAc(5)Hex(3)NeuAc(2) % 2083.7462	HYTNPSQDVTVPCPVPSTPPtPSPSTPPTPSPSCCHPR
AVGDKLPECEAGATLINEQWLLTTAK	4	HexNAc(5)Hex(3)NeuAc(1) % 1792.6507	AVGDKLPECEAGAtLINEQWLLTTAK	HYTNPSQDVTVPCPVPSTPPTPSPSTPPTPSPSCCHPR	4	HexNAc(5)Hex(3)NeuAc(2) % 2083.7462	HYTNPSQDVTVPCPVPSTPPtPsPSTPPTPSPSCCHPR
AVGDKLPECEAGATLINEQWLLTTAK	4	HexNAc(4)Hex(1)NeuAc(4) % 2138.7520	AVGDKLPECEAGAtLINEQWLLTTAK	HYTNPSQDVTVPCPVPSTPPTPSPSTPPTPSPSCCHPR	4	HexNAc(5)Hex(4)NeuAc(2) % 2245.7990	HYTNPSQDVTVPCPVPSTPPtPSPSTPPTPSPSCCHPR
EETTVSPPHTSMAPAQDEER	3	HexNAc(4)Hex(4)NeuAc(4) % 2624.9104	EETTVsPPHTSMAPAQDEER	HYTNPSQDVTVPCPVPSTPPTPSPSTPPTPSPSCCHPR	4	HexNAc(4)Hex(3)NeuAc(2) % 1880.6668	HYTNPSQDVTVPCPVPstPPTPSPSTPPTPSPSCCHPR
EETTVSPPHTSMAPAQDEER	4	HexNAc(4)Hex(4)NeuAc(4) % 2624.9104	EEttVSPPHTSMAPAQDEER	HYTNPSQDVTVPCPVPSTPPTPSPSTPPTPSPSCCHPR	4	HexNAc(6)Hex(3)NeuAc(3) % 2577.9209	HYTNPSQDVTVPCPVPstPPTPSPSTPPTPSPSCCHPR
EETTVSPPHTSMAPAQDEER	3	HexNAc(4)Hex(4)NeuAc(4) % 2624.9104	EETTVsPPHTSMAPAQDEER	HYTNPSQDVTVPCPVPSTPPTPSPSTPPTPSPSCCHPR	5	HexNAc(5)Hex(4)NeuAc(4) % 2827.9898	HYTNPSQDVTVPCPVPstPPTPSPSTPPTPSPSCCHPR
EETTVSPPHTSMAPAQDEER	3	HexNAc(4)Hex(4)NeuAc(4) % 2624.9104	EETTVSPPHTsMAPAQDEER	HYTNPSQDVTVPCPVPSTPPTPSPSTPPTPSPSCCHPR	5	HexNAc(5)Hex(3)NeuAc(2) % 2083.7462	HYTNPSQDVTVPCPVPstPPTPSPSTPPTPSPSCCHPR
EETTVSPPHTSMAPAQDEER	3	HexNAc(2)Hex(2)NeuAc(3) % 1603.5506	EETtVSPPHTSMAPAQDEER	HYTNPSQDVTVPCPVPSTPPTPSPSTPPTPSPSCCHPR	4	HexNAc(5)Hex(4)NeuAc(4) % 2827.9898	HYTNPSQDVTVPCPVPstPPTPSPSTPPTPSPSCCHPR
EETTVSPPHTSMAPAQDEER	3	HexNAc(3)Hex(3)NeuAc(3) % 1968.6828	EEttVSPPHTSMAPAQDEER	HYTNPSQDVTVPCPVPSTPPTPSPSTPPTPSPSCCHPR	4	HexNAc(5)Hex(3)NeuAc(3) % 2374.8416	HYTNPSQDVTVPCPVPstPPTPSPSTPPTPSPSCCHPR
EETTVSPPHTSMAPAQDEER	3	HexNAc(3)Hex(3)NeuAc(3) % 1968.6828	EEttVsPPHTSMAPAQDEER	HYTNPSQDVTVPCPVPSTPPTPSPSTPPTPSPSCCHPR	5	HexNAc(5)Hex(4)NeuAc(3) % 2536.8944	HYTNPSQDVTVPCPVPstPPTPSPSTPPTPSPSCCHPR
EETTVSPPHTSMAPAQDEER	3	HexNAc(3)Hex(3)NeuAc(3) % 1968.6828	EETtVSPPHTSMAPAQDEER	HYTNPSQDVTVPCPVPSTPPTPSPSTPPTPSPSCCHPR	4	HexNAc(5)Hex(4)NeuAc(3) % 2536.8944	HYTNPSQDVTVPCPVPstPPTPSPSTPPTPSPSCCHPR
EETTVSPPHTSMAPAQDEER	3	HexNAc(3)Hex(1)NeuAc(2) % 1353.4818	EEtTVSPPHTSMAPAQDEER	HYTNPSQDVTVPCPVPSTPPTPSPSTPPTPSPSCCHPR	5	HexNAc(5)Hex(3)NeuAc(3) % 2374.8416	HYTNPSQDVTVPCPVPstPPTPSPSTPPTPSPSCCHPR
EETTVSPPHTSMAPAQDEER	3	HexNAc(2)Hex(2)NeuAc(2) % 1312.4552	EETTVsPPHTSMAPAQDEER	HYTNPSQDVTVPCPVPSTPPTPSPSTPPTPSPSCCHPR	4	HexNAc(3)Hex(2)NeuAc(1) % 1224.4392	HYTNPSQDVTVPCPVPSTPPtPSPSTPPTPSPSCCHPR
EETTVSPPHTSMAPAQDEER	3	HexNAc(2)Hex(2)NeuAc(3) % 1603.5506	EETTVsPPHTSMAPAQDEER	HYTNPSQDVTVPCPVPSTPPTPSPSTPPTPSPSCCHPR	4	HexNAc(4)Hex(3)NeuAc(2) % 1880.6668	HYTNPSQDVTVPCPVPstPPTPSPSTPPTPSPSCCHPR
EETTVSPPHTSMAPAQDEER	3	HexNAc(2)Hex(2)NeuAc(2) % 1312.4552	EETTVsPPHTSMAPAQDEER	HYTNPSQDVTVPCPVPSTPPTPSPSTPPTPSPSCCHPR	5	HexNAc(4)Hex(4)NeuAc(3) % 2333.8150	HYTNPSQDVTVPCPVPstPPTPSPSTPPTPSPSCCHPR
EETTVSPPHTSMAPAQDEER	3	HexNAc(2)Hex(2)NeuAc(4) % 1894.6461	EEttVSPPHTSMAPAQDEER	HYTNPSQDVTVPCPVPSTPPTPSPSTPPTPSPSCCHPR	4	HexNAc(4)Hex(4)NeuAc(3) % 2333.8150	HYTNPSQDVTVPCPVPstPPTPSPSTPPTPSPSCCHPR
EETTVSPPHTSMAPAQDEER	3	HexNAc(2)Hex(2)NeuAc(3) % 1603.5506	EETTVsPPHTSMAPAQDEER	HYTNPSQDVTVPCPVPSTPPTPSPSTPPTPSPSCCHPR	4	HexNAc(4)Hex(4)NeuAc(4) % 2624.9104	HYTNPSQDVTVPCPVPsTPPTPSPSTPPTPSPSCCHPR
EETTVSPPHTSMAPAQDEER	3	HexNAc(2)Hex(2)NeuAc(2) % 1312.4552	EEttVsPPHTSMAPAQDEER	HYTNPSQDVTVPCPVPSTPPTPSPSTPPTPSPSCCHPR	4	HexNAc(5)Hex(5)NeuAc(4) % 2990.0426	HYTNPSQDVTVPCPVPstPPTPSPSTPPTPSPSCCHPR
EGTPAPFGDQSHPEPESWNAAQHCQQDR	4	HexNAc(1)Hex(1)NeuAc(2) % 947.3230	EGtPAPFGDQSHPEPESWNAAQHCQQDR	HYTNPSQDVTVPCPVPSTPPTPSPSTPPTPSPSCCHPR	4	HexNAc(5)Hex(4)NeuAc(4) % 2827.9898	HYTNPSQDVTVPCPVPstPPTPSPSTPPTPSPSCCHPR
EPAPTAPK	2	HexNAc(1)Hex(1)NeuAc(1) % 656.2276	EPAPtAPK	HYTNPSQDVTVPCPVPSTPPTPSPSTPPTPSPSCCHPR	4	HexNAc(5)Hex(3)NeuAc(3) % 2374.8416	HYTNPSQDVTVPCPVPstPPTPSPSTPPTPSPSCCHPR
EPAPTTPK	2	HexNAc(2)Hex(2)NeuAc(2) % 1312.4552	EPAPtTPK	HYTNPSQDVTVPCPVPSTPPTPSPSTPPTPSPSCCHPR	4	HexNAc(4)Hex(2)NeuAc(2) % 1718.6140	HYTNPSQDVTVPCPVPstPPTPSPSTPPTPSPSCCHPR
EPAPTTPK	2	HexNAc(1)Hex(1)NeuAc(1) % 656.2276	EPAPtTPK	HYTNPSQDVTVPCPVPSTPPTPSPSTPPTPSPSCCHPR	5	HexNAc(4)Hex(4)NeuAc(4) % 2624.9104	HYTNPSQDVTVPCPVPstPPTPSPSTPPTPSPSCCHPR
EPAPTTPK	2	HexNAc(1)Hex(1)NeuAc(1) % 656.2276	EPAPtTPK	HYTNPSQDVTVPCPVPSTPPTPSPSTPPTPSPSCCHPR	4	HexNAc(5)Hex(5)NeuAc(4) % 2990.0426	HYTNPSQDVTVPCPVPstPPTPSPSTPPTPSPSCCHPR
EPAPTTPK	2	HexNAc(1)Hex(1)NeuAc(1) % 656.2276	EPAPtTPK	HYTNPSQDVTVPCPVPSTPPTPSPSTPPTPSPSCCHPR	4	HexNAc(4)Hex(3)NeuAc(3) % 2171.7622	HYTNPSQDVTVPCPVPstPPTPSPSTPPTPSPSCCHPR
EPAPTTPK	2	HexNAc(1)Hex(1)NeuAc(1) % 656.2276	EPAPtTPK	HYTNPSQDVTVPCPVPSTPPTPSPSTPPTPSPSCCHPR	4	HexNAc(4)Hex(3)NeuAc(3) % 2171.7622	HYTNPSQDVTVPCPVPstPPTPSPSTPPTPSPSCCHPR
EPAPTTPKEPAPTTPK	3	HexNAc(3)Hex(3)NeuAc(3) % 1968.6828	EPAPtTPKEPAPTTPK	HYTNPSQDVTVPCPVPSTPPTPSPSTPPTPSPSCCHPR	4	HexNAc(5)Hex(4)NeuAc(3) % 2536.8944	HYTNPSQDVTVPCPVPstPPtPsPstPPTPSPSCCHPR
EPAPTTPKKPAPK	4	HexNAc(2)Hex(2)NeuAc(2) % 1312.4552	EPAPtTPKKPAPK	HYTNPSQDVTVPCPVPSTPPTPSPSTPPTPSPSCCHPR	4	HexNAc(4)Hex(4)NeuAc(4) % 2624.9104	HYTNPSQDVTVPCPVPstPPTPSPSTPPTPSPSCCHPR
EPAPTTPKKPAPK	3	HexNAc(2)Hex(2)NeuAc(2) % 1312.4552	EPAPttPKKPAPK	HYTNPSQDVTVPCPVPSTPPTPSPSTPPTPSPSCCHPR	4	HexNAc(4)Hex(4)NeuAc(3) % 2333.8150	HYTNPSQDVTVPCPVPSTPPtPSPSTPPTPSPSCCHPR
EPAPTTPKKPAPTTPK	4	HexNAc(3)Hex(3)NeuAc(3) % 1968.6828	EPAPttPKKPAPTTPK	HYTNPSQDVTVPCPVPSTPPTPSPSTPPTPSPSCCHPR	4	HexNAc(4)Hex(4)NeuAc(5) % 2916.0059	HYTNPSQDVTVPCPVPstPPTPSPSTPPTPSPSCCHPR
EPAPTTTKEPAPTTPK	3	HexNAc(3)Hex(3)NeuAc(3) % 1968.6828	EPAPtTTKEPAPTTPK	HYTNPSQDVTVPCPVPSTPPTPSPSTPPTPSPSCCHPR	4	HexNAc(3)Hex(2)NeuAc(2) % 1515.5346	HYTNPSQDVTVPCPVPstPPTPSPSTPPTPSPSCCHPR
EPGVPTTKTPAATKPEMTTTAK	4	HexNAc(4)Hex(4)NeuAc(4) % 2624.9104	EPGVPttKTPAATKPEMTTTAK	HYTNPSQDVTVPCPVPSTPPTPSPSTPPTPSPSCCHPR	4	HexNAc(3)Hex(3)NeuAc(3) % 1968.6828	HYTNPSQDVTVPCPVPsTPPTPSPSTPPTPSPSCCHPR
ETAPTTPK	2	HexNAc(1)Hex(1)NeuAc(1) % 656.2276	ETAPtTPK	HYTNPSQDVTVPCPVPSTPPTPSPSTPPTPSPSCCHPR	4	HexNAc(3)Hex(3)NeuAc(4) % 2259.7783	HYTNPSQDVTVPCPVPstPPTPSPSTPPTPSPSCCHPR
ETTCSKESNEELTESCETK	3	HexNAc(2)Hex(2)NeuAc(1) % 1021.3598	ETtCsKEsNEELTESCETK	HYTNPSQDVTVPCPVPSTPPTPSPSTPPTPSPSCCHPR	4	HexNAc(4)Hex(4)NeuAc(4) % 2624.9104	HYTNPSQDVTVPCPVPstPPtPSPSTPPTPSPSCCHPR
FSEFWDLDPEVRPTSAVAA	2	HexNAc(1)Hex(1)NeuAc(2) % 947.3230	FsEFWDLDPEVRPTSAVAA	HYTNPSQDVTVPCPVPSTPPTPSPSTPPTPSPSCCHPR	4	HexNAc(5)Hex(3)NeuAc(3) % 2374.8416	HYtNPsQDVtVPCPVPSTPPTPSPSTPPTPSPSCCHPR
FSVATQTCQITPAEGPVVTAQYDCLGCVHPISTQSPDLEPILR	4	HexNAc(2)Hex(2)NeuAc(2) % 1312.4552	FSVATQTCQItPAEGPVVtAQYDCLGCVHPIstQSPDLEPILR	HYTNPSQDVTVPCPVPSTPPTPSPSTPPTPSPSCCHPR	4	HexNAc(5)Hex(3)NeuAc(4) % 2665.9370	HYtNPsQDVtVPCPVPSTPPTPSPSTPPTPSPSCCHPR
GTAPTTLK	2	HexNAc(1)Hex(1)NeuAc(1) % 656.2276	GtAPTTLK	HYTNPSQDVTVPCPVPSTPPTPSPSTPPTPSPSCCHPR	4	HexNAc(5)Hex(2)NeuAc(2) % 1921.6933	HYTNPSQDVTVPCPVPstPPTPSPSTPPTPSPSCCHPR
GVAHNNLMAMAQETGDNLYWGSVTGSQSNAVSPTPAPR	3	HexNAc(1)Hex(1)NeuAc(1) % 656.2276	GVAHNNLMAMAQEtGDNLYWGSVTGSQSNAVSPTPAPR	HYTNSSQDVTVPCR	3	HexNAc(3)Hex(3)NeuAc(4) % 2259.7783	HYtNSSQDVTVPCR
GVAHNNLMAMAQETGDNLYWGSVTGSQSNAVSPTPAPR	3	HexNAc(1)Hex(1)NeuAc(2) % 947.3230	GVAHNNLMAMAQEtGDNLYWGSVTGSQSNAVSPTPAPR	HYTNSSQDVTVPCR	3	HexNAc(3)Hex(2)NeuAc(4) % 2097.7254	HYtNSSQDVTVPCR
HYTNPSQDVTVPCPVPSTPPTPSPSTPPTPSPSCCHPR	4	HexNAc(5)Hex(5)NeuAc(1) % 2116.7564	HYTNPSQDVTVPCPVPstPPTPSPSTPPTPSPSCCHPR	HYTNSSQDVTVPCR	3	HexNAc(3)Hex(3)NeuAc(5) % 2550.8737	HYtNSSQDVTVPCR
HYTNPSQDVTVPCPVPSTPPTPSPSTPPTPSPSCCHPR	4	HexNAc(5)Hex(4)NeuAc(1) % 1954.7036	HYTNPSQDVTVPCPVPstPPTPSPSTPPTPSPSCCHPR	IGEIKEETTVSPPHTSMAPAQDEER	4	HexNAc(4)Hex(4)NeuAc(4) % 2624.9104	IGEIKEETTVsPPHTSMAPAQDEER
HYTNPSQDVTVPCPVPSTPPTPSPSTPPTPSPSCCHPR	4	HexNAc(5)Hex(2) % 1339.5025	HYTNPSQDVTVPCPVPsTPPTPSPSTPPTPSPSCCHPR	IGEIKEETTVSPPHTSMAPAQDEER	4	HexNAc(3)Hex(3)NeuAc(3) % 1968.6828	IGEIKEEttVsPPHTSMAPAQDEER
HYTNPSQDVTVPCPVPSTPPTPSPSTPPTPSPSCCHPR	4	HexNAc(5)Hex(4)NeuAc(2) % 2245.7990	HYTNPSQDVTVPCPVPSTPPtPsPSTPPTPSPSCCHPR	ITTAKPINPRPSLPPNSDTSK	4	HexNAc(2)Hex(2)NeuAc(2) % 1312.4552	ITtAKPINPRPSLPPNSDTSK
HYTNPSQDVTVPCPVPSTPPTPSPSTPPTPSPSCCHPR	4	HexNAc(6)Hex(3)NeuAc(2) % 2286.8255	HYTNPSQDVTVPCPVPSTPPtPsPSTPPTPSPSCCHPR	LAILPASAPPATSNPDPAVSR	3	HexNAc(3)Hex(3)NeuAc(3) % 1968.6828	LAILPAsAPPATSNPDPAVSR
HYTNPSQDVTVPCPVPSTPPTPSPSTPPTPSPSCCHPR	4	HexNAc(5)Hex(5)NeuAc(3) % 2698.9472	HYTNPSQDVTVPCPVPstPPTPSPSTPPTPSPSCCHPR	LHVPLMPAQPAPPKPQPTTR	4	HexNAc(2)Hex(2)NeuAc(2) % 1312.4552	LHVPLMPAQPAPPKPQPttR
HYTNPSQDVTVPCPVPSTPPTPSPSTPPTPSPSCCHPR	4	HexNAc(6)Hex(2)NeuAc(1) % 1833.6773	HYTNPSQDVTVPCPVPSTPPtPSPSTPPTPSPSCCHPR	LHVPLMPAQPAPPKPQPTTRTPPQSQTPGALPAK	5	HexNAc(3)Hex(3)NeuAc(3) % 1968.6828	LHVPLMPAQPAPPKPQPttRtPPQsQtPGALPAK
HYTNPSQDVTVPCPVPSTPPTPSPSTPPTPSPSCCHPR	4	HexNAc(5)Hex(4)NeuAc(4) % 2827.9898	HYTNPSQDVTVPCPVPstPPTPSPSTPPTPSPSCCHPR	LHVPLMPAQPAPPKPQPTTRTPPQSQTPGALPAK	5	HexNAc(4)Hex(4)NeuAc(4) % 2624.9104	LHVPLMPAQPAPPKPQPttRtPPQsQtPGALPAK
HYTNPSQDVTVPCPVPSTPPTPSPSTPPTPSPSCCHPR	4	HexNAc(5)Hex(2)NeuAc(1) % 1630.5979	HYTNPSQDVTVPCPVPsTPPTPSPSTPPTPSPSCCHPR	LHVPLMPAQPAPPKPQPTTRTPPQSQTPGALPAK	5	HexNAc(3)Hex(3)NeuAc(3) % 1968.6828	LHVPLMPAQPAPPKPQPttRtPPQsQtPGALPAK
HYTNPSQDVTVPCPVPSTPPTPSPSTPPTPSPSCCHPR	4	HexNAc(6)Hex(3)NeuAc(3) % 2577.9209	HYTNPSQDVTVPCPVPstPPTPSPSTPPTPSPSCCHPR	LHVPLMPAQPAPPKPQPTTRTPPQSQTPGALPAK	4	HexNAc(3)Hex(3)NeuAc(3) % 1968.6828	LHVPLMPAQPAPPKPQPttRtPPQsQTPGALPAK
HYTNPSQDVTVPCPVPSTPPTPSPSTPPTPSPSCCHPR	5	HexNAc(5)Hex(4)NeuAc(3) % 2536.8944	HYTNPSQDVTVPCPVPstPPTPSPSTPPTPSPSCCHPR	LHVPLMPAQPAPPKPQPTTRTPPQSQTPGALPAK	5	HexNAc(3)Hex(3)NeuAc(4) % 2259.7783	LHVPLMPAQPAPPKPQPttRtPPQsQtPGALPAK
HYTNPSQDVTVPCPVPSTPPTPSPSTPPTPSPSCCHPR	4	HexNAc(5)Hex(4)NeuAc(3) % 2536.8944	HYTNPSQDVTVPCPVPstPPTPSPSTPPTPSPSCCHPR	LPDATPTELAK	2	HexNAc(1)Hex(1)NeuAc(1) % 656.2276	LPDAtPTELAK

Supplementary Table S5 (continue). List of O-glycoPSMs identified in a complex glycopeptide mixture by sceHCD in a standard MS1 range (400-2000 *m/z*) using *b*,*y* ions.

Peptide	Charge	Total Glycan Composition	MSFragger Localization
LTPTTPEK	2	HexNAc(2)Hex(2)NeuAc(2) % 1312.4552	LTPtTPEK
LVNEVTEFAKTCVADESAENCDK	3	HexNAc(4)Hex(3)NeuAc(1) % 1589.5714	LVNEVtEFAKTCVADESAENCDK
NQVSLTCLVKGFYPSDIAVEWESNGQPENNYK	4	HexNAc(6)Hex(3)NeuAc(3) % 2577.9209	NQVsLtCLVKGFYPSDIAVEWESNGQPENNYK
RLAILPASAPPATSNPDPAVSR	3	HexNAc(3)Hex(3)NeuAc(3) % 1968.6828	RLAILPAsAPPATSNPDPAVSR
SCDTPPPCPR	2	HexNAc(1)Hex(1)NeuAc(2) % 947.3230	sCDTPPPCPR
SCDTPPPCPR	2	HexNAc(1)Hex(1)NeuAc(2) % 947.3230	sCDTPPPCPR
SHCIAEVENDEMPADLPSLAADFVESK	3	HexNAc(3)Hex(2)NeuAc(2) % 1515.5346	SHCIAEVENDEMPADLPsLAADFVESK
SPDESTPELSAEPTPK	2	HexNAc(2)Hex(2)NeuAc(2) % 1312.4552	SPDEStPELSAEPTPK
SPDESTPELSAEPTPK	2	HexNAc(1)Hex(1)NeuAc(1) % 656.2276	SPDEStPELSAEPTPK
SSTTKPPFKPHGSR	4	HexNAc(1)Hex(1)NeuAc(1) % 656.2276	SSTtKPPFKPHGSR
SSTTKPPFKPHGSR	3	HexNAc(1)Hex(1) % 365.1322	SStTKPPFKPHGSR
SSTTKPPFKPHGSR	3	HexNAc(1) % 203.0794	SStTKPPFKPHGSR
SSTTKPPFKPHGSR	4	HexNAc(2)Hex(2)NeuAc(2) % 1312.4552	SSttKPPFKPHGSR
SSTTKPPFKPHGSR	4	HexNAc(1)Hex(1)NeuAc(1) % 656.2276	SSttKPPFKPHGSR
SVTCHVKHYTNPSQDVTVPCPVPSTPPTPSPSTPPTPSPSCCHPR	4	HexNAc(2)Hex(2)NeuAc(3) % 1603.5506	SVtCHVKHYtNPsQDVtVPCPVPSTPPTPSPSTPPTPSPSCCHPR
TEHLASSSEDSTTPSAQTQEK	3	HexNAc(2)Hex(2)NeuAc(3) % 1603.5506	TEHLASSSEDsTTPSAQTQEK
TEHLASSSEDSTTPSAQTQEK	3	HexNAc(2)Hex(2)NeuAc(4) % 1894.6461	tEHLASSSEDSTTPSAQTQEK
TEHLASSSEDSTTPSAQTQEK	3	HexNAc(1)Hex(1) % 365.1322	TEHLASSSEDstTPSAQTQEK
TEHLASSSEDSTTPSAQTQEK	3	HexNAc(2)Hex(2)NeuAc(3) % 1603.5506	tEHLASSSEDSTTPSAQTQEK
TEHLASSSEDSTTPSAQTQEK	3	HexNAc(2)Hex(2)NeuAc(2) % 1312.4552	tEHLASSSEDSTTPSAQTQEK
TEHLASSSEDSTTPSAQTQEK	3	HexNAc(2)Hex(2)NeuAc(3) % 1603.5506	tEHLASSSEDSTTPSAQTQEK
TEHLASSSEDSTTPSAQTQEK	3	HexNAc(2)Hex(2)NeuAc(2) % 1312.4552	TEHLAsSSEDSTTPSAQTQEK
TEHLASSSEDSTTPSAQTQEK	3	HexNAc(1)Hex(1)NeuAc(2) % 947.3230	TEHLASSSEDsTTPSAQTQEK
TEHLASSSEDSTTPSAQTQEK	3	HexNAc(1)Hex(1)NeuAc(2) % 947.3230	TEHLASSSEDSTTPSAQtQEK
TEHLASSSEDSTTPSAQTQEK	3	HexNAc(1)Hex(1)NeuAc(2) % 947.3230	tEHLASSSEDSTTPSAQTQEK
TEHLASSSEDSTTPSAQTQEK	3	HexNAc(2)Hex(2)NeuAc(3) % 1603.5506	TEHLAsSSEDSTTPSAQTQEK
TEHLASSSEDSTTPSAQTQEK	3	HexNAc(2)Hex(2)NeuAc(2) % 1312.4552	TEHLASSSEDStTPSAQTQEK
TEHLASSSEDSTTPSAQTQEK	3	HexNAc(1)Hex(1)NeuAc(1) % 656.2276	tEHLASSSEDSTTPSAQTQEK
TEHLASSSEDSTTPSAQTQEK	3	HexNAc(2)Hex(2)NeuAc(3) % 1603.5506	TEHLASSSEDSTTPsAQTQEK
TEHLASSSEDSTTPSAQTQEK	3	HexNAc(1)Hex(1)NeuAc(2) % 947.3230	TEHLASSSEDSTTPsAQTQEK
TEHLASSSEDSTTPSAQTQEK	3	HexNAc(2)Hex(2)NeuAc(2) % 1312.4552	TEHLASSsEDSTTPSAQTQEK
TEHLASSSEDSTTPSAQTQEK	3	HexNAc(1)Hex(1)NeuAc(1) % 656.2276	TEHLASSSEDsttPSAQTQEK
TEHLASSSEDSTTPSAQTQEK	3	HexNAc(1)Hex(1)NeuAc(1) % 656.2276	TEHLASSSEDSTtPSAQTQEK
TEHLASSSEDSTTPSAQTQEK	3	HexNAc(2)Hex(2)NeuAc(3) % 1603.5506	TEHLASSSEDstTPSAQTQEK
TEHLASSSEDSTTPSAQTQEK	3	HexNAc(1)Hex(1)NeuAc(2) % 947.3230	TEHLASssEDSTTPSAQTQEK
TEHLASSSEDSTTPSAQTQEK	3	HexNAc(1)Hex(1)NeuAc(1) % 656.2276	TEHLASSSEDStTPSAQTQEK
TEHLASSSEDSTTPSAQTQEK	3	HexNAc(2)NeuAc(1) % 697.2542	tEHLASSSEDSTTPSAQTQEK
TFVLSALQPSPTHSSSNTQR	3	HexNAc(1)Hex(1)NeuAc(1) % 656.2276	tFVLSALQPSPTHSSSNTQR
TFVLSALQPSPTHSSSNTQR	3	HexNAc(2)Hex(2)NeuAc(2) % 1312.4552	TFVLSALQPsPTHSSSNTQR
TFVLSALQPSPTHSSSNTQR	2	HexNAc(1)Hex(1)NeuAc(1) % 656.2276	TFVLsALQPSPTHSSSNTQR
TFVLSALQPSPTHSSSNTQR	3	HexNAc(1)Hex(1)NeuAc(1) % 656.2276	TFVLSALQPsPtHsssNTQR
TITTTEIMNKPEETAKPK	4	HexNAc(2)Hex(2)NeuAc(2) % 1312.4552	TItTTEIMNKPEETAKPK
TPAATKPEMTTTAK	3	HexNAc(2)Hex(2)NeuAc(2) % 1312.4552	TPAAtKPEMTTTAK
TPPQSQTPGALPAK	2	HexNAc(1)Hex(1)NeuAc(1) % 656.2276	TPPQsQTPGALPAK
VATTVISK	2	HexNAc(2)Hex(2)NeuAc(4) % 1894.6461	VAtTVISK
VATTVISK	2	HexNAc(2)Hex(2)NeuAc(3) % 1603.5506	VATTVIsK
VLAKPTPK	3	HexNAc(1)Hex(1)NeuAc(1) % 656.2276	VLAKPtPK
VYACEVTHQGLSSPVTK	4	HexNAc(4)Hex(2)NeuAc(6) % 2882.9956	VYACEVtHQGLSSPVTK
WEYCDVSACSAQDVAYPEESPTEPSTK	3	HexNAc(1)Hex(1)NeuAc(2) % 947.3230	WEYCDVsACsAQDVAYPEESPTEPSTK
